# Diagnostic efficacy of cone beam computed tomography in paediatric dentistry: a systematic review

**DOI:** 10.1007/s40368-019-00504-x

**Published:** 2019-12-19

**Authors:** K. Horner, S. Barry, M. Dave, C. Dixon, A. Littlewood, C. L. Pang, A. Sengupta, V. Srinivasan

**Affiliations:** 1 Division of Dentistry, School of Medical Sciences, Faculty of Biology, Medicine and Health, University of Manchester, Manchester Academic Health Science Centre, Coupland Building 3, Manchester, M13 9PL UK; 2 Dental Radiology, University Dental Hospital of Manchester, Manchester University NHS Foundation Trust, Manchester Academic Health Science Centre, Higher Cambridge Street, Manchester, M15 6FH UK; 3 Paediatric Dentistry, University Dental Hospital of Manchester, Manchester University NHS Foundation Trust, Manchester Academic Health Science Centre, Higher Cambridge Street, Manchester, M15 6FH UK; 4 Information Specialist, Cochrane Oral Health, Division of Dentistry, School of Medical Sciences, Faculty of Biology, Medicine and Health, University of Manchester, Manchester Academic Health Science Centre, Coupland Building 3, Manchester, M13 9PL UK; 5 Division of Imaging, Manchester Royal Infirmary, Manchester University NHS Foundation Trust, Manchester Academic Health Science Centre, Oxford Road, Manchester, M13 9WL UK

**Keywords:** Cone beam computed tomography, Radiography, dental, Paediatric dentistry, Diagnosis

## Abstract

**Purpose:**

To determine in which clinical situations it is indicated or contra-indicated to prescribe cone beam computed tomography (CBCT) for paediatric patients.

**Methods:**

Systematic review of in vivo paediatric research studies of diagnostic efficacy using CBCT, with supplementary searches for guideline documents on CBCT and for systematic reviews permitting inclusion of ex vivo and adult studies.

**Results:**

After screening, 190 publications were included, mostly case studies. No systematic reviews were found of in vivo paediatric research. Fourteen studies of diagnostic efficacy were identified. The supplementary searches found 18 guideline documents relevant to the review and 26 systematic reviews. The diagnostic efficacy evidence on CBCT was diverse and often of limited quality. There was ex vivo evidence for diagnostic accuracy being greater using CBCT than radiographs for root fractures. The multiplanar capabilities of CBCT are advantageous when localising dental structures for surgical planning. Patient movement during scanning is more common in children which could reduce diagnostic efficacy.

**Conclusions:**

No strong recommendations on CBCT are possible, except that it should not be used as a primary diagnostic tool for caries. Guidelines on use of CBCT in the paediatric age group should be developed cautiously, taking into account the greater radiation risk and the higher economic costs compared with radiography. CBCT should only be used when adequate conventional radiographic examination has not answered the question for which imaging was required. Clinical research in paediatric patients is required at the higher levels of diagnostic efficacy of CBCT.

**Electronic supplementary material:**

The online version of this article (10.1007/s40368-019-00504-x) contains supplementary material, which is available to authorized users.

## Introduction

Cone beam computed tomography (CBCT) is used for a wide variety of dental diagnostic uses, including in children and young people (Aps 2013). CBCT typically has a radiation dose one or more orders of magnitude greater than that for conventional radiography (Theodorakou et al. 2012; European Commission 2012; Ludlow et al. 2015). This is important in paediatric use because of the higher levels of risk associated with X-ray exposure in young age groups. This has stimulated efforts on justification and dose optimisation of CBCT in the paediatric context (Law et al. 2014; White et al. 2014; Oenning et al. 2018). In addition, the financial costs of using CBCT instead of, or in addition to, conventional imaging is likely to raise the overall costs of healthcare, unless its use leads to cost-savings elsewhere along the patient care pathway.

Radiation dose and risk are not primary determinants of whether or not to use a diagnostic X-ray technique. A fundamental principle of radiation protection, justification, requires that the potential benefits of its use outweigh the risk. Fryback and Thornbury (1991) devised a hierarchical model of diagnostic efficacy which conceptualises the benefits (Table 1). Evidence of efficacy at the lower levels does not guarantee that it exists at higher levels. Criteria for using an imaging modality should be based on evidence rather than opinion, ideally on a body of evidence at the higher levels of diagnostic efficacy with a low risk of bias.

**Table 1:** The hierarchical Model of diagnostic efficacy. Adapted from Fryback and Thornbury (1991)

Level	Example measures of analyses
Level 6: Societal efficacy	Economic evaluation/cost analysis/cost-effectiveness evaluation from a societal standpoint; cost per outcome change
Level 5: Patient outcome efficacy	e.g. proportion of patients improved pre-test to post-test; morbidity or procedures avoided after having test results
Level 4: Therapeutic efficacy	e.g. proportion of cases in which prospectively stated treatment plan changed pre-test to post-test
Level 3: Diagnostic thinking efficacy	e.g. difference in clinicians’ pre- and post-test diagnoses; change in percentage of cases in a series in which the image was judged to be “helpful” in making diagnosis
Level 2: Diagnostic accuracy efficacy	Sensitivity; specificity; predictive vales; diagnostic odds ratios; ROC curve analysis
Level 1: Technical efficacy	Spatial resolution; grey-scale; contrast–noise ratio; sharpness; Modulation Transfer Function (MTF); linear accuracy

There are a substantial number of guidelines published related to the clinical use of CBCT including referral guidelines, also known as “appropriateness criteria” and “selection criteria”. These were reviewed by Horner et al. (2015), but very little was found specifically on paediatric use of CBCT. The underlying reason for this systematic review was to address this deficiency. The aim was to determine in which clinical situations and paediatric age groups is it indicated or contra-indicated to prescribe CBCT. To achieve this aim, an overall review question was developed: “what are the indications and contra-indications for the use of cone beam computed tomography (CBCT) in the dental care of children and young people as part of diagnosis and management?”

## Materials and methods

### Protocol and registration

The protocol for this systematic review was registered on the International Prospective Register of Systematic Reviews (PROSPERO) and can be accessed at https://www.crd.york.ac.uk/PROSPERO/display_record.asp?ID=CRD42018109768.

### Eligibility criteria

#### Study designs

In vivo paediatric studies of diagnostic efficacy as defined by Fryback and Thornbury (1991) (Table 1).

Included:

- Systematic reviews of in vivo diagnostic efficacy studies.
- Primary studies of in vivo diagnostic efficacy (if not included in a systematic review).
- Narrative reviews, case series, case reports, surveys of clinical use of CBCT and other research study designs (observational studies; observer reliability studies) and guideline documents as secondary sources of information.

Excluded:

- Studies of technical efficacy (level 1, Table 1)
- Studies of any design for which the objectives were to evaluate treatments, in which the use of CBCT was simply as a diagnostic tool.
- Ex vivo*/*in vitro studies
- Animal studies.
- Research on orthodontic applications of CBCT, although flexibility was permitted if these had relevance to paediatric dentistry.
- Radiation dosimetry studies.

#### Participants

Children and young people (under 18 years) under care for any of six clinical contexts (caries, acute dental infections, dental trauma, dental anomalies, developmental disorders and pathological conditions). A seventh category of clinical context was added to encompass “other uses” of CBCT. We included studies that included both adults and children/young people at the same time if data for the latter group could be extracted. We excluded studies solely restricted to adults (18 years or over) unless the clinical context was judged to be clearly also applicable to children and young people.

#### Intervention

CBCT used for dental diagnostic purposes. We excluded studies using multislice (“medical”) computed tomography (CT) and CBCT equipment not designed for dental use.

#### Comparators

For diagnostic accuracy (level 2) studies (Table 1), a reference standard comparator was essential (surgical evidence; histopathological; microCT; other method judged to have sufficient validity). For studies at levels 2–6 (inclusive) of diagnostic efficacy, comparison with conventional dental radiography (intraoral, panoramic and cephalometric radiography), another imaging modality or other diagnostic test was expected. For research at the societal efficacy level (level 6), studies without a comparator were considered for inclusion on an individual basis.

#### Outcomes

For the use of CBCT in each of the six clinical contexts being studied, in comparison with the alternative imaging method(s) or, in the case of no comparator imaging, clinical assessment alone:

- Change in one or more measures of diagnostic accuracy.
- Change in diagnostic thinking, including clinicians’ confidence in their diagnosis or the perceived helpfulness of the imaging in reaching a diagnosis.
- Change in management decision(s), including clinicians’ confidence in their decision(s) or the perceived helpfulness of the imaging in making the decision(s).
- Change in patient outcome after the treatment of the condition.
- Change in costs, cost-utility, cost-effectiveness or other economic measure(s) of efficacy.

#### Setting

Studies in either a primary or a secondary dental healthcare setting.

#### Language

English language studies or at least an English abstract. Studies in other languages were considered for inclusion pragmatically if there was a means of translation within the review team.

### Information sources and searches

Literature search strategies were developed using medical subject headings (MeSH) and text words related to CBCT, children and young people, and dental diseases. Parts of this search strategy were adapted from Leclercq et al. (2013). Search details are presented in Tables 2 and 3.

**Table 2:** The databases searched for the systematic review

MEDLINE Ovid (inc ePub ahead of print, pre-indexed etc.)	1409	PROSPERO*	71
Embase Ovid	856	US National Institutes of Health Trials Registry (ClinicalTrials.gov)*	34
Proquest Dissertations and Theses	120	WHO International Clinical Trials Registry Platform*	4
Web of Science Conference Proceedings	12		

Date of search 08.10.2018. Dates of search coverage 1998–October 2018 except for those marked (*), for which searches of the whole database were made

Total number of papers retrieved: 2506—731 duplicates = 1775 used for this review

**Table 3:** Medline Ovid search strategy

1	exp “cone-beam computed tomography”/
2	(“cone-beam computed tomography” or “cone-beam CAT scan$” or “conebeam CT scan$” or “cone-beam CT” or “cone-beam computer-assisted tomography” or “cone-beam computeried tomography” or “cone-beam computed tomography”).mp.
3	(“volume CT” or “volume computed tomography” or “volumetric CT” or “volumetric computed tomography”).mp.
4	(“digital volumetric tomography” or “digital volume tomography”).mp.
5	(cbct or qcbct).mp.
6	or/1–5
7	exp dentistry/
8	exp tooth diseases/di, dg
9	(oral or dental or intra-oral or intraoral or dentist$).mp.
10	(caries or carious or (tooth adj3 decay) or (teeth adj3 decay)).mp.
11	((tooth or teeth or dental) adj5 (infect$ or diseas$ or trauma$ or injur$ or luxat$ or avuls$)).mp.
12	exp Mouth abnormalities/
13	(orthodontic$ or malocclusion or cleft$ or “open bite” or “deep bite” or ((tooth or teeth) adj crowd$) or “cross bite” or crossbite).mp.
14	or/7–13
15	exp Child/
16	adolescent/
17	(minors or minor or boy or boys or boyhood or girl$ or kid or kids or child or child$ or children$ or schoolchild$ or “school child$” or adolescen$ or juvenil$ or youth$ or teen$ or underage$ or pubescen$ or pediatric$ or paediatric$ or peadiatric$ or school).mp.
18	tooth, deciduous/
19	((tooth or teeth or dentition) adj3 (primary or milk or deciduous or baby)).mp.
20	or/15–19
21	6 and 14
22	20 and 21

Lines 15–20 of the search strategy were adapted from Leclercq et al. (2013). The Embase Ovid search strategy is not presented as it was essentially the same, with only minor differences in terms, e.g. for Embase Ovid, line 12 was “Mouth malformation/di” and line 18 was “Deciduous tooth/”

### Study selection

EndNote was used to compile the searches and de-duplicate references. Retrieved titles and abstracts were screened by pairs of review authors from the team independently to identify publications that potentially met the inclusion criteria. The screened lists were reviewed by a third team member who combined them into a single list. The full text of these potentially eligible studies was retrieved and independently assessed by two review team members. Disagreement over eligibility was resolved through discussion with a third reviewer. Studies were classified according to the six clinical contexts (plus “other uses”) and ten study design types (systematic review, the five levels of diagnostic efficacy, narrative reviews, case series/reports/surveys, other research study designs, clinical guidelines).

### Data collection process

Standardised forms were used to extract data from the included studies for assessment of study quality and evidence synthesis. For primary studies of diagnostic efficacy, two review authors extracted data independently and discrepancies were identified and resolved through discussion (with a third author where necessary). Extracted information included: study setting (primary or secondary care; study population, demographics and presenting characteristics; CBCT equipment used and operating parameters; comparator imaging (if used); study methodology; observer/rater profile; recruitment and study completion rates; outcomes; information for assessment of the risk of bias. For other study types, a specific form was used to record the relevant data. Case reports with fewer than five individual cases were not formally reviewed, but were collated to provide an indication of the uses to which CBCT has been applied.

### Risk of bias in individual studies

The two review authors independently assessed the risk of bias in systematic reviews and primary studies of diagnostic efficacy. The tools planned for critical appraisal are shown in Table 4. Disagreement was resolved by discussion and, if required, by involvement of a third reviewer.

**Table 4:** Critical appraisal tools planned for review of systematic reviews of diagnostic efficacy and primary studies of diagnostic efficacy arising from the main review search

Study type	Tool
Systematic reviews	AMSTAR-2 (Shea et al. 2017)
Diagnostic accuracy efficacy	QUADAS 2 (Whiting et al. 2011)
Diagnostic thinking efficacy	Modified QUADAS (Meads and Davenport 2009)
Therapeutic efficacy	Modified QUADAS (Meads and Davenport 2009)
Patient outcome efficacy	Cochrane Handbook for Systematic Reviews of Interventions, Version 5.1.0 (Higgins and Green 2011)
Societal efficacy	For studies including a patient outcome assessment, Consensus Health Economic Criteria (CHEC) list (Evers et al. 2005) For cost analysis studies without a patient outcome assessment, the proforma used by Christell et al. (2014) based on Drummond et al. (2005)

### Data synthesis

We undertook a systematic narrative synthesis to explore the relationship and findings both within and between the included studies. The purpose was to present clearly, for each clinical condition, information which allowed identification of indications and contra-indications for CBCT.

### Supplementary information

Following initial scoping searches, it became apparent that the eligibility criteria for the main search would be highly restrictive. Therefore, two supplementary searches for information relevant to the review were undertaken. The first identified existing clinical guidelines on the use of CBCT. The second was a broad search for systematic reviews on diagnostic efficacy using CBCT which included ex vivo*/*in vitro studies and in vivo studies of (or including) adult patients. These are described in Online Resources 1 and 2, respectively.

## Results

### Study selection

**Fig. 1:**
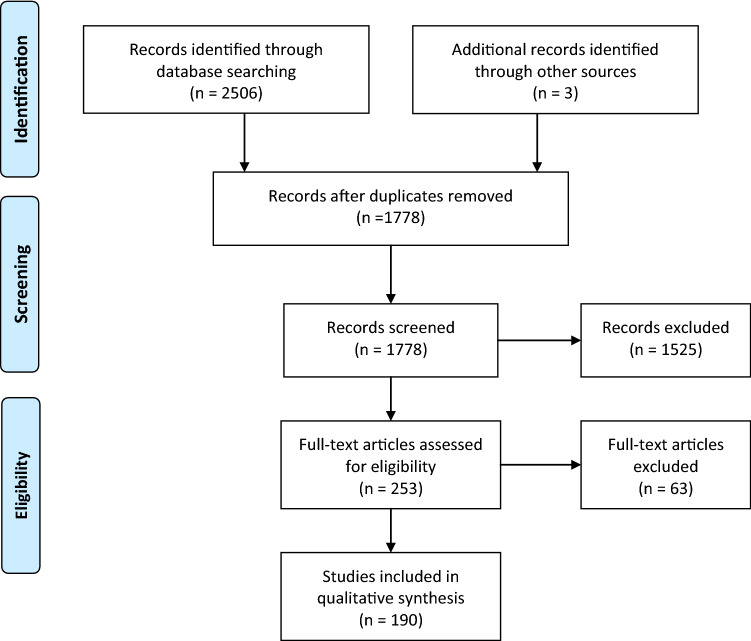
PRISMA flow chart (Moher et al. 2009) showing the flow of publications arising from the main search

Figure 1 shows the flow of the articles identified through our main search. One hundred and ninety publications were included, listed in Online Resource 3. Table 5 shows the allocation of the included studies according to the clinical context and the study type. Some studies fell into multiple clinical contexts, so the summed numbers in Table 5 exceed the 190 shown in Fig. 1. Publications were overwhelmingly in the case series, case report and survey category.

**Table 5:** Allocation of the included studies in the review, according to the clinical context and the study type

Study type	Caries	Acute dental infections	Dental trauma	Dental anomalies	Developmental disorders	Pathological conditions	Other uses
Systematic reviews of diagnostic efficacy	0	0	0	0	0	0	0
Diagnostic accuracy efficacy	1	0	0	1	0	1	1
Diagnostic thinking efficacy	0	0	1	4	1	5	0
Therapeutic efficacy	0	0	0	3	1	1	0
Patient outcome efficacy	0	0	0	0	0	0	0
Societal efficacy	0	0	0	2	0	0	0
Narrative reviews	4	3	4	4	3	4	4
Case series/case reports/surveys	3	4	11	65	15	29	26
Other research study designs	0	0	0	0	10	6	19
Clinical guidelines	1	1	1	1	1	1	3

No systematic review from the main search strategy satisfied the inclusion criteria. There were 14 primary research studies of diagnostic efficacy; for these, flexibility in the inclusion criteria was permitted. Four of these appeared in both Level 3 and Level 4 categories of diagnostic efficacy (Haney et al. 2010; Katheria et al. 2010; Botticelli et al. 2011; Wriedt et al. 2017). No patient outcome efficacy study was identified. The evidence from the 14 diagnostic efficacy studies is summarised in Table 6.

**Table 6:** Summary of findings from the fourteen diagnostic efficacy studies identified in the review

Clinical context(s)	Caries	Purpose of imaging	Diagnosis of proximal caries cavitation
Sansare et al. (2014)			
Diagnostic efficacy level	Level 2: diagnostic accuracy		
Imaging (index tests)	1: CBCT [Kodak 9000; small FoV; 0.07-mm voxel] 2: Bitewing radiography Two observers performed independent assessments of index tests, blinded to the true diagnosis		
Reference standard	Elective temporary tooth separation		
Patient sample description	34 adults for whom there was suspicion of cavitation on visual examination. Prevalence of condition: 61% cavitated Ages: 18–63 years. Gender: 17F/17M. Setting: secondary care healthcare facility in India		
Key outcomes	Using CBCT gave statistically significant greater sensitivity and accuracy. CBCT: Se = 75% and 79%, Sp = 77% and 77%, Acc = 76% and 78% Bitewing: Se = 46% and 42%, Sp = 84% and 87%, Acc = 61% and 59%		
Study strengths	Blinding of assessors to diagnostic truth Reference standard appears robust		
Study weaknesses	Adult population (weakness in the context of the review) Recruitment process unclear Only two assessors of images, both radiologists not dentists Unclear about the time interval between assessing CBCT and bitewing images		

Clinical context(s)	Dental trauma	Purpose of imaging	Diagnosis of horizontal root fracture position and angulation in permanent incisors
Bornstein et al. (2009)			
Diagnostic efficacy level	Level 3: diagnostic thinking efficacy		
Imaging (index tests)	1. CBCT [3 DX Accuitomo XYZ Tomograph; 4 cm × 4 cm FoV; 0.125 mm voxel] 2. Periapical radiograph (analogue; paralleling technique] 3. Occlusal radiograph (analogue)		
Reference standard	Not applicable		
Patient sample description	38 patients presenting as emergencies with single or multiple horizontal root fractures of permanent teeth, with 44 fractured maxillary incisors within this sample Ages: mean age 24 years (8–52 years), Gender: 12F/26 M, Setting: University-based secondary healthcare facility in Switzerland		
Key outcomes	Key outcome measure was fracture location on facial and palatal surfaces The fracture location on the palatal surface was more coronal on CBCT than on radiographs. In particular, a cervical fracture was more common on CBCT, potentially influencing management		
Study strengths	Consecutive patients Clearly described methods		
Study weaknesses	Retrospective study Selection bias possible: only cases with true-positive diagnoses on both CBCT and conventional imaging included Single observer performed the study assessments		

Clinical context(s)	Dental anomalies	Purpose of imaging	Localisation of unerupted and supernumerary teeth in the maxilla
Ziegler and Klimowicz (2013)			
Diagnostic efficacy level	Level 2: diagnostic accuracy		
Imaging (index tests)	1: CBCT [iCAT—model not specified. FoV size not specified; 0.3-mm voxel probably used, but not clear] 2: Intraoral or panoramic radiographs; localisation using the “magnification method” (no explanation of what this means) Three surgeons made preoperative assessments of images independently		
Reference standard	Intraoperative findings by surgeon		
Patient sample description	61 mixed age group patients Ages: mean age 15 years (9–57 years), gender: not specified, setting: secondary care healthcare facility in Norway, prior testing: unknown		
Key outcomes	Higher proportion of correct pre-operative localisation of bucco-palatal position using CBCT CBCT: 96.7% correct Radiographic “magnification method”: 39.3% correct		
Study strengths	Prospective study Surgical reference standard		
Study weaknesses	Recruitment process unclear Uncertain time gap between the index tests Inadequate conventional radiography (single panoramic or intraoral); the “magnification method” used is not explained; so unfair comparison against CBCT The index test assessments by the surgeons not clearly explained No statistics presented but “statistically significant” used in text Numerous text errors in the publication		

Clinical context(s)	Dental anomalies and pathological conditions	Purpose of imaging	Diagnosis and treatment planning of impacted maxillary canines, including diagnosis of root resorption in permanent incisors
Haney et al. (2010)			
Diagnostic efficacy level	Level 3: Diagnostic thinking efficacy and Level 4: Therapeutic efficacy		
Imaging (index tests)	1. CBCT: Hitachi MercuRay; CBWorks software used to produce ”3D” selected images for study 2. Panoramic radiograph 3. Occlusal radiograph 4. Two periapical radiographs Seven faculty members were assessors in the study: four orthodontists and three oral surgeons		
Reference standard	Not applicable		
Patient sample description	18 consecutive patients with impacted maxillary canines. sample included 25 canines with 7 bilateral impactions Ages: mean age 16.9 years (12.3–34.6 years), gender: 12F/6M, setting: University-based secondary healthcare facility in USA		
Key outcomes	Assessors make different decisions on aspects of diagnosis and treatment plans using CBCT for a minority of cases, e.g.: 16% disagreement for labiopalatal position of canine 50% disagreement when localising the cusp in the vertical dimension 36% agreement regarding root resorption between the two methods Clinicians’ confidence in the accuracy of diagnosis and the treatment plan was statistically greater using CBCT		
Study strengths	Prospective study on consecutive patient sample Full range of conventional radiographs available Seven assessors Statistical analyses clear		
Study weaknesses	Presentation of images as print-outs on paper Risk of recognition of cases by assessors; radiographs and CBCT images were viewed on same session, including repeat cases for reliability assessment, with no “washout” period CBCT images viewed only as “3D” reconstructions, unlike normal practice Combining observations and decisions of all assessors for data analysis “Confidence” in diagnosis and treatment plans measured together		

Clinical context(s)	Dental anomalies and pathological conditions	Purpose of imaging	Diagnosis and treatment planning of impacted and supernumerary teeth
Katheria et al. (2010)			
Diagnostic efficacy level	Level 3: Diagnostic thinking efficacy and level 4: therapeutic efficacy		
Imaging (index tests)	1. CBCT: 3D Sirona Galileos; FoV = full facial bone scan; voxel size not stated 2. Panoramic-like image and maxillary occlusal-like image synthesised from CBCT dataset (“Traditional Radiographs”, TR) Ten paediatric residents and ten paediatric dentists served as observers. Each observer viewed four cases only		
Reference standard	Not applicable		
Patient sample description	Eight patients’ radiographic records, each with one impacted canine or supernumerary tooth in the anterior maxilla. Ages: not specified: only “paediatric”, gender: not specified, setting: University-based secondary healthcare facility in USA		
Key outcomes	No significant difference in “pathology diagnosis” using TR or CBCT Significantly greater proportion of decisions on location of pathology classed as “correct” using CBCT compared with TR (but no information on how “correct” was identified) Significantly higher proportion of observer decisions that root resorption was present using CBCT compared with TR Significantly higher proportions of observer decisions on “usefulness of CBCT” in the “very useful” category for both diagnosis and treatment planning		
Study strengths	Multiple observers in the study Observers reviewed four cases using TR and four cases using CBCT, but not both imaging types of the same case, so no risk of recognition of case Missing information in the publication relevant to the review		
Study weaknesses	Retrospective design High risk of selection bias Small number of cases Radiographs synthesised from CBCT Missing information in the publication relevant to the review (e.g. patient data) Use of terms such as “correct diagnosis” in the absence of any diagnostic truth Combining observations and decisions of assessors for data analysis		

Clinical context(s)	Dental anomalies and pathological conditions	Purpose of imaging	Localisation and other imaging aspects of impacted maxillary canines
Alqerban et al. (2011)			
Diagnostic efficacy level	Level 3: diagnostic thinking efficacy		
Imaging (index tests)	1. CBCT: 3D Accuitomo-XYZ; FoV = 30 × 40 mm; voxel size = 0.125 mm 2. CBCT: Scanora 3D CBCT; FoV = 75 × 100 mm; voxel size = 0.2 mm 3. Panoramic radiograph: Cranex Tome (Soredex) Sample had either 1 or 2, but all had 3 Eleven assessors for study (3 experienced dental practitioners and 8 postgraduate (PG) students) The PG students only assessed a limited number of aspects of the imaging		
Reference standard	Not applicable		
Patient sample description	Sixty consecutive patients with impacted or ectopically erupting maxillary canines seeking orthodontic treatment Ages: Mean age 13.2 years (± 4.2 years); (6.3–28.9 years), Gender: 37F/23M, Setting: University-based secondary healthcare facility in Belgium		
Key outcomes	Greater agreement between observers for all variables was achieved when using CBCT Observers’ decisions based on CBCT and panoramic radiography were significantly different for: Canine location Detection of the presence or absence of root resorption of the lateral incisor Detection of the presence or absence of root resorption in the central incisor (Accuitomo group only) Severity of lateral incisor root resorption		
Study strengths	Consecutive patient sample Inter-observer agreement assessed thoroughly		
Study weaknesses	Retrospective Major weakness was no intraoral radiographs, only panoramic radiographs, so how could position and resorption be assessed properly from a single panoramic image? Only partial assessments by PG students No intra-observer repeatability assessment Combining observations and decisions of assessors for data analysis		

Clinical context(s)	Dental anomalies	Purpose of imaging	Diagnosis and treatment planning of impacted maxillary canines
Botticelli et al. (2011)			
Diagnostic efficacy level	Level 3: diagnostic thinking efficacy and Level 4: therapeutic efficacy		
Imaging (index tests)	1. CBCT: NewTom 3G (Quantitative Radiology s.r.l., Verona, Italy); FoV = not specified; Voxel size = not specified) 2. Conventional imaging: panoramic radiograph, periapical radiograph and lateral cephalogram Eight dentists acted as observers (3 specialists and 5 PG trainees: 2 at end of training and 3 early in training)		
Reference standard	Not applicable		
Patient sample description	Twenty-seven patients with 39 ectopic maxillary canines undergoing orthodontic treatment Ages: Mean age 11.8 years, Gender: 17F/10M, Setting: University-based secondary healthcare facility in Denmark		
Key outcomes	Observers’ decisions based on CBCT and conventional radiography were statistically significantly different for: Mesio-distal localization of the apex Vertical level of the clinical crown Overlap with the lateral incisor Labio-palatal position of the crown Labio-palatal position of the apex Root resorption of neighbouring incisor/s Treatment strategy (more observational strategy with conventional imaging; more interventional with CBCT) Treatment assessed as more difficult with CBCT Image quality (CBCT better) However, for all except treatment difficulty and image quality the majority of the decisions (≥ 64%) were the same when using CBCT or conventional radiography		
Study strengths	Prospective Comprehensive conventional radiographic series Eight observers Clear written and visual presentation of findings		
Study weaknesses	Lack of detail about conduct of index tests Images presented as Powerpoint presentations, with pre-selected CBCT images No intra-observer repeatability assessment Combining observations and decisions of assessors for data analysis		

Clinical context(s)	Dental anomalies	Purpose of imaging	For imaging in the context of three different clinical contexts and in four countries (cost analysis)
Christell et al. (2012a)			
Diagnostic efficacy level	Level 6: societal efficacy		
Imaging (index tests)	CBCT: Four different scanners in four different centres No comparator imaging NewTom 3G [Quantitative Radiology (QR),Verona, Italy] Scanora (Soredex, Helsinki, Finland) Accuitomo MCT-1 (Morita, Kyoto, Japan) NewTom CVT 9000 (QR)		
Reference standard	Not applicable		
Patient sample description	One hundred and sixty patients referred for a CBCT examination during one calendar year for one of the following: imaging of maxillary canines with eruption disturbances, of an area with tooth loss prior to implant treatment or of a lower third molar planned for removal (cost analysis) Ages: Means and age ranges of the twelve combinations of country/clinical indication all presented in the paper. A paediatric group is not separately presented, but forms most of the maxillary canine group in two settings. Gender: not specified, settings: Four University-based secondary care specialist centres, in Romania, Belgium, Sweden and Lithuania		
Key outcomes	Estimates for direct and indirect costs varied among the healthcare systems Estimates for direct and indirect costs varied according to clinical application Variation in direct costs was mainly owing to different capital costs Variation in indirect costs mainly owing to differences in examination fees Cost-efficacy established in one healthcare system might not be so in a different system		
Study strengths	Input of health economist to research team Consecutive patients		
Study weaknesses	No assessment of outcomes for patients No comparator imaging method (no incremental cost calculations) Patient’s or accompanying person’s average earnings used to calculate indirect costs, not real earnings		

Clinical context(s)	Dental anomalies	Purpose of imaging	Imaging of maxillary canines with eruption disturbances (cost analysis)
Christell et al. (2012b)			
Diagnostic efficacy level	Level 6: societal efficacy		
Imaging (index tests)	1. New imaging method: CBCT [Accuitomo (Morita, Kyoto, Japan); FoV not specified; voxel size not specified] + panoramic radiograph [Planmeca Pro Max, (Helsinki, Finland)] 2. Conventional imaging method: panoramic radiograph [Planmeca Pro Max, (Helsinki, Finland)] + intraoral radiographs [Planmeca Intra (Helsinki, Finland)] Both methods included one panoramic radiograph per examination. The new method based on a recorded mean of 1.4 CBCT examinations per examination. The conventional method based on a recorded mean of 2.9 intraoral radiographs per examination		
Reference standard	Not applicable		
Patient sample description	Forty-seven patients referred for examination of maxillary canines with eruption disturbances during one calendar year Ages: mean age 14 years (10–19 years), gender: not specified, setting: University-based secondary care specialist centre in Sweden		
Key outcomes	Framework for performing a cost analysis developed Adoption of “new” imaging method resulted in an incremental cost per examination of €46.58 (cost per examination for the new method = 128.38€ and for the conventional method = €81.80		
Study strengths	Novel framework for cost analysis of diagnostic methods Input of health economist to research team Consecutive patients		
Study weaknesses	No assessment of outcomes for patients Based on single clinic: specific costs not generalisable		

Clinical context(s)	Developmental disorders	Purpose of imaging	Diagnosis and treatment plan related to teeth next to alveolar clefts and imaging of the cleft itself
Wriedt et al. (2017)			
Diagnostic efficacy level	Level 3: Diagnostic thinking efficacy and Level 4: Therapeutic efficacy^a^		
Imaging (index tests)	1. CBCT: Accuitomo, Morita, Japan; FoV 40 × 40 mm; Voxel size: not specified 2. Panoramic radiographs [+ Study casts] Twelve clinicians made the assessments (6 maxillofacial surgeon or orthodontic specialists and 6 PG students)		
Reference standard	Not applicable		
Patient sample description	20 patients with 22 alveolar clefts, undergoing (late primary) secondary bone grafting of the alveolar cleft(s) Ages: mean age 12.5 years (± 5.5 years) (8–32 years), gender: 4F/16M, setting: University-based secondary care specialist centre in Germany		
Key outcomes	In 74% of decisions, the cleft type was the same using CBCT and radiographic imaging All cleft borders were rated as “clearly visible” using CBCT, but over half were assessed as “unclear” on radiography Decisions on “clearly defined root” and not clearly defined root” were the same using the two imaging methods in about half of cases The majority of treatment proposals “alignment possible” or “not possible” were unchanged when using CBCT For the lateral incisor, the given proposals differed by up to 43.9% At long-term review after treatment, 65.9% to 92% of the proposals concerning the alignment of teeth were correct using radiographs, and 68.2% to 94.7% of the proposals were correct using CBCT A small-volume CBCT may be justified only as supplement to a routine panoramic X-ray in selected special cases		
Study strengths	Consecutive patients Twelve examiners Inclusion of study casts, not only imaging		
Study weaknesses	Retrospective design No intraoral occlusal radiograph, only panoramic Combining observations and decisions of assessors for data analysis No intra-observer repeatability assessment		

Clinical context(s)	Pathological conditions	Purpose of imaging	Detection of resorption in association with unerupted teeth
Mak (2015)			
Diagnostic efficacy level	Level 2: Diagnostic accuracy		
Imaging (index tests)	1: CBCT [iCAT Next Generation. FoV varied; voxel size varied] 2: At least two conventional radiographs (intraoral or panoramic radiographs Nine residents, either paediatric dentistry or dental and maxillofacial radiology, served as observers		
Reference standard	“Silver standard”: opinion of one Dental Radiologist using CBCT and radiographic images		
Patient sample description	34 paediatric patients with impacted teeth. Mainly supernumerary teeth and mainly in the anterior maxilla Ages: mean 11.7 years (± 2.3 years); age range not given; maximum permissible age 18 years; gender: 18F/16M, setting: University-based secondary care specialist centre for paediatric dentistry		
Key outcomes	No significant differences in diagnostic accuracy between imaging. Using conventional radiographs gave similar specificity to when CBCT was used. Trend to higher sensitivity using CBCT CBCT: Mean Se = 47%; Mean Sp = 85% Radiographs: Mean Se = 73%; Mean Sp = 87% Resorption prevalence = 15%		
Study strengths	Nine observers, with assessment of intra-observer reliability		
Study weaknesses	Small sample size and low resorption prevalence Retrospective study Potential selection bias: convenience sample of patients who had CBCT and radiographs. Possible inclusion of more difficult cases Use of expert-based reference standard, which was based on the viewing of the index test images No standardised CBCT imaging format No standardised conventional radiograph combination		

Clinical context(s)	Pathological conditions	Purpose of imaging	Diagnosis of root resorption in permanent incisors in relation to canine impactions.
Jawad et al. (2016)			
Diagnostic efficacy level	Level 3: Diagnostic thinking efficacy		
Imaging (index tests)	CBCT: “The majority of the CBCT images were taken with an OP300 machine” with “small volume” Conventional radiography varied between patients but most only had a panoramic radiograph		
Reference standard	Not applicable		
Patient sample description	35 patient cases, over a 1-year period, in which CBCT imaging was taken to assess root resorption associated with impacted canines. 42 canines in sample, 40 in maxilla and 2 in the mandible Ages: not specified, gender: not specified, setting: University-based secondary care specialist centre in the UK		
Key outcomes	Root resorption observed on 63% of cases using CBCT and 19% of cases using radiographs Of 14 cases judged not to be resorbed on radiographs, 5 had root resorption on viewing CBCT		
Study strengths	Assessment of intra-observer repeatability made		
Study weaknesses	Retrospective study Potential selection bias (inclusion of patients who had been a priori chosen for CBCT) Lack of detail on the patient sample Variable conventional imaging Lack of detail on conduct of index tests		

Clinical context(s)	Pathological conditions	Purpose of imaging	As an aid to treatment planning for external cervical resorption (ECR)
Goodell et al. (2018)			
Diagnostic efficacy level	Level 4: therapeutic efficacy		
Imaging (index tests)	CBCT [3D Accuitomo 170; FoV = 40 × 40 mm; voxel size 0.08 mm] Intraoral radiographs (digital, CCD sensor); unclear how many and which type Six examiners (2 specialist endodontists, 2 senior endodontic residents, 2 junior endodontic residents)		
Reference standard	Not applicable		
Patient sample description	25 patients with 30 teeth referred for management of ECR and who had also undergone a CBCT examination. Ten “control” teeth of unspecified origin. Ages: not specified, gender: not specified, setting: specialist centre for endodontics in a United States Army facility		
Key outcomes	All 30 ECR cases were identified using CBCT imaging and 29 using periapical radiography Inter-rater agreement higher for CBCT Individual treatment plans changed in 56.7% of cases using CBCT Consensus decisions on dichotomised treatment plan (“repair” versus “no repair”) changed in six out of the 30 cases (20%)		
Study strengths	Clinical scenario provided to observers Specified “wash-out” time period between viewing radiographs and CBCT		
Study weaknesses	Retrospective design Risk of selection bias “Composite” consensus scores presented from examiners, for some aspects, but method of consensus unclear Presentation of diagnostic accuracy results in absence of any reference standard being specified (*results not presented here as this failed a review inclusion criterion*)		

Clinical context(s)	Other uses	Purpose of imaging	Forensic identification by recording teeth present and absent, dental restorations (extent and material), impacted teeth, any pathosis
Murphy et al. (2012)			
Diagnostic efficacy level	Level 2: diagnostic accuracy		
Imaging (index tests)	CBCT [iCAT Classic. FoV varied; voxel size varied] One forensic odontologist performed assessments, including repeated assessment for intra-observer reliability. A second person assessed a small sub-sample for inter-observer reliability.		
Reference standard	Panoramic radiograph [Sirona Orthophos CD]		
Patient sample description	30 patients who had both panoramic radiographs and CBCT examinations, consisting of 10 in each of three age cohorts (data for the ≤ 17 years cohort only considered here) Ages: not specified other than which age cohort (≤ 17 years), Gender: not specified, Setting: University-based secondary care facility in the UK		
Key outcomes	Information could be collected accurately and reliably using CBCT, compared with using panoramic radiographs CBCT: Se = 83.3% (95% CI 78.3–88.3); Sp = 100%; PPV = 100%; NPV = 99.5% (93.5–100)		
Study strengths	Clear statistical presentation Assessment of inter- and intra-observer reliability		
Study weaknesses	Retrospective study Small sample size Possible selection bias Low prevalence of positive findings to record Variable time gap between the index tests Use of a panoramic radiograph as the reference standard. May be justified as it is an existing clinical standard, but not an adequate diagnostic “truth” for diagnostic accuracy Single observer provided the main data		

*FoV* field of view of CBCT, *M* male, *F* female, *Se* sensitivity, *Sp* specificity, *Acc* accuracy, *PPV* positive predictive value, *NPV* negative predictive value, *PG* postgraduate, *sd* standard deviation

^a^Wriedt et al. (2017) presented results of actual treatment which might best be described as “prognostic efficacy”, but which are included here as an aspect of therapeutic efficacy

Case series presenting fewer than five cases are listed in Online Resource 4, with their specific clinical context. The overwhelming proportion fell into the dental anomalies category; none was in the caries category and only one in the acute dental infections category. Twenty-four case series presenting five or more patient subjects were considered in greater detail. Three publications were allocated to the guidelines category (Noffke et al. 2011; American Academy of Pediatric Dentistry 2012; Law et al. 2014), although the last of these was of a very general nature and not solely directed at CBCT.

### Supplementary information

The results of the two supplementary searches for information relevant to the review are described in Online Resources 1 and 2, respectively.

### Risk of bias within systematic reviews and diagnostic efficacy studies

Assessments of risk of bias of the 14 diagnostic efficacy studies are given in Online Resource 5. For the diagnostic accuracy category, the four studies (Murphy et al. 2012; Ziegler and Klimowicz 2013; Sansare et al. 2014; Mak 2015) fell into different clinical contexts and none was rated uniformly as free of risk of bias or without concerns about applicability. The results of quality assessment of diagnostic thinking efficacy and therapeutic efficacy studies are presented using a visual analogue scale, following a previous review method (Horner and Shelley 2016). No study was assessed at the highest level of quality, but two (Katheria et al. 2010; Jawad et al. 2016) were assessed below the mid-point of the scale for quality. The two societal efficacy publications (Christell et al. 2012a, b) were both cost analyses, a design that lacks any measurement of patient outcomes. Because of this, the Consensus Health Economic Criteria (CHEC) list (Evers et al. 2005) could not be used and the proforma used by Christell et al. (2014), based on Drummond et al. (2005), was substituted. This method does not translate to a numerical or categorical descriptor of risk of bias, but the results suggested that Christell et al. (2012b) had a low risk of bias, while Christell et al. (2012a) was judged slightly less favourably because no comparator imaging was included.

### Results according to clinical context

A comprehensive description of the findings of the review and the supplementary searches according to the clinical contexts is provided in Online Resource 6 and only a summary is provided here. Overall, there was very little evidence available specific to the paediatric age group; so, the evidence from the supplementary search for systematic reviews became important.

#### Caries

The evidence relating to CBCT and caries diagnosis relied predominantly on ex vivo research and most studies showed little difference in diagnostic accuracy when CBCT imaging was used compared with intraoral radiography (Abogazalah and Ando 2017). Ex vivo*/*in vitro imaging might result in better quality images than those obtained clinically, while artefact from adjacent high attenuation restorations is usually absent. An ex vivo study reported that that cavitation of proximal lesions can be identified more accurately when using CBCT than when using bitewing radiographs (Wenzel et al. 2013). The same group followed this up by a clinical diagnostic accuracy study, included in our review, which confirmed the ex vivo findings and concluded that cavitated caries should be reported on scans taken for other purposes (Sansare et al. 2014). There was no research evidence at the higher levels of diagnostic efficacy. Existing guidelines (Online Resource 1) provided a unanimous view against using CBCT as a standard tool for caries diagnosis.

#### Acute dental infections

There was no diagnostic efficacy evidence to suggest that acute dental infection is an indication for CBCT and no relevant guidelines were found. There was evidence, from systematic reviews of ex vivo studies (Online Resources 2 and 6), that using CBCT can give a higher diagnostic accuracy efficacy than conventional radiography for mechanically or chemically prepared periapical bone cavities (Kruse et al. 2015; Leonardi Dutra et al. 2016; Aminoshariae et al. 2018). Evidence from observational studies of patients suggests that using CBCT results in greater numbers of periapical inflammatory lesions being identified than when periapical radiography is used. Although there is a risk that false-positive diagnoses are partly responsible for this, it seems likely that true diagnostic yield from CBCT is higher than from radiographs. Several guideline publications suggest that CBCT might be used as an aid to diagnosis of periapical pathosis when conventional radiography reveals nothing but there are contradictory clinical signs and/or symptoms (Online Resource1).

#### Dental trauma

The evidence from systematic reviews of diagnostic accuracy studies predominantly performed ex vivo (Online resources 2 and 6) is that, for non-endodontically treated teeth, CBCT can lead to very high diagnostic accuracies for root fracture. Furthermore, these levels of accuracy are higher than when using periapical radiographs (Corbella et al. 2014; Hidalgo Rivas 2014; Long et al. 2014; Chang et al. 2016; Ma et al. 2016; Talwar et al. 2016; Salineiro et al. 2017). The fact that ex vivo studies lack the impact of patient movement and that the systematic reviews identified risks of bias in a large proportion of these must be recognised. For endodontically treated teeth, most of the evidence suggested that diagnostic accuracy using CBCT for detecting root fracture is lower and that false-positive diagnoses may occur. No solely paediatric studies were available and none on trauma to teeth from the primary dentition. The diagnostic thinking efficacy study by Bornstein et al. (2009), included in our review, found that fracture location on the palatal surface of the root was more coronally placed on CBCT than on radiographs. In particular, a cervical fracture was more commonly seen on CBCT, potentially influencing management. This paper was cited in a review publication by May et al. (2013) who devised a pathway for selection of CBCT that is potentially useful, but which needs further research on its impact.

#### Dental anomalies

The evidence in this context dealt with localisation of unerupted and impacted teeth, mainly permanent maxillary canines. Although one diagnostic accuracy study was identified, which reported high accuracy for tooth localisation (Ziegler and Klimowicz 2013), it was of low quality. The diagnostic thinking studies (Haney et al. 2010, Katheria et al. 2010, Alqerban et al. 2011; Botticelli et al. 2011) each reported that using CBCT led to a change in diagnosis of tooth position in a substantial minority of cases, although none of the studies was of the highest quality. Studies which looked at changes in treatment planning using CBCT found these in a proportion of cases, with increased confidence of clinicians. It seems likely that these findings would be true for any unerupted tooth requiring treatment. There was no evidence that patient outcomes are changed, but there was an increase in financial costs when using CBCT (Christell et al. 2012b). There was little diagnostic efficacy evidence for other dental anomalies apart from case studies. These included reports that CBCT was useful to image morphological anomalies of teeth, particularly in the context of planning endodontic treatment, notably for dens invaginatus anomaly, fusion and gemination.

#### Developmental disorders

With regard to developmental disorders, the publications identified by the current review were dominated by CBCT imaging of cleft lip and palate (CLP) patients. The evidence suggested that CBCT scanning prior to the procedure of bone grafting is appropriate because it permits a volumetric assessment of the defect (see Online Resource 6). It has advantages over CT in terms of radiation dose. It might be useful in imaging the teeth around a cleft, but the evidence that this changed treatment plans or prognosis was lacking in the study included in our review (Wriedt et al. 2017). Management of clefts is not a specific role of paediatric dentists, although they may be part of an interdisciplinary team caring for a patient. Apart from CLP patients, the review found a role for CBCT in production of three-dimensional datasets of the facial skeleton. There was very little evidence about the value of CBCT in specific craniofacial syndromes apart from some case studies.

#### Pathological conditions

There was a complete lack of paediatric evidence for using CBCT in periodontal diseases. From the evidence derived from adult clinical studies, CBCT would only be indicated for exceptional cases requiring complex management, for example in regenerative periodontal surgery (Kim and Bassir 2017; Woelber et al. 2018); in the paediatric context, this would be extremely rare.

The evidence regarding the diagnostic value of CBCT for resorption of teeth was weighted towards that associated with unerupted maxillary canine teeth. It is probably the most common paediatric use of CBCT and may be relevant to paediatric dentists working with their orthodontic colleagues. There is a reliance on ex vivo*/*in vitro studies of diagnostic accuracy. A review of such studies by Yi et al. (2017) found higher sensitivity but equivalent specificity for resorption detection using CBCT compared with intraoral radiography. Artificial lesions, made with a bur or application of acid, are not the same as in vivo resorption. Nonetheless, on balance, it seems reasonable to suggest that a cross-sectional imaging technique, with sufficient image quality, will out-perform a two-dimensional technique for detecting resorptions of teeth, particularly on buccal and lingual surfaces. Studies on diagnostic thinking efficacy reported changes in diagnosis in a proportion of cases when using CBCT (Haney et al. 2010; Katheria et al. 2010; Alqerban et al. 2011; Botticelli et al. 2011; Jawad et al. 2016). Some of the clinical studies cited above did not use intraoral radiographs as comparator imaging, only panoramic radiography, which might under-estimate the value of radiographs compared with CBCT. External cervical resorption is a different entity radiologically to resorption associated with tooth impactions and is impossible to model accurately in ex vivo studies. Goodell et al. (2018), using clinical material, reported that using CBCT changed treatment plans in over half of cases.

The evidence for diagnostic efficacy of CBCT for cysts, benign tumours and other benign conditions was very limited and case-based.

#### Other uses

The application of CBCT for other uses is described in Online Resource 6. One diagnostic accuracy study was included in the review dealing with forensic identification (Murphy et al. 2012), although it was essentially making a comparison of findings with those on panoramic radiographs and has limited relevance to paediatric dental practice. In view of its particular relevance to the paediatric age group, CBCT as part of surgical planning for autotransplantation of teeth is noted, specifically by allowing a three-dimensional model of the tooth to be manufactured and used to prepare the transplant site, along with production of surgical guides (Shahbazian et al. (2013).

## Discussion

The commission for this review was challenging in trying to identify the role of a diagnostic imaging technique in six clinical situations. Each of these six situations, except perhaps “caries”, was composed of several or many different contexts, for example, “pathological conditions” and “dental anomalies”. Furthermore, the diagnostic efficacy of CBCT in each context could be quite different; for example, while using CBCT might improve diagnostic accuracy for root fracture compared with a radiograph, it might not do so for a luxation injury. Thus, the review was ambitious and would better have been planned as a series of separate systematic reviews.

The decision to include only in vivo studies and those carried out in the paediatric age group, along with exclusion of orthodontic research, fitted the remit given to us. It was a strategy to make the task manageable but inevitably limited the literature. The review found little evidence relating to CBCT specific to the paediatric age group and the inclusion criteria had to be relaxed to allow any diagnostic efficacy studies to be included at all. An important finding was the absence of any research at the patient outcome efficacy level. Because of this, the findings of the supplementary search for systematic reviews based on ex vivo research and those including adult data assumed importance as a source of information.

The purpose of this review was to assist in developing guidelines on indications and contra-indications for using CBCT in paediatric dentistry. Existing guidelines on the use of CBCT are numerous and it seemed useful to provide guideline statements from these publications (Online Resource 1). The quality of guideline publications was frequently low when appraised using the AGREE II instrument, with many lacking evidence of adequate methodology (Horner et al. 2015). In the current review, no attempt was made to appraise formally those guidelines produced subsequent to that review.

It is important to reiterate some overriding factors when considering a CBCT examination of a paediatric patient. The “Basic Principles” of using CBCT (Horner et al. 2009; European Commission 2012) apply in all situations, particularly that which says *“*CBCT should only be used when the question for which imaging is required cannot be answered adequately by lower dose conventional (traditional) radiography”. Second, a careful assessment is needed to confirm the expectation that the patient can cooperate for the examination, in particular remaining motionless for a prolonged period. Previous experience from other X-ray examinations should assist. If a child has moved in the past when taking a panoramic radiograph, for example, it does not give assurance that CBCT examination will be successful. Movement of patients undergoing CBCT, producing identifiable image artefacts, is more common in paediatric patients (Donaldson et al. 2013; Nardi et al. 2015; Spin-Neto et al. 2015). Indeed, in one study, the risk of movement was 11 times higher in the 15 years or less age group than in the 31 years or older age group (Spin-Neto et al. 2015). Movement affects image quality more if it is repeated, prolonged or multiplanar during the scan acquisition (Spin-Neto et al. 2016). For diagnostic applications requiring fine detail, such as fracture diagnosis, this might be a significant problem and underlines why reliance on ex vivo*/*in vitro studies is likely to over-estimate diagnostic efficacy of CBCT. A further aspect of this is that high-resolution settings on CBCT equipment which use longer exposure times might increase the chance of movement and a paradoxical loss of image quality when it is most needed (Nardi et al. 2015).

In concluding, a few cautionary points must be made. First, there is ample evidence that the technical efficacy (Table 1) of different CBCT equipment varies. Most of the research evidence is based on studies using “high-end” expensive equipment which usually provides high-quality images. Some CBCT equipment gives inferior image quality and may never have been used in diagnostic research; so, the evidence for diagnostic efficacy for one piece of equipment may not apply to another. Second, the post-acquisition adjustment of the images makes a difference to diagnostic value, yet is usually a subjective judgement. Clinicians may or may not adjust the brightness and contrast of scans when evaluating them, but CBCT allows multiple image processing actions to be made which can change the diagnostic value. Third, for a single piece of equipment, achievable image quality is directly related to the X-ray dose and the exposure settings can impact on diagnostic accuracy; exposures should, therefore, be “ALADAIP” (As Low as Diagnostically Acceptable being Indication-oriented and Patient-specific) (Oenning et al. 2018). Fourth, and very importantly, “diagnostic accuracy” does not belong to a piece of X-ray equipment, yet too many publications use phrases such as “the diagnostic accuracy of CBCT is…”. Diagnostic accuracy (and the two subsequent levels of Fryback and Thornbury’s hierarchy) belongs to the person who performs the evaluation of the images. This value varies from individual to individual and within any single individual at different times. Finally, every patient is unique. The process of justification of CBCT examinations should not be reduced to a simple “…is indicated” or “…is contra-indicated” for a particular clinical context; instead, an individual approach is needed when choosing if CBCT is justified or not.

## Conclusions

The review found almost no in vivo study evidence specific to paediatric patients about diagnostic efficacy when using CBCT. A broader review of literature based on systematic reviews of diagnostic efficacy including ex vivo research and not specific to the paediatric age group provided some evidence of relevance.

- CBCT is not indicated for caries diagnosis. Existing scans taken for other reasons that include the teeth should be checked for caries, taking care to be aware of the risk of false-positive diagnoses.
- CBCT might rarely be indicated in exceptional cases of acute infection, where conventional radiography does not reveal the source of that infection but when a dental or bony lesion is suspected.
- CBCT may be indicated for suspected root fracture in teeth without previous endodontic treatment when conventional radiographic examination does not provide adequate information for management.
- Apart from its use in assessing clefts in CLP patients and as an alternative to CT for producing three-dimensional datasets, there was no evidence of efficacy using CBCT in developmental disorders.
- CBCT is probably indicated for the assessment of resorption (suspected or established) when conventional radiographic examination has proved to be insufficient for management.
- CBCT is probably indicated for imaging of larger bony pathoses (cysts, benign tumours and other benign bony pathosis) to show the lesion characteristics and as an aid in surgical planning.
- Patient cooperation, particularly in the context of movement during long exposure times, is an important aspect to be considered in the justification of CBCT examinations.

## Electronic supplementary material

Below is the link to the electronic supplementary material.

Supplementary material 1 (PDF 271 kb)

Supplementary material 2 (PDF 66 kb)

Supplementary material 3 (PDF 134 kb)

Supplementary material 4 (PDF 95 kb)

Supplementary material 5 (PDF 121 kb)

Supplementary material 6 (PDF 252 kb)
