# Supplementary material for: Diagnostic efficacy of cone beam computed tomography in paediatric dentistry: a systematic review
Source: Eur Arch Paediatr Dent. 2019 Dec 19;21(4):407–26. doi: 10.1007/s40368-019-00504-x (PMC7415745; doi:10.1007/s40368-019-00504-x)
Supplement: Supplementary file 1 — Supplementary material 1 (PDF 271 kb) [file 40368_2019_504_MOESM1_ESM.pdf]

ONLINE RESOURCE 1: SUPPLEMENTARY SEARCH TO IDENTIFY EXISTING GUIDELINES ON CBCT.

Methods

While some existing guidelines on the clinical use of CBCT might have been picked up in the main review search strategy, we also performed an update of the search used in a previous review by Horner et al. (2015). MEDLINE Ovid was searched on 8 October 2018, with a start date of 01.06.2014 and ending 08.10.2018.

1	guideline$.mp.
2	position statement.mp.
3	position paper.mp.
4	clinical recommendation$.mp.
5	or/1-4
6	cone beam computed tomography.mp.
7	volumetric radiography.mp.
8	volumetric tomography.mp.
9	digital volumetric tomography.mp.
10	digital volume tomography.mp.
11	cone-beam.mp. or cone-beam computed tomography/
12	(volume ct or volumetric ct).mp.
13	(volume computed tomography or volumetric computed tomography).mp.
14	cbct.mp.
15	or/6-14
16	(dental or dentistry).mp.
17	exp dentistry/
18	16 or 17
19	5 and 15 and 18

The publications identified were screened by one reviewer to identify those which were specific to the paediatric age group or which contained information that, while not specifying the age group of interest to this review, were still considered to be relevant to it. Guideline statements specific to the clinical contexts identified in the commission for the main review were collected and assembled in tabular form.

Results

Twelve guideline publications from in the original review by Horner et al (2015) were judged to be relevant to the paediatric age group and the remit of current review. After screening, the supplementary guideline search found five more guideline documents. One of these was an update of a position paper included in the review from 2015 so that original paper was excluded, leaving 18 publications. Only two of these 18 were specifically related to paediatric use of CBCT (American Academy of Pediatric Dentistry 2017; Oenning et al 2018). The Table below lists the recommendations and guidelines made in these 18 publications that were considered to be relevant to the current review. Full reference list at end of document.

TABLE: Guideline statements considered to be relevant to the current review extracted from other publications produced by professional organisations at national and international level. The source publications were derived from a previous review in 2015 (Horner et al. 2015), updated for the purpose of the current review as supplementary material. Where guidelines from the same authors/ organisations had undergone an update since the original review, only the more recent publication is listed. Only guideline statements considered to have relevance to radiology of children and young people and/ or the clinical categories in the current review are included. Some publications have been translated by the reviewers from the original languages. Best efforts were made to translate words and phrases accurately, but no guarantee is made for perfect translation. In AWMF (2013), the term "Digital Volumetric Tomography" (DVT) was used rather than CBCT; in this Table, CBCT has been substituted for DVT for consistency. *In the case of "acute dental infections", the category was used for periapical inflammation and bony infection. Shaded boxes indicate that no guideline statement or recommendation was made.

Guideline publication	Clinical use	Guideline statements
Horner et al, 2009	General	Nil specific on children. All guideline statements were general principles and not specific to the conditions for EAPD review. Relevant guideline statements:<br>• CBCT examinations must not be carried out unless a history and clinical examination have been performed<br>• CBCT examinations must be justified for each patient to demonstrate that the benefits outweigh the risks<br>• CBCT examinations should potentially add new information to aid the patient's management<br>• CBCT should not be repeated ''routinely'' on a patient without a new risk/benefit assessment having been performed<br>• CBCT should only be used when the question for which imaging is required cannot be answered adequately by lower dose conventional (traditional) radiography<br>• Where it is likely that evaluation of soft tissues will be required as part of the patient's radiological assessment, the appropriate imaging should be conventional medical CT or MR, rather than CBCT
Haute Autorité de Santé (2009)	General	Nil specific on children, except:<br>• "The CBCT technique, insofar as the associated irradiation is less*, would be of particular interest to children and young adults"

		• "X-rays without clinical indications are never justified and inevitably lead to unnecessary exposure to ionizing radiation, especially when children are concerned."<br>• "in children and adolescents, particular attention must be paid to the choice of the least irradiating imaging technique"<br>**[than multislice CT].*
	Caries	• It is at this stage impossible to conclude on the possible clinical contribution of CBCT in the field of cariology.<br>• It is important to underline that the population concerned [with caries] is predominantly paediatric therefore, first and foremost, to be exposed to less radiation.
	Acute dental infections*	
	Dental trauma	• CBCT imaging … may be of interest in some well-selected cases: assessment of a root fracture, when the information provided by clinical examination and conventional radiology are not sufficiently contributory for diagnosis and sectional imaging is essential.
	Dental anomalies	
	Developmental anomalies	
	Pathological disorders	
	Other uses	Periodontology:<br>• CBCT imaging in periodontics may be of interest in a very small number of selected cases, such as the pre-surgical assessment for the treatment of furcation of maxillary molars, by providing information not provided by the Clinical and conventional radiology (fusion-type anatomical proximity), information which would improve in these cases the taking into load and prognosis of the tooth.<br>Endodontics:

		• CBCT imaging may be of interest in some well selected cases in endodontics: pre-surgical periapical assessment particularly in the posterior maxillary region or in the region of the mental foramen, identification and localization of an additional root canal, assessment of a root pathology, resorption and / or periapical when the information provided by clinical examination and conventional radiology are not sufficiently contributory for diagnosis and sectional imaging is essential.<br>Oral Surgery:<br>• *In vivo* studies have shown that the diagnostic quality of CBCT to specify the position of anatomical elements, for example the topography of the mandibular canal before surgical extraction of third molars, was satisfactory and superior to panoramic radiography.<br>• Studies have also highlighted the technical feasibility of the CBCT exam for evaluation of bone grafts and maxillofacial fractures.<br>Temporomandibular joint:<br>• *In vitro* studies highlight the technical feasibility of CBCT for bone tissue analysis of the TMJ. To date, however, it is difficult to conclude on the diagnostic contribution of this examination compared to other techniques, tomography and CT and clinical studies comparing these techniques are required.
Advies van de Hoge Gezondheidsraad nr. 8705 (2011)	Caries	
	Acute dental infections*	
	Dental trauma	• Suspected dental alveolar trauma which cannot be visualized via conventional two-dimensional imaging, such as root fractures, dental luxations, dental intrusions, and root resorption after dentoalveolar trauma.
	Dental anomalies	• Dental anomalies (dens-in-dente, mesiodens, supernumerary tooth, ...) where the treatment approach cannot be established without further information in three dimensions.<br>• Eruption problems with impurities of definitive, supernumerary or supplementary elements.
	Developmental anomalies	

	Pathological disorders	• Diagnostic and / or therapeutic approach to benign jaw bone tumours and cysts.
	Other uses	• Diagnosis after endodontic failure to visualize aetiology of failure and possible need for retreatment.<br>• Maxillofacial surgery (diagnostic and / or if 3D aspects require it).<br>• Preoperative planning for autografts
Noffke et al (2011)	Caries	• The use of CBCT for caries detection …. is not indicated when conventional radiography can provide sufficient information. Indeed beam-hardening artefact, common with CBCT, could well result in false positive assessments for dental caries, especially where teeth in the mouth have been restored.
	Acute dental infections*	• The use of CBCT for … periapical disease is not indicated when conventional radiography can provide sufficient information.
	Dental trauma	
	Dental anomalies	• It [CBCT] may also be useful for assessment of other impacted teeth and adjacent structures when surgery or surgical orthodontics is to be undertaken.
	Developmental anomalies	
	Pathological disorders	• Maxillofacial CBCT should not be used to replace conventional CT when soft tissue detail is required to assist in the accurate radiologic interpretation of oral and maxillofacial pathoses.
	Other uses	Periodontology:<br>• The use of CBCT for … assessment of periodontal conditions … is not indicated when conventional radiography can provide sufficient information.<br>Endodontics:<br>• High resolution CBCT is useful in endodontics for assessing failure in endodontic therapy when the cause is not readily discernible from conventional radiographs.
American Association of Endodontists; American Academy	General	• The decision to order a CBCT scan must be based on the patient's history and clinical examination, and justified on an individual basis by demonstrating that the benefits to the patient outweigh the potential risks of exposure to X-rays, especially in the case of children or young adults.

of Oral and Maxillofacial Radiology. (2011)		• CBCT should only be used when the question for which imaging is required cannot be answered adequately by lower dose conventional dental radiography or alternate imaging modalities.<br>• In general, the use of CBCT in endodontics should be limited to the assessment and treatment of complex endodontic conditions
	Caries	
	Acute dental infections*	• Diagnosis of dental periapical pathosis in patients who present with contradictory or nonspecific clinical signs and symptoms, who have poorly localized symptoms.
	Dental trauma	• Diagnosis and management of dento-alveolar trauma, especially root fractures, luxation and/or displacement of teeth, and alveolar fractures.
	Dental anomalies	
	Developmental anomalies	
	Pathological disorders	• Diagnosis of non-endodontic origin pathosis in order to determine the extent of the lesion and its effect on surrounding structures.<br>• Localization and differentiation of external from internal root resorption or invasive cervical resorption from other conditions, and the determination of appropriate treatment and prognosis.
	Other uses	Endodontics:<br>• Identification of potential accessory canals in teeth with suspected complex morphology based on conventional imaging.<br>• Identification of root canal system anomalies and determination of root curvature.<br>• associated with an untreated or previously endodontically treated tooth with no evidence of pathosis identified by conventional imaging, and in cases where anatomic superimposition of roots or areas of the maxillofacial skeleton is required to perform task-specific procedures.<br>• Intra- or post-operative assessment of endodontic treatment complications, such as overextended root canal obturation material, separated endodontic instruments, calcified canal identification, and localization of perforations.

		• Pre-surgical case planning to determine the exact location of root apex/apices and to evaluate the proximity of adjacent anatomical structures.
American Dental Association Council on Scientific Affairs, 2012	General	• "CBCT should not be performed for screening purposes. Additional considerations should be weighed prior to the exposure of children and adolescents".<br>• CBCT "should be considered as an adjunct to standard oral imaging modalities. CBCT may supplement or replace conventional (two dimensional or panoramic) dental radiography for the diagnosis, monitoring and treatment of oral disease or the management of oral conditions when, in the clinician's decision-making process, he or she determines that oral anatomical structures of interest may not be captured adequately by means of conventional radiography."
	Caries	
	Acute dental infections*	
	Dental trauma	
	Dental anomalies	
	Developmental anomalies	
	Pathological disorders	
	Other uses	
European Commission (2012)	General	• Reproduction of the Horner et al (2009) basic principles.<br>• Multiple specific guidelines. Those considered to be relevant to children listed below under the clinical categories.
	Caries	• CBCT is not indicated as a method of caries detection and diagnosis.
	Acute dental infections*	• CBCT is not indicated as a standard method for identification of periapical pathosis.

		• Limited volume, high resolution CBCT may be indicated for periapical assessment, in selected cases, when conventional radiographs give a negative finding when there are contradictory positive clinical signs and symptoms.
	Dental trauma	• Limited volume, high resolution CBCT is indicated in the assessment of dental trauma (suspected root fracture) in selected cases, where conventional intraoral radiographs provide inadequate information for treatment planning.
	Dental anomalies	• For the localised assessment of an impacted tooth (including consideration of resorption of an adjacent tooth) where the current imaging method of choice is multislice CT, CBCT may be preferred because of reduced radiation dose.<br>• CBCT may be indicated for the localised assessment of an impacted tooth (including consideration of resorption of an adjacent tooth) where the current imaging method of choice is conventional dental radiography and when the information cannot be obtained adequately by lower dose conventional (traditional) radiography.
	Developmental anomalies	• Where the current imaging method of choice for the assessment of cleft palate is MSCT, CBCT may be preferred if radiation dose is lower. The smallest volume size compatible with the situation should be selected because of reduced radiation dose.<br>• For complex cases of skeletal abnormality, particularly those requiring combined orthodontic/surgical management, large volume CBCT may be justified in planning the definitive procedure, particularly where MSCT is the current imaging method of choice.
	Pathological disorders	• Limited volume, high resolution CBCT may be indicated in selected cases of suspected, or established, inflammatory root resorption or internal resorption, where three-dimensional information is likely to alter the management or prognosis of the tooth.
	Other uses	Periodontology:<br>• CBCT is not indicated as a routine method of imaging periodontal bone support.<br>• Limited volume, high resolution CBCT may be indicated in selected cases of infra-bony defects and furcation lesions, where clinical and conventional radiographic examinations do not provide the information needed for management.

		Endodontics:<br>• CBCT is not indicated as a standard method for demonstration of root canal anatomy.<br>• Limited volume, high resolution CBCT may be indicated, for selected cases where conventional intraoral radiographs provide information on root canal anatomy which is equivocal or inadequate for planning treatment, most probably in multi-rooted teeth.<br>• Limited volume, high resolution CBCT may be indicated for selected cases when planning surgical endodontic procedures. The decision should be based upon potential complicating factors, such as the proximity of important anatomical structures.<br>• Limited volume, high resolution CBCT may be justifiable for selected cases, where endodontic treatment is complicated by concurrent factors, such as resorption lesions, combined periodontal/endodontic lesions, perforations and atypical pulp anatomy.<br>Oral Surgery:<br>• CBCT may be indicated for pre-surgical assessment of an unerupted tooth in selected cases where conventional radiographs fail to provide the information required.<br>• Where it is likely that evaluation of soft tissues will be required as part of the patient’s radiological assessment, the appropriate initial imaging should be MSCT or MR, rather than CBCT.<br>• For maxillofacial fracture assessment, where cross-sectional imaging is judged to be necessary, CBCT may be indicated as an alternative imaging modality to MSCT where radiation dose is shown to be lower and soft tissue detail is not required.<br>• CBCT is indicated where bone information is required, in orthognathic surgery planning, for obtaining three-dimensional datasets of the craniofacial skeleton.<br>• Where the existing imaging modality for examination of the TMJ is MSCT, CBCT is indicated as an alternative where radiation dose is shown to be lower.<br>Temporomandibular joint:<br>• Where the existing imaging modality for examination of the TMJ is MSCT, CBCT is indicated as an alternative where radiation dose is shown to be lower

Diangelis et al. (2012)	General:	• Related to management of traumatic dental injuries: fractures and luxations of permanent teeth
	Caries	
	Acute dental infections*	
	Dental trauma	• Emerging imaging modalities such as cone-beam computerized tomography (CBCT) provide enhanced visualization of traumatic dental injuries, particularly root fractures and lateral luxations, monitoring of healing, and complications. Availability is limited, and its use not currently considered routine; however, specific information is available in the scientific literature.
	Dental anomalies	
	Developmental anomalies	
	Pathological disorders	
	Other uses	
American Academy of Oral and Maxillofacial Radiology (2013)	General	• Related to orthodontic practice. The following were extracted as being potentially relevant to paediatric dentistry and are taken from the "pre-treatment" category as situations in which CBCT is "likely indicated" (Level I).
	Caries	
	Acute dental infections*	• CBCT may be indicated for imaging in a suspected diagnosis of osteomyelitis in the jaw
	Dental trauma	
	Dental anomalies	• "Dental structure abnormalities": These comprise assessments of variations in tooth morphology, hypodontia, retained primary teeth, supernumeraries/ gemination/ fusion, root abnormalities.<br>• "Anomalies in dental position". These include dental impactions, presence of unerupted and impacted supernumeraries, locations of molars in relation to the inferior alveolar canals, anomalies in eruption sequences, and ectopic eruptions (including teeth in clefts).

	Developmental anomalies	
	Pathological disorders	• "Dental structure abnormalities": external and internal resorption.
	Other uses	
Arbeitsgemeinschaft der Wissenschaftlichen Medizinischen (AWMF, 2013)	General	• "for the application of CBCT in children and adolescents, the indication must be particularly strict."
	Caries	• CBCT for caries diagnosis is not indicated.
	Acute dental infections*	• Small-volume high-resolution CBCT can, in individual cases, be indicated for periapical examination when clinical signs and symptoms are inconsistent with two-dimensional radiographic findings.<br>• For diagnostic imaging of the suspected cases of osteomyelitis in the jaw, CBCT may be indicated.
	Dental trauma	• CBCT can be used in the evaluation of dental trauma, especially when two-dimensional radiographic images are insufficient to provide information for treatment planning and prognosis.<br>• Small-volume high-resolution CBCT can be used to detect root fractures when clinical signs and symptoms are inconsistent with two-dimensional radiographic findings.
	Dental anomalies	• The most common abnormalities of the dentition (hypodontia) can be assessed radiologically using two-dimensional radiographs with sufficient accuracy. Cases of atypical findings such as supernumerary teeth and mesiodens, displaced or impacted teeth, hyperplasia and dysplasia of dental structures could, in selected cases, undergo a CBCT scan if knowledge of the exact topographical relationship is necessary for further treatment and / or an operative intervention seems necessary.
	Developmental anomalies	• In complex malformations of the jaw and facial skull, including cleft palate, a CBCT scan can be indicated for diagnosis and therapy planning.

	Pathological disorders	• Small-volume high-resolution CBCT can be used to evaluate internal and external root resorptions when the two-dimensional imaging provides no or insufficient information for treatment planning and to determine prognosis.<br>• CBCT may be indicated to image major pathological changes in the jawbone e.g. large odontogenic cysts, odontogenic tumours or primary bone tumours, if knowledge of the position, extent and relationship to neighbouring structures is important for further management.
	Other uses	Periodontology:<br>• CBCT should not be used for routine periodontal diagnosis.<br>• Small-volume high-resolution CBCT images can be used in selected cases where clinical and two-dimensional radiographic findings are insufficient to make a therapeutic decision.<br><br>Endodontics:<br>• Small-volume high-resolution CBCT may be indicated if perforations are suspected.<br>• Small-volume high-resolution CBCT can be indicated in individual cases when endodontic therapy is made difficult by certain concomitant circumstances, such as complex anatomy of the root canal system.<br>• Small-volume high-resolution CBCT can be indicated, in selected cases, for planning of endodontic-surgical treatments, especially if aggravating factors such as the risk of anatomical neighbouring structures are present.<br>• Small-volume high-resolution CBCT can be indicated for localisation of fractured root canal instruments when two-dimensional imaging does not provide an unequivocal diagnosis.<br>Oral Surgery:<br>• Three-dimensional imaging by means CBCT is not required if two-dimensional X-ray diagnosis reveals no evidence of a particular risk situation.<br>• CBCT may be indicated if clinical diagnostics or two-dimensional X-ray diagnostics indicate a direct risk to structures is present and, at the same time, from the practitioner 's point of view, further spatial information is needed, either for the

		purpose of clarifying the risk to patients, intervention planning or for intraoperative orientation.<br>Temporomandibular joint:<br>• If, after clinical examination and information from other necessary paraclinical findings, predominantly bony temporomandibular joint disease is suspected, a CBCT examination may be indicated.<br>• To answer questions with an imaging need related to the articular disc or for articular soft tissue pathosis, CBCT is not indicated.<br>Localization of foreign bodies:<br>• CBCT can be used to localize radiopaque foreign bodies in the oral, maxillofacial and facial areas.
Faculty of General Dental Practice (UK) 2013	General	• Its [*i.e. CBCT*] use in the developing dentition should adhere strictly to appropriate selection criteria, such as those developed using a formal methodology on a European basis. Only in a very small number of cases will CBCT be required subsequent to abnormal findings identified in a conventional image.
	Caries	• CBCT should not be used as a routine method of caries diagnosis.
	Acute dental infections*	
	Dental trauma	• In some instances, a small field of view cone beam computer tomography (CBCT) scan may be desirable to assess the true nature of luxation injuries and/or cortical plate involvement.
	Dental anomalies	• For the assessment of an impacted tooth and possible resorption of an adjacent tooth, CBCT may be used when the information cannot be obtained adequately by lower-dose conventional radiography.
	Developmental anomalies	
	Pathological disorders	• Limited-volume, high-resolution CBCT may be indicated in selected cases of suspected, or established, external resorption or internal resorption, where three-dimensional information is likely to alter the management or prognosis of the tooth.
	Other uses	Periodontology:<br>• CBCT is not indicated as a routine method of imaging periodontal bone support.

		• Where CBCT images include the teeth, care should be taken to check for periodontal bone levels when performing a clinical evaluation (report).<br><br>Endodontics:<br>• CBCT is not indicated as a standard method for demonstration of root canal anatomy.<br>• Limited-volume, high-resolution CBCT may be indicated, for selected cases where conventional intraoral radiographs provide information on root canal anatomy which is equivocal or inadequate for planning treatment, most probably in multirooted teeth.<br>• Limited-volume, high-resolution CBCT may be indicated for selected cases when planning surgical endodontic procedures. The decision should be based upon potential complicating factors, such as the proximity of important anatomical structures.<br>• Limited-volume, high-resolution CBCT may be justifiable for selected cases, where endodontic treatment is complicated by concurrent factors, such as resorption lesions, combined periodontal/endodontic lesions, suspected perforations, treatment planning prior to periapical microsurgery and atypical root canal anatomy.
Dula et al. (2014)	General	SADMFR guidelines for oral and maxillofacial surgery, temporomandibular joint dysfunctions and disorders, and orthodontics.
	Caries	
	Acute dental infections*	
	Dental trauma	• Crown-Root Fractures: in unclear cases, the visualization of the fracture line and knowledge about its course serves to elucidate the possibilities of tooth conservation.<br>• Isolated maxillofacial trauma with a need for 3-D imaging, with no suspicion of intracerebral lesion or any other indication for soft tissue imaging (e.g. suspected lesions to major vessels)

	Dental anomalies	• For unerupted impacted/ retained molars, canines, premolars, a radiographic evaluation with CBCT should be performed only when information about pathological changes or for surgical removal is needed that cannot be obtained from standard radiographs.<br>• For supernumerary teeth, in complex situations where precise information is required, particularly with regard to related pathologies, such as cystic lesions, resorption of adjacent roots or multiple retention with crowding, a CBCT is indicated prior to the removal of these teeth.
	Developmental anomalies	
	Pathological disorders	• CBCT may be indicated for a more exact localization and for a better evaluation of the position and the dimension of the cyst in relationship to other important anatomic structures.<br>• CBCT can nowadays be considered as the method of choice for examining benign tumours of the jaws.<br>• For the diagnosis of malignant bone tumours of the jaw region, MSCT must be applied instead in order to assess possible soft tissue infiltration and lymph node involvement.<br>• In all cases of replacement resorption, infection-related root resorption, invasive cervical resorption, and internal root resorption, a CBCT is indicated in order to evaluate the possibility of saving the involved tooth.
	Other uses	Endodontics:<br>• Prior to apical surgery of maxillary molars.<br>• Prior to apical surgery of mandibular molars with difficult anatomy or pathology.<br>• Prior to apical surgery (all teeth), when there are clinical signs of a periapical problem with no signs of periapical pathology in the intraoral radiograph; when there are sensitive anatomical structures near the apex; when there is “difficult pathology”.<br><br>Temporomandibular joint:

		• With the few exceptions, additional information obtained by CBCT images does not result in the modification of therapeutic decisions in patients with TMJ problems.<br>• CBCT is not indicated for TMJ-related routine diagnosis in daily practice.<br><br>Foreign body:<br>• If radiopaque, CBCT is suggested for identification of exact location.
European Society of Endodontology (2014)	General	• A request for a CBCT scan should only be considered if the additional information from reconstructed three-dimensional images will potentially aid formulating a diagnosis and/or enhance the management of a tooth with an endodontic problem(s).
	Caries	
	Acute dental infections*	• Diagnosis of radiographic signs of periapical pathosis when there are contradictory (nonspecific) signs and/or symptoms.
	Dental trauma	• Assessment and/or management of complex dento-alveolar trauma, such as severe luxation injuries, suspected fracture of the overlying alveolar complex and horizontal root fractures, which may not be readily evaluated with conventional radiographic views
	Dental anomalies	
	Developmental anomalies	
	Pathological disorders	• Confirmation of nonodontogenic causes of pathosis.<br>• Assessment and/or management of root resorption, which clinically appears to be potentially amenable to treatment.
	Other uses	Endodontics:<br>• Appreciation of extremely complex root canal systems prior to endodontic management (for example, class III & IV *dens invaginatus*)<br>• Assessment of extremely complex root canal anatomy in teeth treatment planned for nonsurgical endodontic re-treatment

		• Assessment of endodontic treatment complications (for examples, [post] perforations) for treatment planning purposes when existing conventional radiographic views have yielded insufficient information<br>• Pre-surgical assessment prior to complex periradicular surgery (for example posterior teeth).
AAE and AAOMR (2015)	General	• Specialist endodontic guidelines<br>• Intraoral radiographs should be considered the imaging modality of choice in the evaluation of the endodontic patient.<br>• Limited FOV CBCT should be considered the imaging modality of choice for diagnosis in patients who present with contradictory or non-specific clinical signs and symptoms associated with untreated or previously endodontically treated teeth.
	Caries	
	Acute dental infections*	
	Dental trauma	• Limited FOV CBCT should be considered the imaging modality of choice if clinical examination and 2D intraoral radiography are inconclusive in the detection of vertical root fracture (VRF).<br>• Limited FOV CBCT should be considered the imaging modality of choice for diagnosis and management of limited dento-alveolar trauma, root fractures, luxation, and /or displacement of teeth and localized alveolar fractures, in the absence of other maxillofacial or soft tissue injury that may require other advanced imaging modalities.
	Dental anomalies	
	Developmental anomalies	
	Pathological disorders	• Limited FOV CBCT is the imaging modality of choice in the localization and differentiation of external and internal resorptive defects and the determination of appropriate treatment and prognosis.
	Other uses	Endodontics:

		• Limited FOV CBCT should be considered the imaging modality of choice for initial treatment of teeth with the potential for extra canals and suspected complex morphology, such as mandibular anterior teeth, and maxillary and mandibular premolars and molars, and dental anomalies.<br>• If a preoperative CBCT has not been taken, limited FOV CBCT should be considered as the imaging modality of choice for intra-appointment identification and localization of calcified canals.<br>• Intraoral radiographs should be considered the imaging modality of choice for immediate postoperative imaging.<br>• Limited FOV CBCT should be the imaging modality of choice when evaluating the non-healing of previous endodontic treatment to help determine the need for further treatment, such as non-surgical, surgical or extraction.<br>• Limited FOV CBCT should be the imaging modality of choice for non-surgical re-treatment to assess endodontic treatment complications, such as overextended root canal obturation material, separated endodontic instruments, and localization of perforations.<br>• Limited FOV CBCT should be considered as the imaging modality of choice for pre-surgical treatment planning to localize root apex/apices and to evaluate the proximity to adjacent anatomical structures.
Dula et al. (2015)		SADMFR guidelines for endodontics, periodontology, reconstructive dentistry and paediatric dentistry.<br>• For paediatric dentistry, the following statement: *"The usefulness of CBCT in pediatric dentistry is therefore limited to specific diseases of children. These are generally diseases with greater importance to the overall health of the child, such as specific or severe inflammations, bone diseases, benign and malignant tumors or other very special pathological conditions."*
	Caries	• No justification can be found for the use of CBCT for caries detection.
	Acute dental infections*	
	Dental trauma	

	Dental anomalies	
	Developmental anomalies	
	Pathological disorders	High-resolution CBCT is justified in cases of suspected cervically invasive root resorption.
	Other uses	Endodontics:<br>• In teeth with atypical anatomy, which could potentially impact endodontic treatment, a CBCT scan may be considered. (e.g. *radix ento-* and *paramolaris*, *dens invaginatus*, and palatal grooves.<br>• For radiolucencies potentially mimicking apical lesions.
American Academy of Pediatric Dentistry 2012 (2017 revision)	General	• “The use of CBCT has been valuable as an adjunct diagnostic tool in assessing periapical pathosis in endodontics, oral pathology, anomalies in the developing dentition (e.g., impacted, ectopic, or supernumerary teeth), oral maxillofacial surgery (e.g., cleft palate), dental and facial trauma, and orthodontic and surgical preparation for orthognathic surgery”<br>• Intraoral imaging should be maintained as the standard diagnostic tool.<br>• “The use of CBCT should be considered when conventional radiographs are inadequate to complete diagnosis and treatment planning and the potential benefits outweigh the risk of additional radiation dose. It must not be routinely prescribed for diagnosis or screening purposes in the absence of clinical indication”
Mandelaris et al (2017)	General:	Guidelines from the American Academy of Periodontology<br>“There is insufficient evidence to support its [*i.e.CBCT*] use and implementation for routine periodontal treatment planning.”
	Caries	
	Acute dental infections*	
	Dental trauma	
	Dental anomalies	

	Developmental anomalies	
	Pathological disorders	
	Other uses	Periodontology:<br>CBCT may be useful in the management of patients with periodontitis according to the following scenarios:<br>• When an advanced furcation lesion has been detected and dental implants are being considered as an alternative treatment option<br>• When advanced bone loss has encroached on anatomic structures, such as sinus cavities or the inferior alveolar nerve<br>• When there is a questionable root fracture, root resorption, or periodontal-endodontic lesion present that could not be identified by 2D imaging and/or clinical evaluation.<br>• In the retreatment of cases that don't respond favourably to localized periodontal therapy
Oenning et al 2018 (DIMITRA project)	Caries	
	Acute dental infections*	
	Dental trauma	• Dentoalveolar trauma (ages 6-15)
	Dental anomalies	• Impacted and supplementary teeth (ages 6-18).<br>• Anomalies of tooth form (morphology, density, outline, dimension, pulp chamber size and shape) at ages 6-15.
	Developmental anomalies	• Orofacial clefts, for diagnosis of deformity, tooth development and eruption and graft planning (ages 8-11), evaluation of graft healing, jaw relationships, asymmetries and discrepancies, orthodontic approach and orthognathic surgery (ages 11-18).<br>• Diagnosis and follow-up of paediatric syndromic cases, especially those with severe craniofacial abnormalities, lesions or multiple dental anomalies and teeth impaction.

	Pathological disorders	• Benign “bone pathology” (ages 6-15).
	Other uses	• Cone-beam-CT-based surgical planning of autotransplantation

References of guideline publications on use of CBCT containing at least some recommendations of relevance to paediatric age groups.

Advies van de Hoge Gezondheidsraad nr. 8705. Dentale Cone Beam Computed Tomography. Brussel: Hoge Gezondheidsraad; 2011.

American Academy of Oral and Maxillofacial Radiology. Clinical recommendations regarding use of cone beam computed tomography in orthodontics. Oral Surg Oral Med Oral Pathol Oral Radiol. 2013;116:238–57.

American Academy of Pediatric Dentistry. Prescribing Dental Radiographs for Infants, Children, Adolescents, and Individuals with Special Health Care Needs. Pediatr Dent. 2017;39:205-07.

American Association of Endodontists; American Academy of Oral and Maxillofacial Radiology. Use of cone-beam computed tomography in endodontics Joint Position Statement of the American Association of Endodontists and the American Academy of Oral and Maxillofacial Radiology. Oral Surg Oral Med Oral Pathol Oral Radiol Endod. 2011; 111: 234-7.

American Dental Association Council on Scientific Affairs. The use of cone-beam computed tomography in dentistry: an advisory statement from the American Dental Association Council on Scientific Affairs. J Am Dent Assoc 2012; 143: 899-902.

Arbeitsgemeinschaft der Wissenschaftlichen Medizinischen Fachgesellschaften (AWMF). s2k-Leitlinie Dentale digitale Volumentomographie Version Nr. 9 vom 5 August 2013. AWMF-Register-Nummer:083–05. D¨usseldorf: AWMF; 2013. Available from: https://www.awmf.org/uploads/tx_szleitlinien/083-005l_S2k_Dentale_Volumentomographie_2013-10-abgelaufen.pdf. Accessed 5 September 2019.

Diangelis AJ, Andreasen JO, Ebeleseder KA, Kenny DJ, Trope M, Sigurdsson A, et al. International Association of Dental Traumatology guidelines for the management of traumatic dental injuries: 1. Fractures and luxations of permanent teeth. Dent Traumatol. 2012;28:2-12.

Dula K, Benic GI, Bornstein M, Dagassan-Berndt D, Filippi A, Hicklin S, Kissling-Jeger F, Luebbers HT, Sculean A, Sequeira-Byron P, Walter C, Zehnder M. SADMFR Guidelines for the Use of Cone-Beam Computed Tomography/Digital Volume Tomography. Swiss Dent J. 2015;125:945-53.

Dula K, Bornstein MM, Buser D, Dagassan-Berndt D, Ettlin DA, Filippi A, Gabioud F, Katsaros C, Krastl G, Lambrecht JT, Lauber R, Luebbers HT, Pazera P, Türp JC; SADMFR. SADMFR guidelines for the use of Cone-Beam Computed Tomography/ Digital Volume Tomography. Swiss Dent J. 2014;124:1169-83.

European Commission. Radiation protection 172. Evidence based guidelines on cone beam CT for dental and maxillofacial radiology. Luxembourg: Office for Official Publications of the European Communities; 2012. https://ec.europa.eu/energy/sites/ener/files/documents/172.pdf. Accessed 5 September 2019.

European Society of Endodontology; Patel S, Durack C, Abella F, Roig M, Shemesh H, Lambrechts P, et al. European Society of Endodontology position statement: the use of CBCT in endodontics. Int Endod J 2014;47:502–4.

Faculty of General Dental Practice (UK). Horner K, Eaton KA, eds. Selection criteria for dental radiography. 3rd edn. London, UK: Faculty of General Dental Practice (UK) Royal College of Surgeons of Surgeons of England; 2013.

Haute Autorité de Santé. Tomographie Volumique a Faisceau Conique de la Face (Cone Beam Computerized Tomography). Rapport d'évaluation Technologique. Service évaluation des actes professionnels. Saint-Denis La Plaine: Haute Autorité de Santé; 2009.

Horner K, Islam M, Flygare L, Tsiklakis K, Whaites E. Basic principles for use of dental cone beam computed tomography: consensus guidelines of the European Academy of Dental and Maxillofacial Radiology. Dentomaxillofac Radiol. 2009;38:87-95.

Mandelaris, G.A., Scheyer, E.T., Evans, M., Kim, D., McAllister, B., Nevins, M.L., Rios, H.F., and Sarment, D., American Academy of Periodontology Best Evidence Consensus Statement on Selected Oral Applications for Cone-Beam Computed Tomography. J Periodontol. 2017;88:939-45.

Noffke CE, Farman AG, Nel S, Nzima N. Guidelines for the safe use of dental and maxillofacial CBCT: a review with recommendations for South Africa. SADJ. 2011;66:262, 264-6.

Oenning AC, Jacobs R, Pauwels R, Stratis A, Hedesiu M, Salmon B; DIMITRA Research Group, http://www.dimitra.be. Cone-beam CT in paediatric dentistry: DIMITRA project position statement. Pediatr Radiol. 2018;48:308-16.

Special Committee to Revise the Joint AAE/AAOMR Position Statement on use of CBCT in Endodontics. AAE and AAOMR Joint Position Statement: Use of Cone Beam Computed Tomography in Endodontics 2015 Update. Oral Surg Oral Med Oral Pathol Oral Radiol. 2015;120:508-12.

<u>Other reference</u>:
Horner K, O'Malley L, Taylor K, Glenny AM. Guidelines for clinical use of CBCT: a review. Dentomaxillofac Radiol. 2015;44:20140225.
