# Supplementary material for: Diagnostic efficacy of cone beam computed tomography in paediatric dentistry: a systematic review
Source: Eur Arch Paediatr Dent. 2019 Dec 19;21(4):407–26. doi: 10.1007/s40368-019-00504-x (PMC7415745; doi:10.1007/s40368-019-00504-x)
Supplement: Supplementary file 2 — Supplementary material 2 (PDF 66 kb) [file 40368_2019_504_MOESM2_ESM.pdf]

ONLINE RESOURCE 2: SUPPLEMENTARY BROAD SEARCH FOR SYSTEMATIC REVIEWS ON CBCT.

Methods

In anticipation that limited numbers of systematic reviews or primary research studies on *in vivo* diagnostic efficacy of CBCT in the paediatric age group would be identified by the review, systematic reviews including *ex vivo / in vitro* studies and i*n vivo* studies of, or including, adult patients were identified as supplementary information. The Medline Ovid search strategy is shown below. The publications identified were screened by one reviewer to identify those considered to provide information relevant to the main review. Publications on implant dentistry were excluded at the screening stage, as these were considered to have no significant relevance to paediatric age groups.

TABLE 1: The supplementary Medline Ovid search strategy, linked to the SIGN systematic review filter, used for identification of systematic reviews on diagnostic efficacy of CBCT in dentistry.

1	exp "cone-beam computed tomography"/
2	("cone-beam computed tomography" or "cone-beam CAT scan$" or "cone beam CT scan$" or "cone-beam CT" or "cone-beam computer-assisted tomography" or "cone-beam computeri?ed tomography" or "cone-beam computed tomography").mp.
3	("volume CT" or "volume computed tomography" or "volumetric CT" or "volumetric computed tomography").mp
4	("digital volumetric tomography" or "digital volume tomography").mp.
5	(cbct or qcbct).mp.
6	or/1-5
7	exp dentistry/
8	exp tooth diseases/di, dg
9	(oral or dental or intra-oral or intraoral or dentist$).mp.
10	(caries or carious or (tooth adj3 decay) or (teeth adj3 decay)).mp.
11	((tooth or teeth or dental) adj5 (infect$ or diseas$ or trauma$ or injur$ or luxat$ or avuls$)).mp.
12	exp Mouth abnormalities/
13	(orthodontic$ or malocclusion or cleft$ or "open bite" or "deep bite" or ((tooth or teeth) adj crowd$) or "cross bite" or crossbite).mp.
14	or/7-13
15	6 and 14
The search was linked to the systematic review filter from the Scottish Intercollegiate Guidelines Network (SIGN) to limit a search to systematic reviews in MEDLINE (from SIGN, Search filters, online at https://www.sign.ac.uk/search-filters.html, accessed 17 October 2018):	
1	Meta-Analysis as Topic/
2	meta analy$.tw.
3	metaanaly$.tw.
4	Meta-Analysis/
5	(systematic adj (review$1 or overview$1)).tw.
6	exp Review Literature as Topic/

7	or/1-6
8	cochrane.ab.
9	embase.ab
10	(psychlit or psyclit).ab.
11	(psychinfo or psycinfo).ab.
12	(cinahl or cinhal).ab.
13	science citation index.ab.
14	bids.ab.
15	cancerlit.ab.
16	or/8-15
17	reference list$.ab.
18	bibliograph$.ab
19	hand-search$.ab.
20	relevant journals.ab.
21	manual search$.ab.
22	or/17-21
23	selection criteria.ab.
24	data extraction.ab.
25	23 or 24
26	Review/
27	25 and 26
28	Comment/
29	Letter/
30	Editorial/
31	animal/
32	human/
33	31 not (31 and 32)
34	or/28-30,33
35	7 or 16 or 22 or 27
36	35 not 34

Results

The flow of articles is shown on a PRISMA chart in Fig. 1. This search strategy identified 159 publications. One highly pertinent review (De Grauwe et al. 2018) had been published shortly after the date of the main review search and had narrowly missed inclusion there, but was captured in this supplementary search. Two other systematic reviews, known to one of the reviewers (KH), were added (Ma et al. 2016a; Nikolic-Jakoba et al. 2016). Screening of the title and abstracts reduced this to 29 publications and this was further reduced to 26 systematic reviews being included which addressed the clinical conditions specified in the current review.

Figure 1: PRISMA flow chart (Moher et al. 2009) showing the flow of publications arising from the supplementary search for systematic reviews of diagnostic efficacy using CBCT and with broad inclusion criteria, permitting *ex vivo/ in vitro* studies and *in vivo* studies of adults.

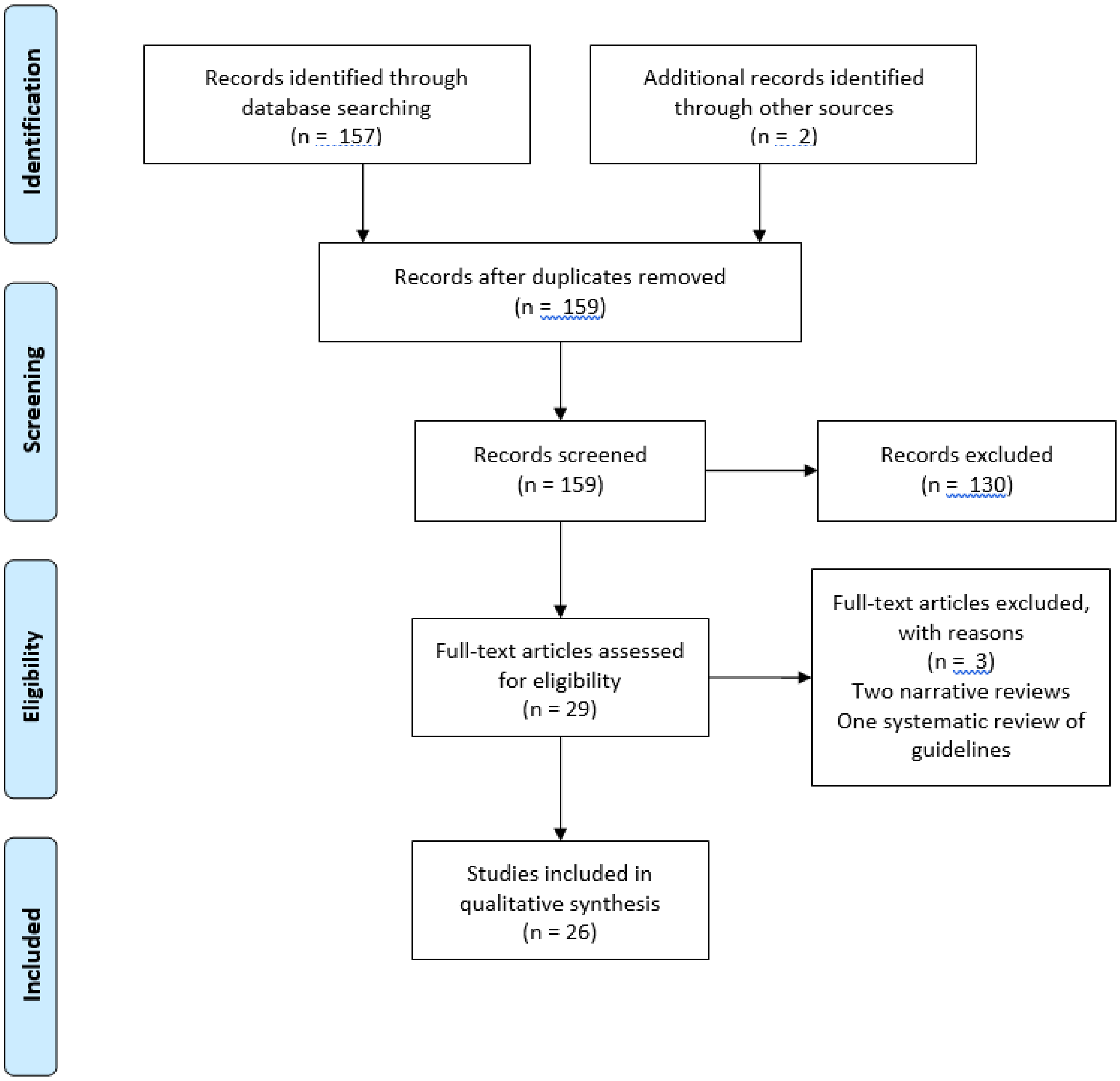

<u>References</u>: Systematic reviews on diagnostic efficacy using CBCT, identified as part of the broad supplementary search. These reviews include primary research on diagnostic efficacy using *ex vivo/ in vitro* designs and, for *in vivo* studies, adult subjects.

Abogazalah N, Ando M. Alternative methods to visual and radiographic examinations for approximal caries detection. J Oral Sci. 2017;59:315-22.

Aminoshariae A, Kulild JC, Syed A. Cone-beam Computed Tomography Compared with Intraoral Radiographic Lesions in Endodontic Outcome Studies: A Systematic Review. J Endod. 2018;44:1626-31.

Chang E, Lam E, Shah P, Azarpazhooh A. Cone-beam Computed Tomography for Detecting Vertical Root Fractures in Endodontically Treated Teeth: A Systematic Review. J Endod. 2016;42:177-85.

Choi IGG, Cortes ARG, Arita ES, Georgetti MAP. Comparison of conventional imaging techniques and CBCT for periodontal evaluation: A systematic review. Imaging Sci Dent. 2018;48:79-86.

Corbella S, Del Fabbro M, Tamse A, Rosen E, Tsesis I, Taschieri S. Cone beam computed tomography for the diagnosis of vertical root fractures: a systematic review of the literature and meta-analysis. Oral Surg Oral Med Oral Pathol Oral Radiol. 2014;118:593-602.

De Grauwe A, Ayaz I, Shujaat S, Dimitrov S, Gbadegbegnon L Van de Vannet B, Jacobs R. CBCT in orthodontics: a systematic review on justification of CBCT in a paediatric population prior to orthodontic treatment. Eur J Orthod. 2019 41:381-89.

De Vos W, Casselman J, Swennen GR. Cone-beam computerized tomography (CBCT) imaging of the oral and maxillofacial region: a systematic review of the literature. Int J Oral Maxillofac Surg. 2009;38:609-25.

Eslami E, Barkhordar H, Abramovitch K, Kim J, Masoud MI.Cone-beam computed tomography vs conventional radiography in visualization of maxillary impacted-canine localization: A systematic review of comparative studies. Am J Orthod Dentofacial Orthop. 2017;151:248-58.

Guerrero ME, Shahbazian M, Elsiena Bekkering G, Nackaerts O, Jacobs R, Horner K. The diagnostic efficacy of cone beam CT for impacted teeth and associated features: a systematic review. J Oral Rehabil. 2011;38:208-16.

Hussain AM, Packota G, Major PW, Flores-Mir C.Role of different imaging modalities in assessment of temporomandibular joint erosions and osteophytes: a systematic review. Dentomaxillofac Radiol. 2008;37:63-71.

Kim DM, Bassir SH. When Is Cone-Beam Computed Tomography Imaging Appropriate for Diagnostic Inquiry in the Management of Inflammatory Periodontitis? An American Academy of Periodontology Best Evidence Review. J Periodontol. 2017;88:978-98.

Kruse C, Spin-Neto R, Wenzel A, Kirkevang LL. Cone beam computed tomography and periapical lesions: a systematic review analysing studies on diagnostic efficacy by a hierarchical model. Int Endod J. 2015;48:815-28.

Kuijpers MA, Chiu YT, Nada RM3 Carels CE, Fudalej PS. Three-dimensional imaging methods for quantitative analysis of facial soft tissues and skeletal morphology in patients with orofacial clefts: a systematic review. PLoS One. 2014;9:e93442.

Leonardi Dutra K, Haas L, Porporatti AL, Flores-Mir C, Nascimento Santos J, Mezzomo LA, Corrêa M, De Luca Canto G. Diagnostic Accuracy of Cone-beam Computed Tomography and Conventional Radiography on Apical Periodontitis: A Systematic Review and Meta-analysis. J Endod. 2016;42:356-64.

Long H, Zhou Y, Ye N, Liao L, Jian F, Wang Y, Lai W. Diagnostic accuracy of CBCT for tooth fractures: a meta-analysis. J Dent. 2014;42:240-8.

Ma RH, Ge ZP, Li G. Detection accuracy of root fractures in cone-beam computed tomography images: a systematic review and meta-analysis. Int Endod J. 2016a;49:646-54.

Ma RH, Yin S, Li G. The detection accuracy of cone beam CT for osseous defects of the temporomandibular joint: a systematic review and meta-analysis. Sci Rep. 2016b;6:34714.

Nikolic-Jakoba N, Spin-Neto R, Wenzel A. Cone-Beam Computed Tomography for Detection of Intrabony and Furcation Defects: A Systematic Review Based on a Hierarchical Model for Diagnostic Efficacy. J Periodontol. 2016;87:630-44.

Petersson A. What you can and cannot see in TMJ imaging--an overview related to the RDC/TMD diagnostic system. J Oral Rehabil. 2010;37:771-8.

Petersson A, Axelsson S, Davidson T, Frisk F, Hakeberg M, Kvist T, Norlund A, Mejàre I, Portenier I, Sandberg H, Tranaeus S, Bergenholtz G. Radiological diagnosis of periapical bone tissue lesions in endodontics: a systematic review. Int Endod J. 2012;45:783-801.

Rosen E, Taschieri S, Del Fabbro M, Beitlitum I, Tsesis I. The Diagnostic Efficacy of Cone-beam Computed Tomography in Endodontics: A Systematic Review and Analysis by a Hierarchical Model of Efficacy. J Endod. 2015;41:1008-14.

Salineiro FCS, Kobayashi-Velasco S, Braga MM, Cavalcanti MGP. Radiographic diagnosis of root fractures: a systematic review, meta-analyses and sources of heterogeneity. Dentomaxillofac Radiol. 2017;46:20170400.

Talwar S, Utneja S, Nawal RR, Kaushik A, Srivastava D, Oberoy SS. Role of cone-beam computed tomography in diagnosis of vertical root fractures: a systematic review and meta-analysis. J Endod.2016;42:12-24.

van Vlijmen OJ, Kuijpers MA, Bergé SJ, Schols JG, Maal TJ, Breuning H, Kuijpers-Jagtman AM. Evidence supporting the use of cone-beam computed tomography in orthodontics. J Am Dent Assoc. 2012;143:241-52.

Woelber JP, Fleiner J, Rau J, Ratka-Krüger P, Hannig C. Accuracy and usefulness of cbct in periodontology: a systematic review of the literature. Int J Periodontics Restorative Dent. 2018;38:289-97.

Yi J, Sun Y, Li Y, Li C, Li X, Zhao Z. Cone-beam computed tomography versus periapical radiograph for diagnosing external root resorption: A systematic review and meta-analysis. Angle Orthod. 2017;87:328-37.
