# Supplementary material for: Diagnostic efficacy of cone beam computed tomography in paediatric dentistry: a systematic review
Source: Eur Arch Paediatr Dent. 2019 Dec 19;21(4):407–26. doi: 10.1007/s40368-019-00504-x (PMC7415745; doi:10.1007/s40368-019-00504-x)
Supplement: Supplementary file 3 — Supplementary material 3 (PDF 134 kb) [file 40368_2019_504_MOESM3_ESM.pdf]

ONLINE RESOURCE 3: LIST OF PUBLICATIONS IDENTIFIED BY THE MAIN REVIEW SEARCH STRATEGY AND INCLUDED IN THE REVIEW.

Adolphs N, Liu W, Keeve E, Hoffmeister B. Craniomaxillofacial surgery planning based on 3D models derived from Cone-Beam CT data. Comput Aided Surg. 2013;18:101-8.

Agrawal PK, Wankhade J, Warhadpande M. A Rare Case of Type III Dens Invaginatus in a Mandibular Second Premolar and Its Nonsurgical Endodontic Management by Using Cone-beam Computed Tomography: A Case Report. J Endod. 2016;42:669-72.

Ahmed F, Brooks SL, Kapila SD. Efficacy of identifying maxillofacial lesions in cone-beam computed tomographs by orthodontists and orthodontic residents with third-party software. Am J Orthod Dentofacial Orthop. 2012;141:451-9.

Allison JR, Garlington G. The Value of Cone Beam Computed Tomography in the Management of Dentigerous Cysts - A Review and Case Report. Dent Update. 2017;44:182-4, 6-8.

Almeida-Barros RQ, Abilio VM, Yamamoto AT, Melo DP, Godoy GP, Bento PM. Digital panoramic radiography versus cone beam computed tomography in the delineation of maxillomandibular tumors. Gen Dent. 2015;63:e5-e10.

Alqerban A, Jacobs R, Fieuws S, Willems G. Comparison of two cone beam computed tomographic systems versus panoramic imaging for localization of impacted maxillary canines and detection of root resorption. Eur J Orthod. 2011;33:93-102.

Al-Sehaibany FS, Marzouk HM, Salama FS. Cone Beam Computed Tomography Evaluation of Inverted Mesiodentes. J Dent Child (Chic). 2016;83:88-93.

Ambu E, Fimiani M, Vigna M, Grandini S. Use of bioactive materials and limited FOV CBCT in the treatment of a replanted permanent tooth affected by inflammatory external root resorption: a case report. Eur J Paediatr Dent. 2017;18:51-55.

American Academy of Pediatric Dentistry. Ad Hoc Committee on Pedodontic R. Guideline on prescribing dental radiographs for infants, children, adolescents, and persons with special health care needs. Pediatr Dent. 2012;34:189-91.

Aps JK. Cone beam computed tomography in paediatric dentistry: overview of recent literature. Eur Arch Paediatr Dent. 2013;14:131-40.

Araki M, Namaki S, Amemiya T, Matsumoto K, Honda K, Yonehara Y, et al. Diverse calcification patterns of ameloblastic fibro-odontoma on radiographic examination. J Oral Sci. 2016;58:533-537.

Arvonen P, Naujokaityte J, Arvonen M, Niinimaki J, Vahasalo P, Pirttiniemi P. PReS-FINAL-2246: CBCT versus orthopan tomogram detecting TMJ alteration in JIA. Pediatric Rheumatology Conference: 20th Pediatric Rheumatology European

Society, PReS Congress Ljubljana Slovenia Conference. Pediatr Rheumatol. 2013;(Suppl 2):P236.

Augello M, Rabufetti A, Ghazal G, Yurtsever H, Leiggener C. Ameloblastic fibro-odontoma in children. Clinical aspects and review of the literature. Oral and Maxillofacial Surgery Cases. 2017;3:34-41.

Bahadure RN, Thosar N, Khubchandani M. Orthodontic extrusion: diagnosis and treatment with CBCT in a pediatric patient. Gen Dent. 2013;61:e5-7.

Barghan S, Tetradis S, Nervina JM. Skeletal and soft-tissue incidental findings on cone-beam computed tomography images. Am J Orthod Dentofacial Orthop. 2013;143:888-92.

Beier US, Dumfahrt H, Widmann G, Puelacher W. Surgical resection technique of a fused supernumerary lateral incisor: A clinical report and review of the literature. Gen Dent. 2012;60:e268-73.

Bianchi A, Muyldermans L, Di Martino M, Lancellotti L, Amadori S, Sarti A, et al. Facial soft tissue esthetic predictions: validation in craniomaxillofacial surgery with cone beam computed tomography data. J Oral Maxillofac Surg. 2010;68:1471-9.

Binsaleh S. Predicting Adoption of Cone Beam Computed Tomography among Pediatric Dentists. [M.Sc thesis, University of Illinois at Chicago]. 2017;10708524:48.

Bockow R, Evans M, Chung CH. Diagnosis of anomalous teeth with cone-beam computed tomography. J Clin Orthod. 2012;46:156-8.

Boffano P, Gallesio C, Roccia F, Berrone S. Bilateral buccal bifurcation cyst. J Craniofac Surg. 2012;23:e643-5.

Borges AH, Mamede-Neto I, Volpato LE, Pedro FL, Bueno MR, Estrela C. Using cone beam computed tomography images to diagnose multiple taurodontisms. Gen Dent. 2014;62:e20-2.

Bornstein MM, Wolner-Hanssen AB, Sendi P, von Arx T. Comparison of intraoral radiography and limited cone beam computed tomography for the assessment of root-fractured permanent teeth. Dental Traumatol. 2009;25:571-7.

Botticelli S, Verna C, Cattaneo PM, Heidmann J, Melsen B. Two- versus three-dimensional imaging in subjects with unerupted maxillary canines. Eur J Orthod. 2011;33:344-9.

Brauer HU. Case report: non-syndromic multiple supernumerary teeth localized by cone beam computed tomography. Eur Arch Paediatr Dent. 2010;11:41-3.

Brauer HU, Diaz C, Manegold-Brauer G. Radiographic assessment of a keratocystic odontogenic tumour using cone-beam computed tomography. Eur Arch Paediatr Dent. 2013;14:173-7.

Burak DS, Deniz S, Sacide D, Numan D. A case report: Bilateral coronoid hyperplasia. Surg Radiol Anat. 2018;40 (1 Supplement 1):S45-S6.

Canoglu E, Canoglu H, Aktas A, Cehreli ZC. Isolated bilateral macrodontia of mandibular second premolars: A case report. Eur J Dent. 2012;6:330-4.

Cantekin K, Sekerci AE, Miloglu O, Buyuk SK. Identification of the mandibular landmarks in a pediatric population. Med Oral Patol Oral Cir Bucal. 2014;19:e136-41.

Cevidanes LH, Alhadidi A, Paniagua B, Styner M, Ludlow J, Mol A, et al. Three-dimensional quantification of mandibular asymmetry through cone-beam computerized tomography. Oral Surg Oral Med Oral Pathol Oral Radiol Endod. 2011;111:757-70.

Ceyhanli KT, Celik D, Altintas SH, Tasdemir T, O SS. Conservative treatment and follow-up of type III dens invaginatus using cone beam computed tomography. J Oral Sci. 2014;56:307-10.

Chindasombatjaroen J, Poomsawat S, Klongnoi B. Calcifying cystic odontogenic tumor associated with other lesions: case report with cone-beam computed tomography findings. Oral Surg Oral Med Oral Pathol Oral Radiol. 2012;113:414-20.

Cho KM, Jang JH, Park SH. Clinical management of a fused upper premolar with supernumerary tooth: a case report. Restor Dent Endod. 2014;39:319-23.

Christell H, Birch S, Hedesiu M, Horner K, Ivanauskaite D, Nackaerts O, et al. Variation in costs of cone beam CT examinations among healthcare systems. Dentomaxillofac Radiol. 2012a;41:571-7.

Christell H, Birch S, Horner K, Rohlin M, Lindh C, SEDENTEXCT consortium. A framework for costing diagnostic methods in oral health care: an application comparing a new imaging technology with the conventional approach for maxillary canines with eruption disturbances. Community Dent Oral Epidemiol. 2012b;40:351-61.

Christou T, Kau CH, Abou-Kheir NS, Louis PJ. The use of three-dimensional evaluation in the management of a complex patient with mandibular fracture: a 5-year evaluation. J Craniofac Surg. 2014;25:e223-8.

Clarke P, Longridge N, Gartshore L. A multidisciplinary management of a type III dens invaginatus in a maxillary permanent canine. Eur Arch Paediatr Dent. 2016;17:131-6.

Cohenca N, Berg J. Diagnosis and conservative treatment of dens invaginatus type III using cone beam computed tomography: two case reports. Pediatr Dent. 2013;35:E33-7.

Cohenca N, Simon JH, Roges R, Morag Y, Malfaz JM. Clinical indications for digital imaging in dento-alveolar trauma. Part 1: traumatic injuries. Dent Traumatol. 2007;23:95-104.

de Souza Tolentino E, Centurion BS, Lima MC, Freitas-Faria P, Consolaro A, Sant'ana E. Ameloblastic fibro-odontoma: a diagnostic challenge. Int J Dent. 2010. 2010:pii: 104630.

Decolli Y, Nemtoi A, Susanu S, Haba D, Petcu A. A software tool used in 3D evaluation of the alveolar bone defect in bilateral cleft lip and palate patients. Rev Med Chir Soc Med Nat Iasi. 2014;118:841-6.

Demirtas O, Dane A, Yildirim E. A comparison of the use of cone-beam computed tomography and panoramic radiography in the assessment of pre-eruptive intracoronal resorption. Acta Odontol Scand. 2016;74:636-41.

Dharmani U, Rajput A, Chaudhary S, Talwar S, Verma M. Type III talon cusp and Type III B dens invaginatus occurring simultaneously in a mandibular lateral incisor. Gen Dent. 2014;62:e16-21.

Dobbyn LM, Morrison JF, Brocklebank LM, Chung LL. A survey of the first 6 years of experience with cone beam CT scanning in a teaching hospital orthodontic department. J Orthod. 2013;40:14-21.

Doğan MS, Callea M, Kusdhany LS, Aras A, Maharani DA, Mandasari M, Adiatman M, Yavuz I. The evaluation of root fracture with cone beam computed tomography (CBCT): an epidemiological study. J Clin Exp Dent. 2018;10:e41-8.

Dogramaci EJ, Rossi-Fedele G, McDonald F. Clinical importance of incidental findings reported on small-volume dental cone beam computed tomography scans focused on impacted maxillary canine teeth. Oral Surg Oral Med Oral Pathol Oral Radiol. 2014;118:e205-9.

Donaldson K, O'Connor S, Heath N. Dental cone beam CT image quality possibly reduced by patient movement. Dentomaxillofac Radiol. 2013;42:91866873.

Donizeth-Rodrigues C, Fonseca-Da Silveira M, Goncalves-De Alencar AH, Garcia-Santos-Silva MA, Francisco-De-Mendonca E, Estrela C. Three-dimensional images contribute to the diagnosis of mucous retention cyst in maxillary sinus. . Med Oral Patol Oral Cir Bucal. 2013;18:e151-7.

dos Santos Neto P, dos Santos L, Coletta RD, Laranjeira AL, de Oliveira Santos CC, Bonan PR, et al. Imaging evalution of the gingival fibromatosis and dental abnormalities syndrome. Dentomaxillofac Radiol. 2011;40:236-43.

Drage N, Rogers S, Greenall C, Playle R. Incidental findings on cone beam computed tomography in orthodontic patients. J Orthod. 2013;40:29-37.

Durack C, Patel S. The use of cone beam computed tomography in the management of dens invaginatus affecting a strategic tooth in a patient affected by hypodontia: a case report. Int Endod J. 2011;44:474-83.

Eggers G, Muhling J, Hofele C. Clinical use of navigation based on cone-beam computer tomography in maxillofacial surgery. Br J Oral Maxillofac Surg. 2009;47:450-4.

Esmaeilzadeh M, Donyavi Z, Shokri A. Cone-beam computed tomography study of crown dilaceration with a talon cusp in an unerupted permanent maxillary tooth. J Craniofac Surg. 2016;27:e170-2.

EzEldeen M, Van Gorp G, Van Dessel J, Vandermeulen D, Jacobs R. 3-dimensional analysis of regenerative endodontic treatment outcome. J Endod. 2015;41:317-24.

Feng B, Jiang M, Xu X, Li J. A new method of volumetric assessment of alveolar bone grafting for cleft patients using cone beam computed tomography. Oral Surg Oral Med Oral Pathol Oral Radiol.2017;124(2):e171-e82.

Friedrich RE, Scheuer HA, Grobe A. Anterior lingual mandibular bone depression in an 11-year-old child. In Vivo. 2012;26:1103-7.

Gandiban K, Ramakrishnan M. CBCT analysis of root canal morphology in geminated primary incisor: a proposed classification and case report. Gen Dent. 2014;62:44-6.

Ge ZP, Ma RH, Li G, Zhang JZ, Ma XC. Age estimation based on pulp chamber volume of first molars from cone-beam computed tomography images. Forensic Sci Int. 2015;253:133.e1-7.

Ge ZP, Yang P, Li G, Zhang JZ, Ma XC. Age estimation based on pulp cavity/chamber volume of 13 types of tooth from cone beam computed tomography images. Int J Legal Med. 2016;130:1159-67.

Ghoneima A, Sachdeva K, Hartsfield J, Weaver D, Kula K. The use of cone beam computed tomography for the assessment of trichorhinophalangeal syndrome, type I-a case report. J Orthod. 2013;40:47-52.

Goodell KB, Mines P, Kersten DD. Impact of cone-beam computed tomography on treatment planning for external cervical resorption and a novel axial slice-based classification system. J Endod. 2018;44:239-44.

Gruszka K, Rozylo TK, Rozylo-Kalinowska I, Denkiewicz K, Maslowska K. Transmigration of mandibular canine - case report. Pol J Radiol. 2014;79:20-3.

Guo J, Vahidnia A, Sedghizadeh P, Enciso R. Evaluation of root and canal morphology of maxillary permanent first molars in a North American population by cone-beam computed tomography. J Endod. 2014;40:635-9.

Gurgel CV, Costa AL, Kobayashi TY, Rios D, Silva SM, Machado MA, et al. Cone beam computed tomography for diagnosis and treatment planning of supernumerary teeth. Gen Dent. 2012;60:e131-5.

Hamada Y, Kondoh T, Noguchi K, Iino M, Isono H, Ishii H, Mishima A, Kobayashi K, Seto K. Application of limited cone beam computed tomography to clinical assessment of alveolar bone grafting: a preliminary report. Cleft Palate Craniofac J. 2005;42:128-37.

Haney E, Gansky SA, Lee JS, Johnson E, Maki K, Miller AJ, Huang JC. Comparative analysis of traditional radiographs and cone-beam computed tomography volumetric images in the diagnosis and treatment planning of maxillary impacted canines. Am J Orthod Dentofacial Orthop. 2010;137:590-7.

Hashim HA, Al-Qahtani AA, Taha SM, Tharupeedikayil S, Ahmed MF. Management of complete impacted maxillary second deciduous molar with the aid of cone-beam computed tomography: Case report and a review of the literature. J Orthod Sci 2013;2:130-5.

Hidalgo Rivas JA, The diagnostic efficacy of cone beam computed tomography for dental root fractures in non-endodontically treated anterior teeth. A systematic review. In: Aspects of Dental Cone Beam Computed Tomography In Children And Young People. PhD thesis, University of Manchester; 2014.

Hidalgo-Rivas JA, Theodorakou C, Carmichael F, Murray B, Payne M, Horner K. Use of cone beam CT in children and young people in three United Kingdom dental hospitals. Int J Paediatr Dent.2014;24:336-48.

Holst AI, Hirschfelder U, Holst S. Diagnostic potential of 3D-data-based reconstruction software: an analysis of the rare disease pattern of cherubism. Cleft Palate Craniofac J. 2009;46:215-9.

Hou M, Zhang LC, Zhang XZ, Song DL, Du QX, Liu CM. Application of the cone beam computed tomography (CBCT) in Le Fort I osteotomy. Zhonghua Zheng Xing Wai Ke Za Zhi. 2011;27:246-9.

Hunter AK, Muller S, Kalathingal SM, Burnham MA, Moore WS. Evaluation of an ameloblastic fibro-odontoma with cone beam computed tomography. Texas Dent J. 2012;129:619-24.

Ikeda K, Kawamura A, Ikeda R. Assessment of optimal condylar position in the coronal and axial planes with limited cone-beam computed tomography. J Prosthodont. 2011;20:432-8.

Isman O, Yilmaz HH, Aktan AM, Yilmaz B. Indications for cone beam computed tomography in children and young patients in a Turkish subpopulation. Int J Paediatr Dent.2017;27:183-90.

Jacobs R. Dental cone beam CT and its justified use in oral health care. JBR-BTR. 2011;94:254-65.

Janssen NG, Schreurs R, Bittermann GKP, Borstlap WA, Koole R, Meijer GJ, Maal TJJ. A novel semi-automatic segmentation protocol for volumetric assessment of alveolar cleft grafting procedures. J Craniomaxillofac Surg. 2017;45:685-689.

Jawad Z. A review of cone beam computed tomography for the diagnosis of root resorption associated with impacted canines, introducing an innovative root resorption scale. Oral Surg Oral Med Oral Pathol Oral Radiol.2016;122:765-71.

Jeremias F, Fragelli CM, Mastrantonio SD, Dos Santos-Pinto L, Dos Santos-Pinto A, Pansani CA. Cone-beam computed tomography as a surgical guide to impacted anterior teeth. Dent Res J (Isfahan).. 2016;13:85-9.

Jiang M, You M, Wang H, Xu L. Characteristic features of the adenomatoid odontogenic tumour on cone beam CT. Dentomaxillofac Radiol. 2014;43:20140016.

Kaneko T, Sakaue H, Okiji T, Suda H. Clinical management of dens invaginatus in a maxillary lateral incisor with the aid of cone-beam computed tomography--a case report. Dent Traumatol. 2011;27:478-83.

Katheria BC, Kau CH, Tate R, Chen JW, English J, Bouquot J. Effectiveness of impacted and supernumerary tooth diagnosis from traditional radiography versus cone beam computed tomography. Pediatr Dent. 2010;32:304-9.

Kato H. Non-surgical endodontic treatment for dens invaginatus type III using cone beam computed tomography and dental operating microscope: a case report. Bull Tokyo Dent Coll. 2013;54:103-8.

Kaya S, Yavuz I, Uysal I, Akkus Z. Measuring bone density in healing periapical lesions by using cone beam computed tomography: a clinical investigation. J Endod. 2012;38:28-31.

Keightley AJ, Cross DL, McKerlie RA, Brocklebank L. Autotransplantation of an immature premolar, with the aid of cone beam CT and computer-aided prototyping: a case report. Dent Traumatol. 2010;26:195-9.

Kfir A, Telishevsky-Strauss Y, Leitner A, Metzger Z. The diagnosis and conservative treatment of a complex type 3 dens invaginatus using cone beam computed tomography (CBCT) and 3D plastic models. Int Endod J. 2013;46:275-88.

Kim MS, Lee HS, Nam OH, Choi SC. Autotransplantation: A Reliable Treatment Modality for Severely Malpositioned Teeth. J Clin Pediatr Dent. 2017;41:388-91.

Kim SH, Choi YK, Shin SM, Choi YS, Yamaguchi T, Takahashi M, Maki K, Park SB, Kim YI. The estimation of skeletal maturity of patients with cleft lip and palate using statistical shape analysis: a preliminary study. Dentomaxillofac Radiol. 2017;46:20160491.

Kim SY, Choi SC, Chung YJ. Management of the fused permanent upper lateral incisor: a case report. Oral Surg Oral Med Oral Pathol Oral Radiol Endod. 2011;111:649-52.

Kleinbergen YJ, Schepers RH, Schepman KP. Unrecognizable objects on a radiograph after a dental trauma. Cone beam computer tomography provided clarification. Ned Tijdschr Tandheelkd. 2011;118:317-9.

Kobayashi TY, Gurgel CV, Cota AL, Rios D, Machado MA, Oliveira TM. The usefulness of cone beam computed tomography for treatment of complex odontoma. Eur Arch Paediatr Dent. 2013;14:185-9.

Kovisto T, Ahmad M, Bowles WR. Proximity of the mandibular canal to the tooth apex. J Endod. 2011;37:311-5.

Koye V, Grondahl HG. Characteristics of patients referred for Cone Beam Computed Tomography (CBCT) of ectopically erupting maxillary canines. Swed Dent J 2011;35:159-65.

Kuijpers MA, Pazera A, Admiraal RJ, Berge SJ, Vissink A, Pazera P. Incidental findings on cone beam computed tomography scans in cleft lip and palate patients Clin Oral Investig. 2014;18:1237-44.

Laffranchi L, Dalessandri D, Fontana P, Visconti L, Sapelli P. Cone beam computed tomography role in diagnosis and treatment of impacted canine patient's: a case report. Minerva Stomatol. 2010a;59:363-76.

Laffranchi L, Dalessandri D, Tonni I, Paganelli C. Use of CBCT in the orthodontic diagnosis of a patient with pycnodysostosis. Minerva Stomatol. 2010b;59:653-61.

Law CS, Douglass JM, Farman AG, White SC, Zeller GG, Lurie AG, Goske MJ. The image gently in dentistry campaign: partnering with parents to promote the responsible use of x-rays in pediatric dentistry. Pediatr Dent. 2014;36:458-9.

Lee D, Atti E, Blackburn J, Yen S, Lee D, Tetradis S, Hong C. Volumetric assessment of cleft lip and palate defects using cone beam computed tomography. J Calif Dent Assoc. 2013;41:813-7.

Lee Y, Chang SW, Perinpanayagam H, Yoo YJ, Lim SM, Oh SR, Gu Y, Ahn SJ, Kum KY. Autotransplantation of mesiodens for missing maxillary lateral incisor with cone-beam CT-fabricated model and orthodontics. Int Endod J. 2014;47:896-904.

Linderup BW, Kuseler A, Jensen J, Cattaneo PM. A novel semiautomatic technique for volumetric assessment of the alveolar bone defect using cone beam computed tomography. Cleft Palate Craniofac J. 2015;52:e47-55.

Linsuwanont P, Sinpitaksakul P, Lertsakchai T. Evaluation of root maturation after revitalization in immature permanent teeth with nonvital pulps by cone beam computed tomography and conventional radiographs. Int Endod J. 2017;50:836-46.

Liu DG, Zhang WL, Zhang ZY, Wu YT, Ma XC. Three-dimensional evaluations of supernumerary teeth using cone-beam computed tomography for 487 cases. Oral Surg Oral Med Oral Pathol Oral Radiol Endod. 2007;103:403-11.

Liu L, Ma L, Lin J, Zhang C, Jia Y. Assessing the Interdental Septal Thickness in Alveolar Bone Grafting Using Cone Beam Computed Tomography. Cleft Palate Craniofac J. 2016;53:683-9.

Lucey S, Heath N, Welbury RR, Wright G. Case report: Cone-beam ct imaging in the management of a double tooth. Eur Arch Paediatr Dent. 2009;10 Suppl 1:49-53.

Lucio PS, Cavalcante RB, Maia RN, Santos ES, Godoy GP. Aggressive ameloblastic fibro-odontoma assessment with CBCT and treatment Eur Arch Paediatr Dent. 2013;14:179-84.

MacDonald D. Cone-beam computed tomography and the dentist. J Investig Clin Dent. 2017;8(1).

Maini A, Durning P, Drage N. Resorption: within or without? The benefit of cone-beam computed tomography when diagnosing a case of an internal/external resorption defect. Br Dent J. 2008;204:135-7.

Mak K. Root Resorption Detection by Multiple Radiographs versus Cone-Beam Computed Tomography. [Master's thesis]. 2015 2015;1599860:21. https://digital.lib.washington.edu/researchworks/handle/1773/33709 Accessed 07 Apr 2019.

Marques-da-Silva B, Baratto-Filho F, Abuabara A, Moura P, Losso EM, Moro A. Multiple taurodontism: the challenge of endodontic treatment. J Oral Sci. 2010;52(4):653-8.

May JJ, Cohenca N, Peters OA. Contemporary management of horizontal root fractures to the permanent dentition: Diagnosis-radiologic assessment to include cone-beam computed tomography. J Endod. 2013;39(3 Suppl):S20-5..

Mehan W. The use of cone-beam computed tomography in the diagnosis and treatment of severely ectopic teeth. J Clin Orthod. 2007;41:701-4.

Mehrdad L, Malekafzali B, Shekarchi F, Safi Y, Asgary S. Histological and CBCT evaluation of a pulpotomised primary molar using calcium enriched mixture cement. Eur Arch Paediatr Dent. 2013;14:191-4.

Merrett SJ, Drage N, Siphahi SD. The use of cone beam computed tomography in planning supernumerary cases. J Orthod. 2013;40:38-46.

Mischkowski RA, Zinser MJ, Ritter L, Neugebauer J, Keeve E, Zoller JE. Intraoperative navigation in the maxillofacial area based on 3D imaging obtained by a cone-beam device. Int J Oral Maxillofac Surg. 2007;36:687-94.

Mittal P, Jadhav GR, Syed S, Bhujbal ND. Cone-Beam Computed Tomography-Guided Management of C-Shaped Type III Dens Invaginatus With Peri-invagination Periodontitis in a Maxillary Canine: A Case Report. Compend Contin Educ Dent. 2016;37:e9-e12.

Mossaz J, Kloukos D, Pandis N, Suter VG, Katsaros C, Bornstein MM. Morphologic characteristics, location, and associated complications of maxillary and mandibular supernumerary teeth as evaluated using cone beam computed tomography. Eur J Orthod. 2014;36:708-18.

Murphy M, Drage N, Carabott R, Adams C. Accuracy and reliability of cone beam computed tomography of the jaws for comparative forensic identification: a preliminary study. J Forensic Sci. 2012;57:964-8.

Nadig K, Newlands C, Minhas G. 3D printing assisted autotransplantation of an Ectopic Canine. British Journal of Oral and Maxillofacial Surgery. 2016 December;54 (10):e98.

Narayana P, Hartwell GR, Wallace R, Nair UP. Endodontic clinical management of a dens invaginatus case by using a unique treatment approach: a case report. J Endod. 2012;38:1145-8.

Nardi C, Borri C, Regini F, Calistri L, Castellani A, Lorini C, Colagrande S.. Metal and motion artifacts by cone beam computed tomography (CBCT) in dental and maxillofacial study. Radiol Med. 2015;120:618-26.

Nematolahi H, Abadi H, Mohammadzade Z, Soofiani Ghadim M. The use of cone beam computed tomography (CBCT) to determine supernumerary and impacted teeth position in pediatric patients: a case report. J Dent Res Dent Clin Dent Prospects. 2013;7:47-50.

Noffke CE, Farman AG, Nel S, Nzima N. Guidelines for the safe use of dental and maxillofacial CBCT: a review with recommendations for South Africa. SADJ. 2011;66:262, 4-6.

Nosrat A, Schneider SC. Endodontic management of a maxillary lateral incisor with 4 root canals and a dens invaginatus tract. J Endod. 2015;41:1167-71.

Nurko C. Three-dimensional imaging cone bean computer tomography technology: an update and case report of an impacted incisor in a mixed dentition patient. Pediatr Dent. 2010;32:356-60.

Oana L, Zetu I, Petcu A, Nemtoi A, Dragan E, Haba D. The essential role of cone beam computed tomography to diagnose the localization of impacted maxillary canine and to detect the austerity of the adjacent root resorption in the Romanian population. Rev Med Chir Soc Med Nat Iasi. 2013;117:212-6.

Oberoi S, Chigurupati R, Gill P, Hoffman WY, Vargervik K. Volumetric assessment of secondary alveolar bone grafting using cone beam computed tomography. Cleft Palate Craniofac J. 2009;46:503-11.

O'Connell S, Davies J, Smallridge J, Vaidyanathan M. Amelogenesis imperfecta associated with dental follicular-like hamartomas and generalised gingival enlargement. Eur Arch Paediatr Dent. 2014;15:361-8.

Olivieri JG, Duran-Sindreu F, Mercade M, Perez N, Roig M. Treatment of a perforating inflammatory external root resorption with mineral trioxide aggregate and histologic examination after extraction. J Endod. 2012;38:1007-11.

Omami M, Chokri A, Hentati H, Selmi J. Cone-beam computed tomography exploration and surgical management of palatal, inverted, and impacted mesiodens. Contemp Clin Dent. 2015;6(Suppl 1):S289-93.

Orhan AI, Orhan K, Aksoy S, Ozgul O, Horasan S, Arslan A, et al. Evaluation of perimandibular neurovascularization with accessory mental foramina using cone-beam
computed tomography in children. J Craniofac Surg. 2013;24:e365-9.

Orhan K, Uyanik LO, Erkmen E, Kilinc Y. Unusually severe limitation of the jaw attributable to fibrodysplasia ossificans progressiva: a case report with cone-beam computed tomography findings. Oral Surg Oral Med Oral Pathol Oral Radiol. 2012;113:404-9.

Ozcan G, Sekerci AE, Cantekin K, Aydinbelge M, Dogan S. Evaluation of root canal morphology of human primary molars by using CBCT and comprehensive review of the literature. Acta Odontol Scand. 2016a;74:250-8.

Ozcan G, Sekerci AE, Kocoglu F. C-shaped mandibular primary first molar diagnosed with cone beam computed tomography: A novel case report and literature review of primary molars' root canal systems. J Indian Soc Pedod Prev Dent. 2016b;34:397-404.

Pacheco-Pereira C, Alsufyani NA, Major MP, Flores-Mir C. Accuracy and reliability of oral maxillofacial radiologists when evaluating cone-beam computed tomography imaging for adenoid hypertrophy screening: a comparison with nasopharyngoscopy. Oral Surg Oral Med Oral Pathol Oral Radiol.2016;121:e168-74.

Pallivathukal RG, Misra A, Nagraj SK, Donald PM. Dens invaginatus in a geminated maxillary lateral incisor. BMJ Case Rep. 2015 May 22;2015. pii: bcr2015209672.

Pamboo J, Hans MK, Chander S, Kumar S, Chinna H. CBCT-aided multidisciplinary approach to salvaging an intruded tooth. Compend Contin Educ Dent. 2016;37:198, 200, 202 passim.

Park JM, Tatad JC, Landayan ME, Heo SJ, Kim SJ. Optimizing third molar autotransplantation: applications of reverse-engineered surgical templates and rapid prototyping of three-dimensional teeth. J Oral Maxillofac Surg. 2014;72:1653-9.

Patel S. The use of cone beam computed tomography in the conservative management of dens invaginatus: a case report. Int Endod J. 2010;43:707-13.

Pazera P, Bornstein MM, Pazera A, Sendi P, Katsaros C. Incidental maxillary sinus findings in orthodontic patients: a radiographic analysis using cone-beam computed tomography (CBCT). Orthod Craniofac Res. 2011;14:17-24.

Pinheiro LR, Pinheiro JJ, Junior SA, Guerreiro N, Cavalcanti MG. Clinical and imagiological findings of central giant cell lesion and cherubism. Braz Dent J. 2013;24:74-9.

Pliska B, DeRocher M, Larson BE. Incidence of significant findings on CBCT scans of an orthodontic patient population. Northwest Dent. 2011;90:12-6.

Pontual ML, Pontual AA, Grempel RG, Campos LR, Costa Ade L, Godoy GP. Aggressive multilocular osteoblastoma in the mandible: a rare and difficult case to diagnose. Braz Dent J. 2014;25:451-6.

Radwan A, Kim SG. Treatment of a hypertaurodontic maxillary second molar in a patient with 10 taurodonts: a case report. J Endod. 2014;40:140-4.

Reynolds M, Reynolds M, Adeeb S, El-Bialy T. 3-d volumetric evaluation of human mandibular growth. Open Biomed Eng J. 2011;5:83-9.

Rogers SA, Drage N, Durning P. Incidental findings arising with cone beam computed tomography imaging of the orthodontic patient. Angle Orthod. 2011;81:350-5.

Romero-Delmastro A, Kadioglu O, Currier GF, Cook T. Digital tooth-based superimposition method for assessment of alveolar bone levels on cone-beam computed tomography images. Am J Orthod Dentofacial Orthop. 2014;146:255-63.

Rouas P, Bandon D, Nancy J, Delbos Y, Hauret L, Bar D. Digital volume tomography using the NewTom system: advantages of this new technique in children. Arch Pediatr. 2006;13:1169-77.

Sahai S, Kaveriappa S, Arora H, Aggarwal B. 3-D imaging in post-traumatic malformation and eruptive disturbance in permanent incisors: a case report. Dent Traumatol. 2011;27:473-7.

Saini T, Ogunleye A, Levering N, Norton NS, Edwards P. Multiple enamel pearls in two siblings detected by volumetric computed tomography. Dentomaxillofac Radiol. 2008;37:240-4.

Sakabe R, Sakabe J, Kuroki Y, Nakajima I, Kijima N, Honda K. Evaluation of temporomandibular disorders in children using limited cone-beam computed tomography: a case report. J Clin Pediatr Dent. 2006;31:14-6.

Salehinejad J, Langaroodi AJ, Shahakbari R, Yazdani N. Ameloblastic fibrodentinoma: report of a rare case. J Contemp Dent Pract. 2013;14:548-51.

Sane VD, Chandan S, Patil S, Patil K. Cone Beam Computed Tomography Heralding New Vistas in Appropriate Diagnosis and Efficient Management of Incidentally Found Impacted Mesiodens. J Craniofac Surg. 2017;28:e105-e6.

Sansare K, Singh D, Sontakke S, Karjodkar F, Saxena V, Frydenberg M, Wenzel A. Should cavitation in proximal surfaces be reported in cone beam computed tomography examination? Caries Res. 2014;48:208-13.

Schneiderman ED, Xu H, Salyer KE. Characterization of the maxillary complex in unilateral cleft lip and palate using cone-beam computed tomography: a preliminary study. Journal of Craniofacial Surgery. 2009;20 Suppl 2:1699-710.

Schulze D, Blessmann M, Pohlenz P, Wagner KW, Heiland M. Diagnostic criteria for the detection of mandibular osteomyelitis using cone-beam computed tomography. Dentomaxillofac Radiol. 2006;35:232-5.

Shahbazian M, Jacobs R, Wyatt J, Denys D, Lambrichts I, Vinckier F, Willems G. Validation of the cone beam computed tomography-based stereolithographic surgical guide aiding autotransplantation of teeth: clinical case-control study. Oral Surg Oral Med Oral Pathol Oral Radiol. 2013;115:667-75.

Sharma S, Grover S, Sharma V, Srivastava D, Mittal M. Endodontic and esthetic management of a dilacerated maxillary central incisor having two root canals using cone beam computed tomography as a diagnostic aid. Case Rep Dent 2014;2014:861942.

Shim YS, Kim AH, Choi JE, An SY. Use of three-dimensional computed tomography images in dental care of children and adolescents in Korea. Technol Health Care. 2014;22:333-7.

Shirota T, Kurabayashi H, Ogura H, Seki K, Maki K, Shintani S. Analysis of bone volume using computer simulation system for secondary bone graft in alveolar cleft. Int J Oral Maxillofac Surg. 2010;39:904-8.

Sinanoglu A, Kocasarac HD, Noujeim M. Age estimation by an analysis of spheno-occipital synchondrosis using cone-beam computed tomography. Leg Med (Tokyo). 2016;18:13-9.

Soylu E, Alkan A, Dogan S, Ustun Y, Yildirim MD, Canpolat DG. Are radiological examinations necessary for mentally handicapped dental patients? J Clin Analytical Med. 2016;7:848-51.

Spin-Neto R, Matzen LH, Schropp L, Gotfredsen E, Wenzel A. Factors affecting patient movement and re-exposure in cone beam computed tomography examination. Oral Surg Oral Med Oral Pathol Oral Radiol.2015;119:572-8.

Spin-Neto R, Matzen LH, Schropp L, Gotfredsen E, Wenzel A. Movement characteristics in young patients and the impact on CBCT image quality. Dentomaxillofac Radiol. 2016;45:20150426.

Star H, Thevissen P, Jacobs R, Fieuws S, Solheim T, Willems G. Human dental age estimation by calculation of pulp-tooth volume ratios yielded on clinically acquired cone beam computed tomography images of monoradicular teeth. J Forensic Sci. 2011;56 Suppl 1:S77-82.

Stuehmer C, Essig H, Bormann KH, Majdani O, Gellrich NC, Rucker M. Cone beam CT imaging of airgun injuries to the craniomaxillofacial region. Int J Oral Maxillofac Surg. 2008;37:903-6.

Tan X, Zhang L, Zhou W, Li Y, Ning J, Chen X, Chen X, Song D, Zhou X, Huang D. Palatal Radicular Groove Morphology of the Maxillary Incisors: A Case Series Report. J Endod. 2017;43:827-33.

Teixido M, Abella F, Duran-Sindreu F, Moscoso S, Roig M. The use of cone-beam computed tomography in the preservation of pulp vitality in a maxillary canine with type 3 dens invaginatus and an associated periradicular lesion. J Endod. 2014;40:1501-4.

Terzic A, Combescure C, Scolozzi P. Accuracy of computational soft tissue predictions in orthognathic surgery from three-dimensional photographs 6 months after completion of surgery: a preliminary study of 13 patients. Aesthetic Plastic Surgery. [Comparative Study]. 2014;38(1):184-91.

Togan B, Gander T, Lanzer M, Martin R, Lubbers HT. Incidence and frequency of nondental incidental findings on cone-beam computed tomography. J Craniomaxillofac Surg. 2016;44:1373-80.

Trainito S, Favero L, Martini G, Pedersen TK, Favero V, Herlin T, Zulian F. Odontostomatologic involvement in juvenilel scleroderma of the face. J Paediatr Child Health. 2012;48:572-6.

Troeltzsch M, Liedtke J, Troeltzsch V, Frankenberger R, Steiner T, Troeltzsch M. Odontoma-associated tooth impaction: accurate diagnosis with simple methods? Case report and literature review. J Oral Maxillofac Surg. 2012;70:e516-20.

Van Acker JW, Martens LC, Aps JK. Cone-beam computed tomography in pediatric dentistry, a retrospective observational study. Clin Oral Investig. 2016;20:1003-10.

Vasconcelos Kde F, de-Azevedo-Vaz SL, Freitas DQ, Haiter-Neto F. CBCT Post-Processing Tools to Manage the Progression of Invasive Cervical Resorption: A Case Report. Braz Dent J. 2016;27:476-80.

Verweij JP, Anssari Moin D, Wismeijer D, van Merkesteyn JPR. Replacing Heavily Damaged Teeth by Third Molar Autotransplantation With the Use of Cone-Beam Computed Tomography and Rapid Prototyping. J Oral Maxillofac Surg. 2017;75:1809-16.

Verweij JP, Moin DA, Mensink G, Nijkamp P, Wismeijer D, van Merkesteyn JP. Autotransplantation of Premolars With a 3-Dimensional Printed Titanium Replica of the Donor Tooth Functioning as a Surgical Guide: Proof of Concept. J OralMaxillofac Surg. 2016;74:1114-9.

Vier-Pelisser FV, Morgental RD, Fritscher G, Ghisi AC, Borba MG, Scarparo RK. Management of type III dens invaginatus in a mandibular premolar: a case report. Braz Dent J. 2014;25:73-8.

Vier-Pelisser FV, Pelisser A, Recuero LC, So MV, Borba MG, Figueiredo JA. Use of cone beam computed tomography in the diagnosis, planning and follow up of a type III dens invaginatus case. Int Endod J. 2012;45:198-208.

Walker L, Enciso R, Mah J. Three-dimensional localization of maxillary canines with cone-beam computed tomography. Am J Orthod Dentofacial Orthop. 2005;128:418-23.

Wall A, Ng S, Djemal S. The value of cone beam CT in assessing and managing a dilated odontome of a maxillary canine. Dent Update. 2015;42:126-8.

Wang J, Cui NH, Guo YJ, Zhang W. Navigation-Guided Extraction of Impacted Supernumerary Teeth: A Case Report. J Oral Maxillofacl Surg. 2017;75:1136.e1-.e5.

Wangsrimongkol T, Manosudprasit M, Pisek P, Sutthiprapaporn P, Somsuk T. Alveolar bone graft evaluation agreement using cone beam computed tomography in cleft lip and palate patients: a pilot study. J Med Assoc Thai. 2013;96 Suppl 4:S36-43.

Wen C, Li G, Ren J, Zheng G. Evaluation of cone-beam CT in diagnosis of supernumerary teeth in the anterior maxilla. West China journal of stomatology. 2012;30:399-401.

Wenzel A. Radiographic display of carious lesions and cavitation in approximal surfaces: Advantages and drawbacks of conventional and advanced modalities. Acta Odontol Scand. 2014;72:251-64.

Wriedt S, Al-Nawas B, Schmidtmann I, Eletr S, Wehrbein H, Moergel M, Jacobs C. Analyzing the teeth next to the alveolar cleft: Examination and treatment proposal prior to bone grafting based on three-dimensional versus two-dimensional diagnosis-A diagnostic study. J Craniomaxillofac Surg. 2017;45:1272-7.

Yang R, Yang C, Liu Y, Hu Y, Zou J. Evaluate root and canal morphology of primary mandibular second molars in Chinese individuals by using cone-beam computed tomography. J Formos Med Assoc. 2013;112:390-5.

Yang Y, Xia X, Wang W, Qin M. Uncommon fusion of teeth and lateral periodontal cyst in a Chinese girl: a case report. Oral Surg Oral Med Oral Pathol Oral Radiol Endod. 2011;112:e18-20.

Yue W, Kim E. Nonsurgical Endodontic Management of a Molar-Incisor Malformation-affected Mandibular First Molar: A Case Report. J Endod. 2016;42:664-8.

Zhang MM, Liang YH, Gao XJ. Comparison of periapical radiography and cone-beam computed tomography in endodontic treated teeth for assessment of periapical lesions. Beijing Da Xue Xue Bao Yi Xue Ban. 2016;48:539-43.

Zhang W, Shen G, Wang X, Yu H, Fan L. Evaluation of alveolar bone grafting using limited cone beam computed tomography. Oral Surg Oral Med Oral Pathol Oral Radiol. 2012;113:542-8.

Zhou WN, Xu YB, Jiang HB, Wan L, Du YF. Accurate evaluation of cone-beam computed tomography to volumetrically assess bone grafting in alveolar cleft patients. J Craniofac Surg. 2015;26:e535-9.

Ziegler CM, Klimowicz TR. A comparison between various radiological techniques in the localization and analysis of impacted and supernumerary teeth. Indian J Dent Res. 2013;24:336-41.

Zoya A, Ali S, Alam S, Tewari RK, Mishra SK, Kumar A, Andrabi SM. Double Dens Invaginatus with Multiple Canals in a Maxillary Central Incisor: Retreatment and Managing Complications. J Endod. 2015;41:1927-32.
