# Supplementary material for: Diagnostic efficacy of cone beam computed tomography in paediatric dentistry: a systematic review
Source: Eur Arch Paediatr Dent. 2019 Dec 19;21(4):407–26. doi: 10.1007/s40368-019-00504-x (PMC7415745; doi:10.1007/s40368-019-00504-x)
Supplement: Supplementary file 4 — Supplementary material 4 (PDF 95 kb) [file 40368_2019_504_MOESM4_ESM.pdf]

ONLINE RESOURCE 4: CASE REPORT PUBLICATIONS CONTAINING FEWER THAN FIVE PAEDIATRIC CASES, LISTED ACCORDING TO THE CLINICAL CONTEXTS AND WITH A BRIEF INDICATION OF THE ROLE OF CBCT DEMONSTRATED IN THE REPORT.

Clinical contexts	Publication	Role of CBCT
Caries	NONE	
Acute infections	605. Schulze et al. (2006)	Imaging of the bony signs of osteomyelitis
Dental trauma	325. Cohenca et al. (2007)	Imaging of root fracture and luxation injuries
	1677. Kleinbergen et al. (2011)	Imaging of a luxation injury
	1209. Bahadure (2013)	Imaging of a post-trauma intruded incisor
	1693. Ambu et al. (2013)	Follow-up of replanted avulsed incisor
	275. Pamboo et al. (2017)	Imaging of a post-trauma intruded incisor
	452. Stuehmer et al. (2008)	Teeth displaced into facial soft tissues
	1721. Christou et al. (2014)	Mandibular fracture
Dental anomalies	12. Sahai et al. (2011)	Tooth malformation and eruptive disturbance
	998. Canoglu et al. (2012)	Management of unerupted macrodonts
	1031. Hashim (2013)	Management of unerupted primary molar
	255. Lucey et al. (2009)	3D model of geminated tooth used in treatment planning
	440. Laffranchi et al. (2010a)	Imaging of ectopic maxillary canine
	1645. Gruszka et al. (2014)	Imaging of ectopic mandibular canine
	1607. Nurko (2010)	Imaging of unerupted maxillary incisor and supernumerary tooth
	1698. Nematolahi et al. (2013)	Imaging of unerupted maxillary incisor and odontome
	257. Brauer (2011)	Imaging of multiple supernumerary teeth for surgical planning
	430. Gurgel et al. (2012)	Imaging of multiple supernumerary teeth for surgical planning
	1702. Merrett et al. (2013)	Imaging of supernumerary teeth and treatment planning
	478. Omami et al. (2015)	Imaging of multiple supernumerary teeth for surgical planning

	425. Al-Sehaibany et al. (2016)	Imaging of supernumerary teeth for surgical planning
	464. Jeremias et al. (2016)	Imaging of supernumerary teeth for surgical planning
	432. Sane et al. (2017)	Imaging of supernumerary teeth for surgical planning
	1146. Wang et al. (2017)	Navigation-guided extraction of impacted supernumerary teeth
	261. Gandiban and Ramakrishnan (2014)	Value in root canal identification in geminated tooth
	541. Ozcan et al. (2016)	Imaging of primary tooth root canal anomaly – no treatment done
	1674. Yang et al. (2011)	Value of imaging morphology of fused tooth
	1037. Kim (2013)	Value of imaging morphology of fused tooth
	326. Cho et al. (2014)	Value of imaging morphology of fused tooth
	1703. Patel (2010)	Value of imaging in endodontic management of dens invaginatus
	327. Kaneko et al. (2011)	Value of imaging in management of dens invaginatus
	717. Narayana et al. (2012)	Value of imaging in management of dens invaginatus
	1704. Vier-Pelisser et al. (2012)	Value of imaging in management of dens invaginatus
	594. Cohenca and Berg (2013)	Value of imaging in management of dens invaginatus
	1166. Kato (2013)	Value of imaging in management of dens invaginatus
	506. Ceyhanli et al. (2014)	Value of imaging in endodontic management of dens invaginatus
	1710. Teixido et al. (2014)	Value of imaging in endodontic management of dens invaginatus
	1038. Vier-Pelisser et al. (2014)	Value of imaging in endodontic management of dens invaginatus
	719. Nosrat and Schneider (2015)	Value of imaging in endodontic management of dens invaginatus
	551. Pallivathukal et al. (2015)	Value of imaging in endodontic management of dens invaginatus
	1739. Wall et al. (2015)	Value of imaging in endodontic management of dens invaginatus
	645. Zoya et al. (2015)	Value of imaging in endodontic management of dens invaginatus
	1365. Agrawal et al. (2016)	Value of imaging in endodontic management of dens invaginatus
	1126. Clarke et al. (2016)	Value of imaging in endodontic management of dens invaginatus
	489. Mittal et al. (2016)	Value of imaging in endodontic management of dens invaginatus
	715. Sharma, S.et al (2014)	Endodontic management of dilacerated tooth
	1165. Yue and Kim (2016)	Endodontic management of molar in case of Molar-Incisor Malformation
	483. Esmaeilzadeh et al. (2016)	Imaging of unerupted dilacerated tooth + talon cusp
	1672. Dharmani et al. (2014)	Imaging of talon cusp and dens invaginatus

	593. Kfir et al. (2013)	3D printed model in managing dens invaginatus
	1130. Saini et al. (2008)	Detection of enamel pearls
	1133. Marques-da-Silva et al. (2010)	Detection and imaging of taurodonts
	1725. Borges et al. (2014)	Detection and imaging of taurodonts
	1658. Radwan and Kim (2014)	Detection and imaging of taurodonts
	1722. Kobayashi et al. (2013)	Imaging of complex odontome
Developmental disorders	20. Reynolds et al. (2011)	3D models to assess growth of jaws
	612. Holst et al. (2009)	Imaging and monitoring of cherubism
	1694. Laffranchi et al. (2010b)	Imaging of pyknodysostosis
	1699. Ghoneima et al. (2013)	Imaging of trichorhinophalangeal syndrome, type Ia
Pathological conditions	91. Salehinejad et al. (2013)	Ameloblastic fibrodentinoma
	93. de Souza Tolentino et al. (2010)	Ameloblastic fibro-odontoma
	740. Hunter et al. (2012)	Ameloblastic fibro-odontoma: “extent of the lesion and its effects on adjacent structures”
	69. Lucio et al. (2013)	Ameloblastic fibro-odontoma: “extent of bone involvement”
	92. Augello et al. (2017)	Ameloblastic fibro-odontoma: “localisation of lesion and preservation of vital structures”
	94. O'Connell et al. (2014)	Amelogenesis imperfecta associated with dental follicular-like hamartomas and generalised gingival enlargement
	126. Friedrich et al. (2012)	Anterior mandibular lingual bone depression (Stafne)
	222. Boffano et al. (2012)	Buccal bifurcation cyst
	244. Chindasombatjaroen et al. (2012)	Calcifying cystic odontogenic tumour: extent and relationships of lesion
	254. Burak et al. (2018)	Coronoid hyperplasia
	272. Vasconcelos et al. (2016)	Invasive cervical resorption
	1693. Ambu et al. 2017	Inflammatory external root resorption

	1414. Maini (2008)	Differentiating internal and external resorption
	1738. Allison and Garlington (2017)	Dentigerous cyst
	312. Pinheiro et al. (2013)	Giant cell lesions
	1681. Orhan et al. (2012)	Fibrodysplasia ossificans progressiva
	1338. Brauer et al. (2013)	Odontogenic keratocyst
	70. Pontual et al. (2014)	Osteoblastoma:
Other uses	983. Mischkowski et al. (2007)	Surgical navigation using CBCT (foreign bodies; odontoma)
	801. Sakabe et al. (2006)	TMJ bony abnormality imaging
	213. Keightley et al. (2010)	3D model of tooth used for autotransplantation
	216. Lee.et al. (2014)	3D model of tooth used for autotransplantation
	1199. Park et al. (2014)	3D model of tooth used for autotransplantation
	217. Verweij et al. (2016)	3D model of tooth used for autotransplantation
	18. Nadig et al. (2016)	3D model of tooth used for autotransplantation
	1407. Verweij et al. (2017)	3D model of tooth used for autotransplantation
	218. Kim et al. (2017)	3D model of tooth used for autotransplantation
	22. EzEldeen et al. (2015)	Measurement of dental hard tissue volume in regenerative endodontics
	535. Adolphs et al. (2013)	Craniomaxillofacial surgery planning
	888. Mehrdad et al. (2013)	Evaluation of pulpotomy outcomes in primary molar
