# Supplementary material for: Diagnostic efficacy of cone beam computed tomography in paediatric dentistry: a systematic review
Source: Eur Arch Paediatr Dent. 2019 Dec 19;21(4):407–26. doi: 10.1007/s40368-019-00504-x (PMC7415745; doi:10.1007/s40368-019-00504-x)
Supplement: Supplementary file 5 — Supplementary material 5 (PDF 121 kb) [file 40368_2019_504_MOESM5_ESM.pdf]

ONLINE RESOURCE 5: Assessments of risk of bias of the diagnostic efficacy studies. **1.** diagnostic accuracy; **2**. diagnostic thinking efficacy and therapeutic efficacy; **3**. societal efficacy.

1. Assessments of risk of bias of the diagnostic accuracy efficacy studies, using QUADAS2 (Whiting et al. 2011). ☺ = low risk of bias or low level of concern about applicability. ? = unclear; ☹ = high risk of bias or concern about applicability.

Study	Clinical context	Risk of bias				Concerns regarding applicability		
		Patient selection	Index test	Reference standard	Flow and timing	Patient selection	Index test	Reference standard
Sansare et al. (2014)	Caries: *Diagnosis of approximal cavitation*	?	☺	☺	?	?	?	☺
Ziegler and Klimowicz (2013)	Dental anomalies: *localisation of unerupted teeth in the anterior maxilla*	☹	☹	☹	?	☺	☹	?
Mak (2015)	Pathological conditions: *Root resorption*	☹	☺	☹	?	?	☺	☺
Murphy et al. (2012)	Other uses: *Forensic identification*	?	?	☺	?	?	☺	☺

2: Assessments of risk of bias of the diagnostic thinking efficacy and therapeutic efficacy studies, using Modified QUADAS (Meads and Davenport, 2009). Study quality is presented a visual analogue scale, following the style of Horner and Shelley (2016). Studies marked with * appear under two clinical contexts.

Clinical context	Fryback and Thornbury level of efficacy		Study	Specific use of CBCT	Quality assessment
	Diagnostic thinking efficacy	Therapeutic efficacy			High quality ↔ Low quality
Dental trauma	X		Bornstein et al. (2009)	Diagnosis of horizontal root fracture position and angulation in permanent incisors	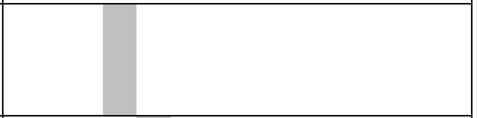
Dental anomalies	X	X	Haney et al. (2010)*	Diagnosis and treatment planning of impacted maxillary canines	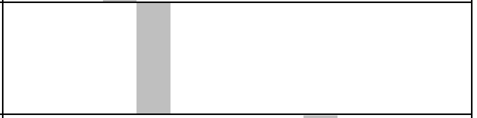
	X	X	Katheria et al. (2010)*	Diagnosis and treatment planning of impacted and supernumerary teeth	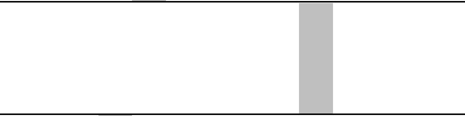
	X		Alqerban et al. (2011)*	Localisation and other imaging aspects of impacted maxillary canines	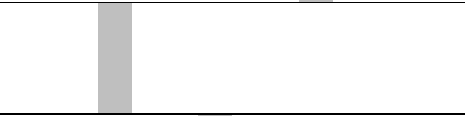
	X	X	Botticelli et al. (2011)	Diagnosis and treatment planning of impacted maxillary canines	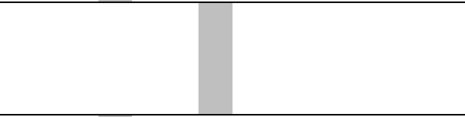
Developmental disorders	X	X	Wriedt et al. (2017)	Diagnosis and treatment plan related to teeth next to alveolar clefts and imaging of the cleft itself	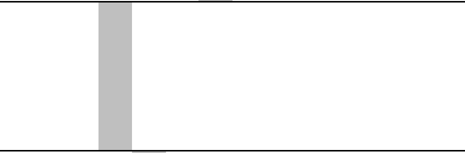
Pathological conditions	X		Haney et al. (2010)*	Diagnosis of root resorption in permanent incisors in relation to maxillary canine impaction	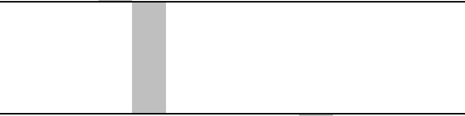
	X		Katheria et al. (2010)*	Diagnosis of root resorption in permanent incisors in relation to maxillary canine impaction	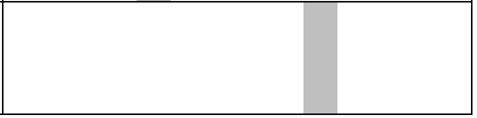

	X		Alqerban et al. (2011)*	Diagnosis of root resorption in permanent incisors in relation to maxillary canine impaction	
	X		Botticelli et al. (2011)	Diagnosis of root resorption in permanent incisors in relation to maxillary canine impaction	
	X		Jawad et al. (2016)	Diagnosis of root resorption in permanent incisors in relation to maxillary canine impaction	
		X	Goodell et al. (2018)	Treatment planning for external cervical resorption	

3: Quality assessments of the societal efficacy studies, using the proforma developed by Christell et al. (2014) and based on the checklist by Drummond (2005).

	Christell et al. (2012a)			Christell et al. (2012b)		
	Yes	No	Unclear	Yes	No	Unclear
SAMPLE SELECTION						
Is the sample selection relevant for the diagnostic methods?	X			X		
DIAGNOSTIC METHODS						
Is there a clear rationale described for the methods under comparison?	X			X		
Does one of the methods represent the current approach for the sample and clinical condition?			X	X		
Were the methods described in a way to permit replication?	X			X		
Were appropriate outcomes presented and were these calculated appropriately? Is the evaluation of the outcomes based on valid analyses?		X			X	
ECONOMIC EVALUATION						
Is the perspective of the study stated – e.g.healthcare or societal?	X			X		
Is the type of economic evaluation stated?	X			X		
Is there a presentation of all relevant costs related to the stated perspective?	X			X		
Is there a presentation of the methods for calculating the costs?	X			X		
COST AND CONSEQUENCES						
Were costs and consequences measured accurately in appropriate physical units (e.g. hours of equipment time, hours of maintenance, number of m2, number of worked clinic hours, time per examination, gained life-years) and valued appropriately?	X			X		
Was an analysis of incremental costs and consequences of the alternative methods performed?		X		X		
Were costs and consequences adjusted for differential timing – discounting?		X		X		
Was allowance made for uncertainty in the estimates of costs and consequences?	X			X		
