# Supplementary material for: Diagnostic efficacy of cone beam computed tomography in paediatric dentistry: a systematic review
Source: Eur Arch Paediatr Dent. 2019 Dec 19;21(4):407–26. doi: 10.1007/s40368-019-00504-x (PMC7415745; doi:10.1007/s40368-019-00504-x)
Supplement: Supplementary file 6 — Supplementary material 6 (PDF 252 kb) [file 40368_2019_504_MOESM6_ESM.pdf]

ONLINE RESOURCE 6: DETAILED EVALUATION OF THE RESULTS OF THE REVIEW ACCORDING TO THE CLINICAL CONTEXTS

Caries

Although three systematic reviews were available from the supplementary search, each providing an overview of the use of CBCT in paediatric dentistry and in orthodontics (De Vos et al. 2009; van Vlijmen et al. 2012; De Grauwe et al. 2019), none addressed evidence regarding caries. One *in vivo* diagnostic accuracy efficacy study was identified as part of the main review (Sansare et al. 2014), although this was conducted on adult patients and, as shown in Online Resource 5, there was a lack of clarity around patient selection and some concern about applicability. Nonetheless, the reviewers felt that this unique study, focused on diagnosis of cavitation of approximal caries lesions rather than presence or depth of disease, had relevance to the paediatric age group. Overall accuracy and sensitivity for cavitation were significantly higher for CBCT than for bitewing radiography, but with no significant difference in specificity. The authors concluded that CBCT examination was significantly more accurate than bitewing radiography for detecting cavitated proximal carious lesions in posterior teeth without restorations.

A narrative review by Abogazalah and Ando (2017) listed sixteen primary research studies of diagnostic accuracy using CBCT for caries on a range of tooth surfaces. All but one study they reviewed were *ex vivo/ in vitro* studies, with the exception being that of Sansare et al. (2014), reviewed above. This review concluded that CBCT did not improve the accuracy of *in vitro* caries detection when compared with intraoral radiography (analogue or digital). The current reviewers checked the source papers cited by Abogazalah and Ando (2017) and found that their statement had concealed some studies in which significant differences between diagnostic accuracy of CBCT and radiography had been reported, with some evidence for increased sensitivity for occlusal caries *ex vivo*, but not consistently so. The *ex vivo* study by Wenzel et al. (2013), cited by Abogazalah and Ando, 2017, found increased sensitivity for diagnosis of cavitation of proximal caries lesions, a finding confirmed by the *in vivo* study referred to above (Sansare et al. 2014).

A narrative review by Wenzel (2014) highlighted the evidence for improved diagnosis accuracy achievable using CBCT for proximal surface cavitation in the two studies

performed by her group (Wenzel et al. 2013; Sansare et al. 2014). She also, however, emphasised the problem of artefacts from high attenuation materials (metallic restorations, implants), and even from enamel itself, leading to incorrect diagnosis, along with the radiation dose impact and economic cost implications of using CBCT.

Online Resource 1 shows considerable alignment of guideline publications in the context of the use of CBCT and dental caries diagnosis. None recommends its use and many are emphatic in that CBCT should not be used for this purpose. The typical guidance is that CBCT should not be used as a primary means of assisting the task of caries diagnosis. Some add to this by stating that CBCT scans taken for other purposes should be evaluated for any caries lesions that may be identified as incidental findings.

Acute Dental Infections

For the purposes of this review, “acute dental infections” was interpreted as encompassing the use of CBCT in diagnosis of periapical inflammatory pathosis and in wider bony infection (i.e. osteomyelitis) although it is accepted that these conditions are often clinically encountered as chronic lesions.

From the supplementary broad search for systematic reviews, there were four systematic reviews of diagnostic efficacy of imaging techniques for periapical inflammatory pathosis (Petersson et al. 2012; Kruse et al. 2015; Leonardi Dutra et al. 2016; Aminoshariae et al. 2018). The review by Petersson et al. (2012) can fairly be said to have been superseded by the subsequent reviews, carried out when more primary studies had become available, so is not considered further here. These systematic reviews differed considerably in their review question and eligibility criteria. Kruse et al. (2015) concluded that higher sensitivity was achieved using CBCT than either periapical or panoramic radiography. One study (Balasundaram et al. 2012) was identified by them in which the additional use of CBCT in relation to treatment planning was investigated and found no significant difference in treatment choice was found when CBCT imaging was made available to endodontists. Kruse et al. (2015) also reported 13 clinical studies which essentially compared the numbers of periapical inflammatory lesions identified on CBCT and conventional radiographs, with no

diagnostic truth available. These indicated that when CBCT is used, more lesions are identified, but the additional yield varied a lot between studies.

The review by Leonardi Dutra et al. (2016) is a more formal and thorough systematic review, which considered studies of diagnostic accuracy for periapical inflammatory pathosis using conventional radiography (intraoral and panoramic radiography) and CBCT. This included five studies in which CBCT was included, all of which were *ex vivo/ in vitro* designs performed with dry mandibles and artificially produced periapical defects. They reported a remarkably high pooled sensitivity and specificity using CBCT imaging of 0.95 and 0.88, respectively, although two of the studies included in the meta-analysis had lower specificities (0.73 and 0.70, indicating a fairly high level of false positive diagnoses). The diagnostic odds ratio for CBCT was highest of the imaging techniques included, indicating better discriminatory test performance. As the review authors highlighted, however, these results apply to artificially created bone defects which do not have the same characteristics as pathosis. Aminoshariae et al. (2018) focused their review question on what the difference was in radiological outcome of non-surgical endodontics comparing CBCT imaging and periapical radiography. They found that the using CBCT doubled the odds of detecting a periapical lesion in outcome when compared with periapical radiographs.

Most existing guidelines (Online Resource 1) have nothing to say about the clinical context of acute dental infections. If periapical inflammatory pathosis is included under this heading, there is a consistent message from several guideline documents that CBCT might be indicated for diagnosis of dental periapical pathosis in patients who present with contradictory or nonspecific clinical signs and symptoms, who for those who have poorly localized symptoms.

<u>Dental trauma</u>

Tooth trauma proved to be the most fertile area for systematic reviews once the strict inclusion criteria of the review were relaxed to permit inclusion of *ex vivo/ in vitro* and non-paediatric reviews, with six identified by the supplementary search (Corbella et al. 2014; Long et al. 2014; Chang et al. 2016; Ma et al. 2016a; Talwar et al. 2016; Salinero et al. 2017). To these was added one other, a review in a PhD thesis, picked up in the main search through its PROSPERO registration but initially excluded because it was

based almost completely on *ex vivo/ in vitro* studies (Hidalgo Rivas et al. 2014). None of these reviews had eligibility criteria limiting inclusion to studies in the paediatric age group. Importantly, all reviews were aimed at root fracture diagnosis in permanent teeth and none at other aspects of dental trauma.

These systematic reviews varied in their focus and their eligibility criteria. In the context of our current review, related to paediatric use of CBCT and dental trauma, the study of Hidalgo Rivas et al. (2014) might be seen as potentially the most relevant, along with that of Ma et al. 2016a, as these looked at non-endodontically treated teeth, with the former specifically aimed at studies on single-rooted teeth. Trauma to non-endodontically treated anterior teeth is the most likely situation to be faced by a paediatric dentist. On the other hand, the reviews which had an eligibility criterion of solely *in vivo* studies (Long et al. 2014; Chang et al. 2016) also provide important information, taking into account that artefact from patient movement is included; in contrast, *ex vivo* studies may result in better image quality than seen clinically when movement is absent and the methods use often lack sufficient reproduction of soft tissues and bone. The most up-to-date systematic review (Salineiro et al. 2017) has the advantage of having more primary studies to review. Importantly, all reviews were aimed at root fracture diagnosis and none at other aspects of dental trauma. As some diagnostic accuracy studies have included a comparator imaging of periapical radiography, some reviews have presented data on this in addition to diagnostic accuracy using CBCT.

In terms of the standard measures of diagnostic accuracy (sensitivity, specificity, accuracy, positive and negative predictive values, diagnostic odds ratio, area under receiver operating characteristic curve) reviews vary in what is presented and also in the complexity of the way the results are presented, with some providing a metanalysis and others presenting only ranges of measurements or mean values. For non-root-filled teeth using CBCT, Salinero et al. (2017) reported a pooled sensitivity of 0.82 and a pooled specificity of 0.88, with a diagnostic odds ratio of 39.05. The figures for periapical radiography were a pooled sensitivity of 0.51 and a pooled specificity of 0.91, with a diagnostic odds ratio of 15.53. Their conclusion was therefore favourable to the use of CBCT for diagnosis of root fracture in non-root filled teeth, in accord with the findings and conclusions of previous reviews (Hidalgo Rivas et al. 2014; Long et

al. 2014; Ma et al. 2016). There is, however, repeated evidence from these reviews of reduced diagnostic accuracy in the presence of endodontic treatments and artefact-producing materials, particularly for vertical root fractures. Long et al. 2014 recommended caution when performing diagnosis of root fracture in endodontically treated teeth, while Chang, et al. 2016 and Ma et al. 2016 each found insufficient evidence for efficacy in this situation. The sophisticated meta-analysis performed by Talwar et al. 2016 found a trend for better diagnostic performance using CBCT imaging, compared with periapical radiographs, for vertical root fracture in non-endodontically treated teeth. If filling material was present in the canals, however, there was a significant fall in specificity using CBCT imaging and the diagnostic performance was found to be marginally better using periapical radiography. This was due to artefact from high attenuation materials leading to “streak” artefacts. It is important to note that lower specificity means an increased false positive diagnosis rate for fracture, which can have profound clinical consequences in terms of treatment choices.

As mentioned above, *in vivo* studies provide a different insight into diagnostic performance for an exacting diagnostic task like detection of root fracture, as the inevitable patient movement can be anticipated to degrade the image quality. Despite this Long et al. (2014), who only reviewed *in vivo* studies, performed a subgroup analysis on non-endodontically treated teeth studies and found that the diagnostic accuracy was still very high indeed, although no study in their review was carried out in the paediatric age group and limitations were noted about the small sample sizes and the reference standard diagnosis. Overall, looking at all the systematic reviews, *in vivo* evidence is based on thirteen studies, seven of which are only included in this review by Long et al. (2014). These seven studies are in Chinese and could not be accessed for checking by the current review team. Furthermore, all but one of the thirteen *in vivo* studies included teeth with endodontic treatment, so the evidence most relevant to the current review is lacking.

Hidalgo Rivas et al. (2014) found only one study at a higher level of diagnostic accuracy to review, an *in vivo* article on diagnostic thinking efficacy which was also picked up in our main search (Bornstein et al. 2009). That study evaluated the changes in diagnosis between the use of periapical radiographs and CBCT. The study was

judged as having only moderate quality because it relied on a single observer's assessments, with some lack of clarity about blinding, but the results suggested that the two imaging techniques led to different findings about fracture location on the tooth surface. Clearly, further studies at higher levels of diagnostic efficacy are required to see if this is a reproducible finding and if it translates to changes in management and, ideally, improved patient outcomes.

Apart from general review papers on CBCT that briefly mention the potential role of CBCT in dental trauma, one narrative review was identified which focused specifically on diagnosis of horizontal root fractures (May et al. 2013). While concluding that more clinical research was needed to assess impact on patient outcomes, they suggested that CBCT was of potential value in selected cases when conventional radiographic evidence was inconclusive. They particularly highlighted its appropriateness for fractures diagnosed as being in the middle third of the root, for which CBCT could exclude or confirm an oblique path into the coronal third that might influence retention or extraction. This suggestion was based principally on the study by Bornstein et al. (2009). Their paper also provides a suggested decision pathway for selecting CBCT when faced with a suspected horizontal root fracture.

Those guideline documents which make recommendations about CBCT in the context of dental trauma make essentially the same recommendation: that it should be considered in selected cases when conventional radiography is insufficient for diagnosis and treatment planning (Online Resource 1). The type of trauma mentioned in guidelines ranges from root fractures through to dento-alveolar fractures, but with an emphasis on the value of CBCT for more complex trauma rather than simpler dental trauma, despite the evidence being almost completely related to root fracture assessment. Two sets of guidelines (European Commission 2012; AWMF 2013) emphasise that "high resolution" CBCT is appropriate, with voxel sizes of 0.2mm or less specified in the former publication, although it is important to note that resolution is not wholly a function of voxel size.

Dental anomalies

For the purposes of this review, this category consisted of developmental anomalies of tooth number, size, structure, morphology and position. Odontomes were also included.

The supplementary search for systematic reviews identified a recent review (Eslami et al. 2017) on maxillary impacted canine localisation which included some *ex vivo/ in vitro* studies. This thorough systematic review made four conclusions: first, that using CBCT gave better accuracy for localisation of canines than using conventional radiography; second, that the wide variation in agreement between observers and between imaging modalities when localising canine teeth and in planning treatment might be due to methodological aspects of study design, including observer variation, and varying complexity of study cases/ subjects; third, as well as having better accuracy, the use of CBCT for localisation of maxillary canine teeth was more reliable. Finally, there was no robust evidence to support using CBCT as a first-line imaging method for imaging impacted maxillary canine teeth, but that it can be indicated when conventional radiographs fail to provide sufficient information. The review by De Grauwe et al. (2019) on CBCT use in the paediatric age group in orthodontics, highlights the value of CBCT in the context of impacted teeth and associated resorption, concluding that "CBCT can be considered justified in children for diagnosis and treatment planning of impacted teeth and root resorption". Beyond the use of CBCT in the context of impacted teeth, De Grauwe et al. (2019) stated that CBCT can be of great value for endodontic reasons in cases of dens invaginatus, fusion, or gemination.

The one diagnostic accuracy efficacy study identified by the main search in this category (Ziegler and Klimowicz 2013) related the anatomical localisation of unerupted teeth (including supernumerary teeth) in the anterior maxilla. The reference standard was the finding at surgery when the teeth were removed. The study was assessed as having a high risk of bias on multiple levels (Online Resource 5), but they reported correct preoperative diagnosis of anatomical location of the tooth using CBCT in all but two of 61 cases.

The four studies in the Diagnostic thinking efficacy category (Level 3) measured the impact of CBCT in the context of unerupted canines and supernumerary teeth (Haney et al. 2010; Katheria et al. 2010; Alqerban et al. 2011; Botticelli et al. 2011). These were studies from the orthodontic literature which, after careful consideration, were amongst those that were judged to still have relevance to the paediatric dentists. Haney et al. (2010) and Botticelli et al. (2011) found a change in diagnostic thinking as regards localisation of the canine in 16% and 35% of cases, respectively. Both studies found that a greater proportion of cases of associated root resorption were diagnosed using CBCT, with a change in diagnosis observed in 17.3% (Botticelli et al. 2011) and 37% (Haney et al. 2010) of cases. Alqerban et al. (2011) also identified more cases of resorption of incisor roots in association with impacted canine teeth when using CBCT. Their study can, however, be criticised for only using panoramic radiographs as comparator imaging. Most clinicians would view this as an incomplete examination to determine position of an unerupted canine and the presence or absence of incisor root resorption. Botticelli et al. (2011) reported improved estimation of the space conditions in the arch when using CBCT. The study of Katheria et al. (2010) was focused on the identification of impacted canines and supernumerary teeth and associated resorption in the anterior maxilla using CBCT and traditional radiographs (panoramic and maxillary occlusal). They concluded that CBCT provided more information than radiographs on the location of pathology, the presence of root resorption, and with regard to treatment planning. This study has weaknesses, reflected in the risk of bias assessment (Online Resource 5), however, including that the “radiographic” images were not radiographs at all, but cross-sectional images synthesised from the CBCT.

In terms of Level 4 (Therapeutic efficacy), three of the same studies addressed the impact of CBCT on treatment planning for unerupted maxillary canines compared with that based on conventional radiographic techniques. Haney et al. (2010) reported that use of CBCT images resulted in a change in treatment planning in 27% of teeth. This is remarkably similar to the findings of Botticelli et al. (2011), who reported a change in treatment plan for 29.5% of cases had when CBCT was available, with a shift towards proportionately fewer observational / interceptional approaches to treatment and “more active intervention”. This corresponded with a significantly increased judgement of cases as being “difficult” when CBCT was available.

The only Level 6 (Societal efficacy) studies that were identified by the search for this review was related to this clinical context, a study which developed a framework for costing diagnostic methods in oral health care and which was then applied the radiographic examination of maxillary canines with eruption disturbances (Christell et al. 2012a; Christell et al. 2012b). The comparison was between conventional imaging (panoramic and intraoral radiography) and panoramic radiography with CBCT. This study identified an incremental cost when CBCT was used, equivalent to a 57% increase in economic costs. A subsequent study by the same group, which was not picked up by the current search strategy because it was “orthodontic” in nature, performed a study that combined a cost analysis with a therapeutic efficacy design for evaluation of impacted maxillary canine teeth (Christell et al. 2018). They calculated an economic cost per changed treatment decision when using CBCT. While the absolute cost will vary considerably according to the setting (country, primary/ secondary care, operator profiles, time, etc), using CBCT as an addition or alternative to conventional radiography is associated with additional economic costs per changed treatment decision.

Out of the seven case series publications in this clinical context that underwent a detailed appraisal (those with five or more cases in the publication), three related to unerupted maxillary canine teeth (Walker et al. 2005; Koye and Grondahl 2011; Oana et al. 2013) and three to supernumerary teeth (Liu et al. 2007; Wen et al. 2012; Mossaz et al. 2014). In the case of maxillary canines suspected of a disturbance of eruption, several uses of CBCT were highlighted, but particularly its value for accurate localisation and assessment of angulation of the canine and the presence of resorption of roots in adjacent teeth. Essentially the same benefits of using CBCT were identified for supernumerary teeth. These case series did not, however, compare directly the diagnostic value of CBCT with that of conventional radiography. The seventh case series in the dental anomalies category presented eight cases of palatal radicular grooves in incisors, for which CBCT was proposed as the best way to identify the extent and severity of the groove prior to treatment decision regarding extraction or restoration outside the mouth and re-implantation (Tan et al. 2017).

There were numerous publications in the dental anomalies category reporting fewer than five cases (Online Resource 4). These covered a range of clinical uses, but prominent amongst them were cases showing the value of CBCT for endodontic management of dens invaginatus. Other publications made a case for the usefulness in other anomalous type of tooth development, including fusion, gemination, dilaceration and taurodonts. In some cases, however, it was difficult to understand why CBCT had been used for a reason relevant to management.

Many of the guidelines included in Online Resource 1 indicated that there is a role for CBCT in selected cases. These broadly fall into two groups of clinical situations, anomalies of tooth position (usually unerupted ectopic teeth and including supernumerary teeth) and, secondly abnormalities of tooth form (for example dens invaginatus, fusion). For the first type, this includes the need to detect or exclude resorption of adjacent teeth. The German guidelines emphasise that the most common abnormalities of the dentition (hypodontia) can be assessed radiologically using two-dimensional radiographs with sufficient accuracy (AWMF, 2013). Overall, most guidelines say, using various forms of words, that CBCT is indicated where the treatment approach cannot be established without further information in three dimensions. In the case of morphological anomalies of teeth, while several of the guidelines state there is a role for CBCT, they do not specify what purpose this serves, but others point to endodontic needs.

Developmental disorders

This category is formed of those developmental abnormalities of the maxillofacial region which are not classifiable as dental anomalies. In terms of the current review, this is essentially limited to developmental disorders affecting the bones of the jaws and face, because CBCT is an imaging technique of hard (calcified) tissues. Potentially, this could lead to inclusion of a large number of uncommon syndromes and conditions. It is perhaps unsurprising, however, that most evidence relating to CBCT in this category was in the context of the one of the most common developmental anomalies in this region: cleft lip and palate (CLP). Demonstrating the growth of the literature over the intervening years, De Grauwe et al. (2019) included 19 publications on the use of CBCT diagnosis for CLP patients. They concluded that CBCT was an excellent tool for assessing bone volume and morphology. Second, they

judged that it was helpful for assessing root morphology, development of the adjacent teeth close to the cleft area, and quantification of soft-tissue depth. Finally, they noted that it was feasible to use CBCT to measure outcome of bone grafting procedures. Many of the studies cited by De Grauwe et al. (2019), however, were merely using CBCT and had assumed rather than assessed its diagnostic efficacy.

The supplementary search for systematic reviews found three publications relevant to this clinical category from the orthodontic literature: two general reviews on the use of CBCT in orthodontics and one specifically on orofacial clefts but in the general context of three-dimensional imaging rather than solely on CBCT. The review by van Vlijmen et al. (2012) found no evidence at that time to show that use of “3D diagnostics” led to benefits to CLP patients, but anticipated that benefits might be demonstrated by future research. This publication was followed up in 2014 by the comprehensive systematic review by Kuijpers et al. (2014), which had the objective of identifying three-dimensional imaging methods that permit quantitative analysis of facial soft tissues, velopharyngeal function and airway, skeletal morphology, and dentition in CLP patients. The review undertook a careful quality assessment of publications. They concluded that for the assessment of the craniofacial skeleton, because CBCT generally has a lower radiation dose than CT, CBCT imaging might be the preferred method for quantifying bone volume and for surgical planning. However, they also qualified this by stating that further research studies were needed to establish how this imaging influenced treatment planning and treatment outcomes. For imaging the dentition, they noted the use of CBCT for imaging dental abnormalities, eruption and bone around the teeth in relation to clefts, but again pointed out the lack of evidence for impact on treatment planning.

One study (Wriedt et al. 2017) was identified at the diagnostic thinking and therapeutic efficacy levels by our search and was assessed as having a low risk of bias (Online Resource 5). This measured the impact of CBCT on treatment planning for patients with CLP. Its findings are a reminder that cross-sectional imaging does not automatically translate into improved diagnostic efficacy at the higher levels. They reported that orthodontists’ treatment proposals did not differ significantly using conventional radiographs or CBCT regarding possible alignment of teeth around the

cleft. They recommended, therefore, that CBCT could be justified only as a supplement to conventional radiographs in selected cases.

Several research studies which do not fit neatly into the diagnostic efficacy hierarchy, but which otherwise fitted with the eligibility criteria of the main search, were included in the “other research designs” category. All of these developed or investigated methods using CBCT data to characterise and quantify bone or, in the case of CLP, cleft defects (Cevidanes et al. 2011; Reynolds et al. 2011; Zhang et al. 2012; Linderup et al. 2017; Janssen et al. 2017).

Of the reviews identified in the main search, there is little mention of developmental disorders. Aps (2013) suggested that so-called ‘‘complex craniofacial and surgical cases” may be the most suitable candidates for CBCT imaging, although he emphasised that the need for CBCT required a ‘‘case-by-case” approach. Jacobs (2011) highlighted the potential value of CBCT in the context of maxillofacial surgery, as part of preoperative planning and its transfer to the surgical theatre. One aspect of this is that patients with disorders of the facial bones are subjected to repeated imaging, often using CT. In such instances, the imaging of the facial bones can almost always be achieved at a lower radiation dose using CBCT.

One larger cases series presented 84 cases of patients who had undergone Le Fort I osteotomy with CBCT available (Hou et al. 2011). They described how the imaging allowed an exact evaluation of important anatomical structures and concluded that CBCT improves the safety and accuracy of surgery. There is no evidence, however, given for the opinion about improved safety.

The role of CBCT in the management of syndromic patients barely exists, at least in terms of the results of our search, apart from a few case reports in the paediatric age group. Our screening process excluded cases in which CBCT had been used but without any comment on its value, so those that were included were few. Only one of these (dos Santos Neto et al. 2011) included sufficient cases that were large enough for more detailed assessment, presenting a single family with a new syndrome. Their comment was that *“CBCT was useful to characterize the dentomaxillofacial features*”, but this was opinion rather than based on empirical evidence. A few other single case

reports are available (Online Resource 4) but these tell us little about the usefulness of CBCT compared with conventional radiography.

Only three guideline publications (Online Resource 1) present statements for this clinical context. All indicate that CBCT may be appropriate for imaging of cleft palate and for other complex malformations of the jaws and facial skeleton. The European guidelines (European Commission 2012) highlight the potential advantages for cleft palate imaging of replacing CT by CBCT when the latter has a radiation dose advantage.

Pathological conditions

This clinical context is potentially enormous, encompassing dental pathosis not already in other categories, cysts (odontogenic and non-odontogenic), tumours (benign and malignant), metabolic and endocrine diseases with bony manifestations and many other lesions. This category yielded the second highest number of publications for review although, as with the review generally, this was dominated by case series and reports. Despite the potential for very many pathoses to be included, the topics that dominated this clinical category were periodontal diseases and resorptions of teeth.

Periodontal diseases

Although periodontal disease is primarily a disease of adults, some periodontal diseases can present in the paediatric population. Although they did not discuss periodontal uses of CBCT in the text of their paper, De Grauwe et al. (2019) mentioned that CBCT was not indicated except for cases of infrabony defects and furcation lesions. Using our supplementary search strategy, four systematic reviews looking specifically at the diagnostic efficacy for aspects of periodontal diseases were identified (Nikolic-Jakoba et al. 2016; Kim and Bassir 2017; Choi et al. 2018; Woelber et al. 2018). There are important differences in the aims and inclusion criteria of these reviews. All except of Choi et al. (2018) undertook some kind of assessment of risk of bias for studies included in their reviews, although one of these was not a detailed assessment (Kim and Bassir 2017). Although there is also a marked lack of detail about the results of their risk of bias assessments, the limitation to clinical studies by Woelber et al. (2018) provides the most relevant information for our current review.

They concluded that CBCT provided high accuracy for detecting periodontal bony defects, furcation involvements, the periodontal ligament space, and for imaging of bony defects in the bucco-lingual direction compared with conventional radiographs. In terms of impact on clinical practice, the reviewers found that there was some potential value of CBCT is cases of regenerative periodontal surgery and complex cases including treatment of furcation lesions. They emphasised, however, that there was a lack of evidence that using CBCT changed patient outcomes after treatment. Overall, it is notable that two of the systematic reviews reached starkly different conclusions; Nikolic-Jakoba et al. (2016) found insufficient scientific evidence to justify CBCT for the diagnosis of and/or treatment planning for intrabony and furcation defects while, in complete contrast, CBCT was judged to be "*the best imaging technique*" to assess infrabony defects and furcation lesions by Choi et al. (2018). The more moderate conclusions of Kim and Bassir (2017) and Woelber et al. (2018), that CBCT should be restricted to complex periodontal cases, particularly those involving maxillary molars including planning regenerative periodontal surgery, seems sensible.

Several guidelines (Online Resource 1) include recommendations relating to periodontal imaging using CBCT, but there is no sign that any paediatric use was anticipated. Those that make recommendations say that CBCT is not indicated as a routine method of imaging periodontal bone and that it might be indicated in very selected cases of infra-bony defects and furcation lesions, where clinical and conventional radiographic examinations do not provide the information needed for management. The only guideline document that is specifically prepared for periodontal practice also recommends highly selected use (Mandelaris et al. 2017).

Resorption of teeth

One systematic review was identified relating to the diagnostic role of CBCT in assessment of root resorption using the supplementary search strategy (Yi et al. 2018). They performed a meta-analysis of *ex vivo* studies that compared the diagnostic accuracies achievable using CBCT and periapical radiography for the detection of external root resorption *ex vivo*. Although the context was orthodontic-related resorption, the studies they reviewed included some from the endodontic literature. They reported that the pooled sensitivity using CBCT (0.89) was significantly higher than that achievable with periapical radiography (0.62), while there

was no significant difference in specificities (0.92 and 0.89 for CBCT and periapical radiography, respectively). They highlighted, however, the limitations of using *ex vivo* models and of the heterogeneity of the studies.

One diagnostic accuracy (Level 2) study satisfied the inclusion criteria for our review (Mak, 2015). She performed a small retrospective study of 34 patients $\leq$ 18 years with impacted teeth and who had CBCT and multiple conventional radiographs. Sensitivity for diagnosis of resorption was higher using CBCT. Unfortunately, the study was small and also scored as having a high risk of bias for the reference standard (Online Resource 5), which was based on the CBCT as viewed by an experienced radiology specialist. This problem of obtaining a valid reference standard is common to any *in vivo* study of diagnostic accuracy.

Two retrospective clinical studies were included as Diagnostic thinking studies, both in relation to detection of incisor root resorption associated with unerupted maxillary canines (Alqerban et al. 2011; Jawad et al. (2016). The first study included 60 cases with CBCT and panoramic radiographs (Alqerban et al. 2011). Eleven observers were asked to identify incisor resorption, its severity and location on the root. Considering the modal assessment of the observers, resorption was significantly more often identified in lateral incisors using CBCT than based on panoramic radiographs and the level of agreement between observers was greater with CBCT. Resorption severity was significantly different between the two imaging modalities. The current reviewers noted that there was no inclusion of intraoral radiographs in this study which, in view of their greater image detail, might have led to some improvement in diagnosis if it had been used instead of, or with, panoramic radiography. The limitations of panoramic radiography for detecting root resorption were highlighted by van Vlijmen et al. (2012).

The study by Jawad et al. (2016) made a retrospective review of consecutive cases of referrals for suspected root resorption in relation to impacted maxillary canines. They found that resorption was identified on only 19% of cases using plain radiographs but 63% when using CBCT. There are weaknesses in this study reflected in a high risk of bias (Online Resource 5), however, as almost two-thirds of the cases only had panoramic radiographs as the comparator rather than intraoral radiographs and the

two types of imaging were reported by only one individual who was not the same for the two types of imaging.

One study looked at the impact on treatment planning of CBCT information compared with conventional intraoral radiographs in cases of idiopathic cervical resorption of unknown patient age (Goodell et al. 2018). There was no significant difference between treatment decisions based on CBCT or radiographs either to undertake repair of lesions or not (i.e. a yes/no decision), but detailed treatment planning decisions differed in over half of cases. It should be noted that this paper presents data on diagnostic accuracy, which is inappropriate without any reference standard, although this did not impact on the risk of bias at the therapeutic efficacy level.

The case reports listed in Online Resource 4 include some which present examples of situations in which CBCT information aided diagnosis of various kinds of resorption of teeth.

Brief perusal of previous guidelines (Online Resource 1) reveals multiple references to the use of CBCT in the context of resorption associated with unerupted teeth, in endodontics, after dental trauma, and for differentiation of external from internal root resorption or invasive cervical resorption from other conditions. Most guidelines favour selected use of CBCT when considering patients with established or suspected resorptions for imaging, indicating that small field of view high-resolution CBCT should be chosen when the diagnostic information is not obtainable using conventional radiography (European Commission 2012; AWMF 2012; Faculty of General Dental Practice (UK) 2013; European Society of Endodontology 2014). Somewhat out of alignment with these statements, Dula et al. (2014) state that CBCT is appropriate in “all cases” of resorptions of various aetiologies in order to evaluate the possibility of saving the involved tooth. Similarly, AAE and AAOMR (2015) say that CBCT is the “the imaging modality of choice” in this situation, which implies routine use.

Cysts benign tumours and other benign conditions

It is notable that the main search found very little evidence on the diagnostic efficacy of CBCT in the context of benign jaw pathosis. No studies of diagnostic efficacy at any level were identified and the paediatric literature was dominated by case reports.

The recent review by De Grauwe et al. (2019) also found no publications for inclusion according to their PRISMA chart. Even in our wider supplementary search for systematic reviews, nothing specific was found on this subject. This paucity of evidence is initially surprising, although it is likely to reflect the challenges involved in running clinical trials, particularly in children, where randomisation of patients into CBCT and conventional imaging groups may be ethically difficult, when lesions may be quite rare and when the treatment choice is usually surgery regardless of the imaging.

Three case series included five or more patient cases to consider in more detail than the others listed in Online Resource 4. Jiang et al. (2014) presented eight cases of adenomatoid odontogenic tumour. They found that CBCT allowed a better assessment of the intralesional calcifications, the relationships with adjacent structures and the shape of the lesion. They said that the distribution of calcifications visible on CBCT provided information that was critical to radiological diagnosis. The calcification patterns of ameloblastic fibro-odontoma were different on CBCT and radiographs in a series of seven patients, leading the authors to suggest that CBCT information might be useful in differential diagnosis (Araki et al. 2016). In both these case series, a similarity is that both sets of authors highlight this usefulness in helping radiological differential diagnosis. It might be argued, however, that this would not make a difference to management in these cases.

Trainito et al. 2012 presented a series of sixteen young patients with juvenile localized scleroderma of the face. They found multiple dental and bony abnormalities on conventional panoramic, cephalometric radiography and using CBCT. It is not, however, clear what additional information was obtained by the CBCT apart from a three-dimensional set of information. They concluded by saying that CBCT may offer a feasible tool for monitoring disease progression, but that additional studies were needed.

Almeida-Barros et al. (2015) reviewed the imaging (CBCT and panoramic radiography) of 23 jaw tumours and the key radiological findings compared. They found that CBCT gave superior findings for clarity of the margins of lesions, relationships with teeth and other adjacent anatomical structures and bony expansion

and perforation. They concluded that panoramic radiography should not be the imaging modality of choice when evaluating tumours of the jaws. Looking at the lists of small case series and reports in Online Resource 4, a range of different pathoses of the jaws is included. One common element in these, in agreement with the views of Almeida-Barros et al. (2015), is that the value of CBCT is judged to be in determining the lesion boundaries, including the presence of perforation of the bone edges, and its relationships to important structures, notably neurovascular canals.

Despite the lack of empirical evidence of efficacy in the paediatric age group, some guidelines have recommended CBCT in the context of managing cysts and benign tumours of the jaws (AWMF 2013; Dula et al. 2014; Oenning et al. 2018). The German guidelines specify “major pathological changes” including “large” lesions. In contrast, the other two publications suggest more general use is justified in these situations, with one specifying the 6 to 15-year old age group (Oenning et al. 2018).
All three guidelines highlight the usefulness of CBCT in providing knowledge about the relationships to neighbouring important anatomical structures as part of surgical planning.

For the diagnosis of malignant bone tumours of the jaw region, MSCT must be applied instead in order to assess possible soft tissue infiltration and lymph node involvement. The Swiss guidelines specify that for malignant lesions, CBCT is not the appropriate first or sole choice of imaging and that CT/ MR is required (Dula et al. 2014).

<u>Other uses</u>
In carrying out this review, this additional category was used as a “catch-all” for clinical uses of CBCT which might be potentially relevant to the paediatric age group but which did not fall into the initial remit from EAPD. Three main areas were identified: endodontics, temporomandibular joint (TMJ), “other” oral surgical applications (not already covered in the main clinical categories) and forensic applications of CBCT.

Endodontics
Endodontic use contributed to this category, although some diagnostic uses have been mentioned within the the current review, in the context of diagnostic efficacy for

periapical pathosis, root fracture detection, in endodontic management of teeth with anomalous morphology (notably dens invaginatus) and root resorption.

Rosen et al. (2015) undertook a broad review of the use of CBCT in endodontics, classifying studies according the hierarchy of Fryback and Thornbury (1991). They found that 90% of included studies were at the two lowest levels of diagnostic efficacy and they opined that improvements in some technical characteristics of CBCT imaging, or in its diagnostic accuracy in a certain situations, may incorrectly lead to a conclusion that its use is a guarantee of improvements at higher levels of efficacy. Within the restraints of the inclusion criteria of the current review, no endodontic-related studies of diagnostic efficacy were found at any level.

Jacobs (2011) highlighted the use of CBCT in the context of complex root canal anatomy, those cases complicated by other factors such as resorptions, and in cases of endodontic treatment failure. Aps (2013) noted the impact of root filling materials on image quality in CBCT, which is a factor to consider when imaging teeth with radiopaque material in the roots.

Several large case series have reviewed root canal anatomy of specific tooth types in large populations by retrospective review of CBCT databases, including maxillary first permanent molars (Guo et al. 2014) and primary molars (Yang et al. 2013; Ozcan et al. 2016). While of interest to dental anatomists, endodontists and paediatric dentists, they do not provide evidence that CBCT information changes patient outcomes, particularly in the primary dentition. Imaging of root maturation after revitalisation procedures in immature permanent teeth using CBCT was described by Linsuwanont et al. (2017). CBCT provided detailed images of root morphology and canal contents, but there was no evidence presented of any clinical value outside the research context. EzEldeen et al. (2015), in an elegant study with preliminary in vitro validation, were able to use CBCT images of immature teeth undergoing regenerative endodontic treatments to quantify the increase of root length and volumetric change after treatment, as well as a detailed qualitative assessment of morphology. This technique is clearly demanding technically and not ready for regular clinical application.

When surveys of CBCT use in the paediatric age group are considered, it was rarely used for “endodontic reasons” in the UK survey by Hidalgo-Rivas et al. (2014) and never in the Turkish survey (Isman et al. 2017). The Belgian survey saw a higher rate of use, at 10% of the examinations in the survey, although most of these were related to a concurrent resorption (Van Acker et al. 2016). The overall impression obtained from these surveys was that endodontic use of CBCT in child patients is quite small.

Guidelines on the use of CBCT in endodontics are available from several specialist organisations and from general publications on dentistry. The review of the guidelines in Online Resource 1 gives the impression that this area of use of CBCT has probably received more attention, at least in terms of detailed guidance, than any other. Overall, guidelines advocate that CBCT is useful in selected cases, some of which might face a paediatric dentist, including complex root canal systems, re-treatment cases and those with complications such as fractured instruments, suspected perforations, etc. It is important, however, to note that none of these guidelines were specifically written with the paediatric age group in mind.

Once again, sparse evidence exists related to the application of CBCT in a specifically paediatric context. While high resolution CBCT images can allow detailed visualisation of root canal anatomy, there is no evidence that the diagnostic efficacy is better than using optical magnification or good quality periapical radiographs. Consequently, any recommendations on using CBCT for endodontic applications is based on largely case-based evidence and opinion.

Temporomandibular joint

Disease of the temporomandibular joint (TMJ) includes many different pathoses. In a paediatric population TMJ pathosis is less common than in adults but do occur.

The supplementary search for systematic reviews found three on aspects of diagnostic usefulness of CBCT in TMJ disorders (Hussain et al. 2008; Ma et al 2016b; Petersson 2012). Hussein et al. (1998) based on the limited *ex vivo* diagnostic accuracy evidence at the time, reported that that CBCT was a promising alternative to CT for diagnosis of erosions and osteophytes. What was essentially an update of that review but with enough new studies to allow a meta-analysis, looking at diagnostic accuracy for

detection of “osseous defects” of the TMJ, found that imaging using CBCT led to a pooled sensitivity of 0.67 and pooled specificity of 0.87. In the general review on justification of CBCT in a paediatric population by De Grauwe et al (2019), three primary studies on the TMJ were included in their review, although none was a diagnostic efficacy study and each would not have passed the eligibility criteria of the current review. They cited these studies to support the statement that CBCT was regarded as an accurate and reliable tool for the shape, volume and angulation of mandibular condyle. Ikeda et al (2011) performed a study on a small cohort of patients aged 12-25 years, to determine normal condyle position within the articular fossa. The underlying rationale seemed to be that this might reflect disc position although the potential clinical value of this knowledge is not apparent.

No diagnostic efficacy studies at any level were found through the main review. No larger case series were seen and only a single case report was found (Sakabe et al. (2006), showing that CBCT was able to image erosions and shape change in the condyle and improvement in these after treatment. Surveys of paediatric use of CBCT for TMJ imaging report very low levels of use for this purpose, with 1 to 1.5% of examinations identified in the three main surveys in the current review (Hidalgo-Rivas et al. 2014; Van Acker et al. 2016; Isman et al. 2017).

Despite the lack of evidence for efficacy in paediatric age groups, Aps (2013) suggested that CBCT might be a welcome diagnostic tool for evaluating the osseous components of the joint compared with CT because of the lower radiation dose.
Three guideline publications include statements related to the temporomandibular joint (European Commission 2012; AWMF 2013; Dula et al. 2014). These do not give strong support for the use of CBCT unless a bony pathosis is suspected. The most recent guidelines (Dula et al. 2014) state that “CBCT is not indicated for TMJ-related routine diagnosis in daily practice” and that the information it can give does not usually alter management, while the German guidelines emphasise that when imaging is needed to answer questions related to the soft tissue components of the joint, CBCT is not indicated (AWMF 2013).

In summary, management of TMJ problems forms a very limited part of the work of the paediatric dentist. Even in hospital environments, there is little use of CBCT to

image the joints, as indicated by survey evidence. The evidence is sparse for diagnostic efficacy of CBCT for the TMJ, particularly so for children, and its use has probably happened because it has a lower radiation dose than CT and, perhaps, easier availability.

Other Oral Surgical applications

Some applications of CBCT in Oral Surgery practice have been addressed in other clinical contexts, but others included orthognathic surgery planning, bone grafts and diagnosis of maxillofacial fractures. The importance of not using CBCT if soft tissue evaluation forms part of the diagnostic task and instead considering CT or MR imaging has been pointed out previously.

The most recent guideline publication mentions the use of CBCT in the paediatric age group as part of surgical planning for autotransplantation of teeth (Oenning et al 2018). Seven case studies were identified describing this procedure (Online Resource 4), along with a research study by Shahbazian et al. (2013). This interesting study used CBCT data to prepare tooth replicas and surgical guides for a cohort of 20 paediatric subjects undergoing autotransplantation. A control group was obtained by retrospective matching of old cases without the availability of CBCT data. They reported that the extra-alveolar time for the teeth was significantly shorter in the study group, on average being less than one minute compared with 3 to 10 minutes in the control group. While there was no statistically significant difference in outcomes between the two groups, more failures were seen in the controls.

Two guidelines suggest that CBCT can be indicated for localisation of foreign bodies (AWMF 2013; Dula et al 2014). Certainly, a radiopaque foreign body can be identified in soft tissues and some localisation achieved, but as CBCT has poor soft tissue contrast and shows tissue planes poorly, exact localisation might not be possible. Ultrasound, when available, can assist in localisation of foreign bodies without exposure to ionising radiation and without the requirement for radiopacity of the object in question.

Forensic uses

One of the four diagnostic accuracy efficacy studies identified by the main search strategy and included in the review was one aimed at identifying the accuracy and reliability achievable using CBCT for recording forensically relevant information in comparison with panoramic radiography (Murphy et al. 2012). As such, it was a borderline case for inclusion. The authors compared the identification of presence or absence of teeth including impacted teeth, dental restorations and any pathology. This study is weakened by using panoramic radiography as a reference standard. Clearly panoramic radiographs are not a "diagnostic truth", although using it as a reference standard is explicable when conventional radiography is the current imaging method. Findings identified as "false positives" and "negatives" using CBCT may have been true findings. The authors opined that CBCT might be useful for post-mortem identification, but a few disparities between it and panoramic radiography might require further investigation and that further research was needed.

Several studies have evaluated the use of CBCT images for dental age estimation (Star et al. 2011; Ge et al. 2015; Ge et al. 2016; Sinanoglu et al. 2016), with three of these studies focused on measurement of pulp volume as an indicator of age. The studies show a significant correlation between pulp volume and chronological age.

REFERENCES

Abogazalah N, Ando M. Alternative methods to visual and radiographic examinations for approximal caries detection. J Oral Sci. 2017;59:315-22.

Alqerban A, Jacobs R, Fieuws S, Willems G. Comparison of two cone beam computed tomographic systems versus panoramic imaging for localization of impacted maxillary canines and detection of root resorption. Eur J Orthod. 2011;33:93-102

Almeida-Barros RQ, Abilio VM, Yamamoto AT, Melo DP, Godoy GP, Bento PM. Digital panoramic radiography versus cone beam computed tomography in the delineation of maxillomandibular tumors. Gen Dent. 2015;63:e5-e10.

Altman DG, Bland JM. Absence of evidence is not evidence of absence. BMJ. 1995;311(7003):485.

Aps JK. Cone beam computed tomography in paediatric dentistry: overview of recent literature. Eur Arch Paediatr Dent. 2013;14:131-40.

AWMF (Arbeitsgemeinschaft der Wissenschaftlichen Medizinischen Fachgesellschaften). s2k-Leitlinie Dentale digitale Volumentomographie Version Nr. 9 vom 5 August 2013. AWMF-Register-Nummer:083–05. D¨usseldorf: AWMF; 2013. Available from: https://www.awmf.org/uploads/tx_szleitlinien/083-005l_S2k_Dentale_Volumentomographie_2013-10-abgelaufen.pdf. Accessed 5 April 2019.

American Academy of Pediatric Dentistry. Ad Hoc Committee on Pedodontic R. Guideline on prescribing dental radiographs for infants, children, adolescents, and persons with special health care needs. Pediatr Dent. 2012;34:189-91.

American Academy of Pediatric Dentistry. Prescribing Dental Radiographs for Infants, Children, Adolescents, and Individuals with Special Health Care Needs. Pediatr Dent. 2017;39:205-07.

Aminoshariae A, Kulild JC, Syed A. Cone-beam Computed Tomography Compared with Intraoral Radiographic Lesions in Endodontic Outcome Studies: A Systematic Review. J Endod. 2018;44:1626-31.

Aps JK. Cone beam computed tomography in paediatric dentistry: overview of recent literature. Eur Arch Paediatr Dent. 2013;14:131-40.

Araki M, Namaki S, Amemiya T, Matsumoto K, Honda K, Yonehara Y, Matsumoto N, Asano M. Diverse calcification patterns of ameloblastic fibro-odontoma on radiographic examination. J Oral Sci. 2016;58:533-537.

Balasundaram A, Shah P, Hoen MM, Wheater MA, Bringas JS, Gartner A, Geist JR. Comparison of cone-beam computed tomography and periapical radiography in predicting treatment decision for periapical lesions: a clinical study. Int J Dent. 2012;2012:920815.

Bornstein MM, Wolner-Hanssen AB, Sendi P, von Arx T. Comparison of intraoral radiography and limited cone beam computed tomography for the assessment of root-fractured permanent teeth. Dental Traumatol. 2009;25:571-7.

Botticelli S, Verna C, Cattaneo PM, Heidmann J, Melsen B. Two- versus three-dimensional imaging in subjects with unerupted maxillary canines. Eur J Orthod. 2011;33:344-9.

Cevidanes LH, Alhadidi A, Paniagua B, Styner M, Ludlow J, Mol A, Turvey T, Proffit WR, Rossouw PE. Three-dimensional quantification of mandibular asymmetry through cone-beam computerized tomography. Oral Surg Oral Med Oral Pathol Oral Radiol Endod. 2011;111:757-70.

Chang E, Lam E, Shah P, Azarpazhooh A. Cone-beam Computed Tomography for Detecting Vertical Root Fractures in Endodontically Treated Teeth: A Systematic Review. J Endod. 2016;42:177-85.

Choi IGG, Cortes ARG, Arita ES, Georgetti MAP. Comparison of conventional imaging techniques and CBCT for periodontal evaluation: A systematic review. Imaging Sci Dent. 2018;48:79-86.

Christell H, Birch S, Bondemark L, Horner K, Lindh C; SEDENTEXCT consortium. The impact of Cone Beam CT on financial costs and orthodontists' treatment decisions in the management of maxillary canines with eruption disturbance. Eur J Orthod. 2018;40:65-73.

Christell H, Birch S, Hedesiu M, Horner K, Ivanauskaite D, Nackaerts O, Rohlin M, Lindh C; SEDENTEXCT consortium. Variation in costs of cone beam CT examinations among healthcare systems. Dentomaxillofac Radiol. 2012a;41:571-7.

Christell H, Birch S, Horner K, Lindh C, Rohlin M; SEDENTEXCT Consortium. Economic evaluation of diagnostic methods used in dentistry. A systematic review. J Dent. 2014;42:1361-71.

Christell H, Birch S, Horner K, Rohlin M, Lindh C, SEDENTEXCT consortium. A framework for costing diagnostic methods in oral health care: an application comparing a new imaging technology with the conventional approach for maxillary canines with eruption disturbances. Community Dent Oral Epidemiol. 2012b;40:351-61.

Corbella S, Del Fabbro M, Tamse A, Rosen E, Tsesis I, Taschieri S. Cone beam computed tomography for the diagnosis of vertical root fractures: a systematic review of the literature and meta-analysis. Oral Surg Oral Med Oral Pathol Oral Radiol. 2014;118:593-602.

De Grauwe A, Ayaz I, Shujaat S, Dimitrov S, Gbadegbegnon L Van de Vannet B, Jacobs R. CBCT in orthodontics: a systematic review on justification of CBCT in a paediatric population prior to orthodontic treatment. Eur J Orthod. 2019;41:381-89.

De Vos W, Casselman J, Swennen GR. Cone-beam computerized tomography (CBCT) imaging of the oral and maxillofacial region: a systematic review of the literature. Int J Oral Maxillofac Surg. 2009;38:609-25.

Donaldson K, O'Connor S, Heath N. Dental cone beam CT image quality possibly reduced by patient movement. Dentomaxillofac Radiol. 2013;42:91866873.

dos Santos Neto P, dos Santos L, Coletta RD, Laranjeira AL, de Oliveira Santos CC, Bonan PR, Martelli-Júnior H. Imaging evalution of the gingival fibromatosis and dental abnormalities syndrome. Dentomaxillofac Radiol. 2011;40:236-43.

Drummond MF, Sculpher MJ, Torrance GW, O'Brien BJ, Stoddart GL. Methods for the economic evaluation of health care programmes. 3rd ed. Oxford: Oxford University Press; 2005.

Dula K, Bornstein MM, Buser D, Dagassan-Berndt D, Ettlin DA, Filippi A, Gabioud F, Katsaros C, Krastl G, Lambrecht JT, Lauber R, Luebbers HT, Pazera P, Türp JC; SADMFR. SADMFR guidelines for the use of Cone-Beam Computed Tomography/ Digital Volume Tomography. Swiss Dent J. 2014;124:1169-83.

Eslami E, Barkhordar H, Abramovitch K, Kim J, Masoud MI.Cone-beam computed tomography vs conventional radiography in visualization of maxillary impacted-canine localization: A systematic review of comparative studies. Am J Orthod Dentofacial Orthop. 2017;151:248-58.

European Commission. Radiation protection 172. Evidence based guidelines on cone beam CT for dental and maxillofacial radiology. Luxembourg: Office for Official Publications of the European Communities; 2012. https://ec.europa.eu/energy/sites/ener/files/documents/172.pdf. Accessed 5 September 2019.

European Society of Endodontology; Patel S, Durack C, Abella F, Roig M, Shemesh H, Lambrechts P, Lemberg K. European Society of Endodontology position statement: the use of CBCT in endodontics. Int Endod J 2014;47:502–4.

Evers S, Goossens M, de Vet H, van Tulder M, Ament A. Criteria list for assessment of methodological quality of economic evaluations: consensus on Health Economic Criteria. Int J Technol Assess Health Care 2005;21:240–5.

EzEldeen M, Van Gorp G, Van Dessel J, Vandermeulen D, Jacobs R. 3-dimensional analysis of regenerative endodontic treatment outcome. J Endod. 2015;41:317-24.

Faculty of General Dental Practice (UK). Horner K, Eaton KA, eds. Selection criteria for dental radiography. 3rd edn. London, UK: Faculty of General Dental Practice (UK) Royal College of Surgeons of Surgeons of England; 2013.

Fryback DG, Thornbury JR. The efficacy of diagnostic imaging. Med Decis Making. 1991;11:88-94.

Ge ZP, Ma RH, Li G, Zhang JZ, Ma XC. Age estimation based on pulp chamber volume of first molars from cone-beam computed tomography images. Forensic Sci Int. 2015;253:133.e1-7.

Ge ZP, Yang P, Li G, Zhang JZ, Ma XC. Age estimation based on pulp cavity/chamber volume of 13 types of tooth from cone beam computed tomography images. Int J Legal Med. 2016;130:1159-67.

Goodell KB, Mines P, Kersten DD. Impact of cone-beam computed tomography on treatment planning for external cervical resorption and a novel axial slice-based classification system. J Endod. 2018;44:239-44.

Guo J, Vahidnia A, Sedghizadeh P, Enciso R. Evaluation of root and canal morphology of maxillary permanent first molars in a North American population by cone-beam computed tomography. J Endod. 2014;40:635-9.

Haney E, Gansky SA, Lee JS, Johnson E, Maki K, Miller AJ, Huang JC. Comparative analysis of traditional radiographs and cone-beam computed tomography volumetric images in the diagnosis and treatment planning of maxillary impacted canines. Am J Orthod Dentofacial Orthop. 2010;137:590-7.

Hidalgo Rivas JA, The diagnostic efficacy of cone beam computed tomography for dental root fractures in non-endodontically treated anterior teeth. A systematic review. In: Aspects of Dental Cone Beam Computed Tomography in Children and Young People. PhD thesis, University of Manchester; 2014.

Hidalgo-Rivas JA, Theodorakou C, Carmichael F, Murray B, Payne M, Horner K. Use of cone beam CT in children and young people in three United Kingdom dental hospitals. Int J Paediatr Dent.2014;24:336-48.

Higgins JPT, Green S, editors. Cochrane Handbook for Systematic Reviews of Interventions Version 5.1.0 [updated March 2011]. The Cochrane Collaboration, 2011. http://www.handbook.cochrane.org. Accessed 5 September 2019.

Horner K, Islam M, Flygare L, Tsiklakis K, Whaites E. Basic principles for use of dental cone beam computed tomography: consensus guidelines of the European Academy of Dental and Maxillofacial Radiology. Dentomaxillofac Radiol. 2009;38:187-95.

Horner K, O'Malley L, Taylor K, Glenny AM. Guidelines for clinical use of CBCT: a review. Dentomaxillofac Radiol. 2015;44:20140225.

Horner K, Shelley AM. Preoperative radiological evaluation of missing single teeth: A review. Eur J Oral Implantol. 2016;9 Suppl 1:S69-88.

Hou M, Zhang LC, Zhang XZ, Song DL, Du QX, Liu CM. Application of the cone beam computed tomography (CBCT) in Le Fort I osteotomy. Zhonghua Zheng Xing Wai Ke Za Zhi. 2011;27:246-9.

Hussain AM, Packota G, Major PW, Flores-Mir C.Role of different imaging modalities in assessment of temporomandibular joint erosions and osteophytes: a systematic review. Dentomaxillofac Radiol. 2008;37:63-71.

Ikeda K, Kawamura A, Ikeda R. Assessment of optimal condylar position in the coronal and axial planes with limited cone-beam computed tomography. J Prosthodont. 2011;20:432-8.

Isman O, Yilmaz HH, Aktan AM, Yilmaz B. Indications for cone beam computed tomography in children and young patients in a Turkish subpopulation. Int J Paediatr Dent.2017;27:183-90.

Jacobs R. Dental cone beam CT and its justified use in oral health care. JBR-BTR. 2011;94:254-65.

Janssen NG, Schreurs R, Bittermann GKP, Borstlap WA, Koole R, Meijer GJ, Maal TJJ. A novel semi-automatic segmentation protocol for volumetric assessment of alveolar cleft grafting procedures. J Craniomaxillofac Surg. 2017;45:685-689..

Jawad Z. A review of cone beam computed tomography for the diagnosis of root resorption associated with impacted canines, introducing an innovative root resorption scale. Oral Surg Oral Med Oral Pathol Oral Radiol.2016;122:765-71.

Jiang M, You M, Wang H, Xu L. Characteristic features of the adenomatoid odontogenic tumour on cone beam CT. Dentomaxillofac Radiol. 2014;43:20140016.

Katheria BC, Kau CH, Tate R, Chen JW, English J, Bouquot J. Effectiveness of impacted and supernumerary tooth diagnosis from traditional radiography versus cone beam computed tomography. Pediatr Dent. 2010;32:304-9.

Kim DM, Bassir SH. When Is Cone-Beam Computed Tomography Imaging Appropriate for Diagnostic Inquiry in the Management of Inflammatory Periodontitis? An American Academy of Periodontology Best Evidence Review. J Periodontol. 2017;88:978-98.

Koye V, Grondahl HG. Characteristics of patients referred for Cone Beam Computed Tomography (CBCT) of ectopically erupting maxillary canines. Swed Dent J 2011;35:159-65.

Kruse C, Spin-Neto R, Wenzel A, Kirkevang LL. Cone beam computed tomography and periapical lesions: a systematic review analysing studies on diagnostic efficacy by a hierarchical model. Int Endod J. 2015;48:815-28.

Kuijpers MA, Chiu YT, Nada RM, Carels CE, Fudalej PS. Three-dimensional imaging methods for quantitative analysis of facial soft tissues and skeletal morphology in patients with orofacial clefts: a systematic review. PLoS One. 2014;9:e93442.

Law CS, Douglass JM, Farman AG, White SC, Zeller GG, Lurie AG, Goske MJ. The Image Gently in Dentistry campaign: partnering with parents to promote the responsible use of X-rays in pediatric dentistry. Pediatr Dent. 2014;36:458-9.

Leclercq E, Leeflang MM, van Dalen EC, Kremer LC. Validation of search filters for identifying pediatric studies in PubMed. J Pediatr. 2013;162:629-634.e2.

Leonardi Dutra K, Haas L, Porporatti AL, Flores-Mir C, Nascimento Santos J, Mezzomo LA, Corrêa M, De Luca Canto G. Diagnostic Accuracy of Cone-beam Computed Tomography and Conventional Radiography on Apical Periodontitis: A Systematic Review and Meta-analysis. J Endod. 2016;42:356-64.

Linderup BW, Kuseler A, Jensen J, Cattaneo PM. A novel semiautomatic technique for volumetric assessment of the alveolar bone defect using cone beam computed tomography. Cleft Palate Craniofac J. 2015;52:e47-55.

Linsuwanont P, Sinpitaksakul P, Lertsakchai T. Evaluation of root maturation after revitalization in immature permanent teeth with nonvital pulps by cone beam computed tomography and conventional radiographs. Int Endod J. 2017;50:836-46.

Liu DG, Zhang WL, Zhang ZY, Wu YT, Ma XC. Three-dimensional evaluations of supernumerary teeth using cone-beam computed tomography for 487 cases. Oral Surg Oral Med Oral Pathol Oral Radiol Endod. 2007;103:403-11.

Long H, Zhou Y, Ye N, Liao L, Jian F, Wang Y, Lai W. Diagnostic accuracy of CBCT for tooth fractures: a meta-analysis. J Dent. 2014;42:240-8.

Ludlow JB, Timothy R, Walker C, Hunter R, Benavides E, Samuelson DB, Scheske MJ. Effective dose of dental CBCT-a meta analysis of published data and additional data for nine CBCT units. Dentomaxillofac Radiol. 2015;44:20140197.

Ma RH, Ge ZP, Li G. Detection accuracy of root fractures in cone-beam computed tomography images: a systematic review and meta-analysis. Int Endod J. 2016a;49:646-54.

Ma RH, Yin S, Li G. The detection accuracy of cone beam CT for osseous defects of the temporomandibular joint: a systematic review and meta-analysis. Sci Rep. 2016b;6:34714.

Mak K. Root Resorption Detection by Multiple Radiographs versus Cone-Beam Computed Tomography. [Master's thesis]. 2015;1599860:21. https://digital.lib.washington.edu/researchworks/handle/1773/33709. Accessed 5 September 2019.

Mandelaris, G.A., Scheyer, E.T., Evans, M., Kim, D., McAllister, B., Nevins, M.L., Rios, H.F., and Sarment, D., American Academy of Periodontology Best Evidence Consensus Statement on Selected Oral Applications for Cone-Beam Computed Tomography. J Periodontol. 2017;88:939-45.

May JJ, Cohenca N, Peters OA. Contemporary management of horizontal root fractures to the permanent dentition: Diagnosis-radiologic assessment to include cone-beam computed tomography. J Endod. 2013;39(3 Suppl):S20-5..

Meads CA, Davenport CF. Quality assessment of diagnostic before-after studies: development of methodology in the context of a systematic review. BMC Med Res Methodol. 2009;9:3.

Moher D, Liberati A, Tetzlaff J, Altman DG, The PRISMA Group (2009). Preferred Reporting Items for Systematic Reviews and Meta-Analyses: The PRISMA Statement. BMJ 2009;339:b2535.

Mossaz J, Kloukos D, Pandis N, Suter VG, Katsaros C, Bornstein MM. Morphologic characteristics, location, and associated complications of maxillary and mandibular supernumerary teeth as evaluated using cone beam computed tomography. Eur J Orthod. 2014;36:708-18.

Murphy M, Drage N, Carabott R, Adams C. Accuracy and reliability of cone beam computed tomography of the jaws for comparative forensic identification: a preliminary study. J Forensic Sci. 2012;57:964-8.

Nardi C, Borri C, Regini F, Calistri L, Castellani A, Lorini C, Colagrande S.. Metal and motion artifacts by cone beam computed tomography (CBCT) in dental and maxillofacial study. Radiol Med. 2015;120:618-26.

Nikolic-Jakoba N, Spin-Neto R, Wenzel A. Cone-Beam Computed Tomography for Detection of Intrabony and Furcation Defects: A Systematic Review Based on a Hierarchical Model for Diagnostic Efficacy. J Periodontol. 2016;87:630-44.

Noffke CE, Farman AG, Nel S, Nzima N. Guidelines for the safe use of dental and maxillofacial CBCT: a review with recommendations for South Africa. SADJ. 2011;66:262, 4-6.

Oana L, Zetu I, Petcu A, Nemtoi A, Dragan E, Haba D. The essential role of cone beam computed tomography to diagnose the localization of impacted maxillary canine and to detect the austerity of the adjacent root resorption in the Romanian population. Rev Med Chir Soc Med Nat Iasi. 2013;117:212-6.

Oberoi S, Chigurupati R, Gill P, Hoffman WY, Vargervik K. Volumetric assessment of secondary alveolar bone grafting using cone beam computed tomography. Cleft Palate Craniofac J. 2009;46:503-11.

Oenning .C, Jacobs R, Pauwels R, Stratis A, Hedesiu M, Salmon B; DIMITRA Research Group, http://www.dimitra.be. Cone-beam CT in paediatric dentistry: DIMITRA project position statement. Pediatr Radiol 2018; 48, 308–316.

Ozcan G, Sekerci AE, Cantekin K, Aydinbelge M, Dogan S. Evaluation of root canal morphology of human primary molars by using CBCT and comprehensive review of the literature. Acta Odontol Scand. 2016a;74:250-8.

Petersson A. What you can and cannot see in TMJ imaging--an overview related to the RDC/TMD diagnostic system. J Oral Rehabil. 2010;37:771-8.

Petersson A, Axelsson S, Davidson T, Frisk F, Hakeberg M, Kvist T, Norlund A, Mejàre I, Portenier I, Sandberg H, Tranaeus S, Bergenholtz G. Radiological diagnosis of periapical bone tissue lesions in endodontics: a systematic review. Int Endod J. 2012;45:783-801.

Reynolds M, Reynolds M, Adeeb S, El-Bialy T. 3-d volumetric evaluation of human mandibular growth. Open Biomed Eng J. 2011;5:83-9.

Rosen E, Taschieri S, Del Fabbro M, Beitlitum I, Tsesis I. The Diagnostic efficacy of cone-beam computed tomography in endodontics: a systematic review and analysis by a hierarchical model of efficacy. J Endod. 2015;41:1008-14.

Sakabe R, Sakabe J, Kuroki Y, Nakajima I, Kijima N, Honda K. Evaluation of temporomandibular disorders in children using limited cone-beam computed tomography: a case report. J Clin Pediatr Dent. 2006;31:14-6.

Salineiro FCS, Kobayashi-Velasco S, Braga MM, Cavalcanti MGP. Radiographic diagnosis of root fractures: a systematic review, meta-analyses and sources of heterogeneity. Dentomaxillofac Radiol. 2017;46:20170400.

Sansare K, Singh D, Sontakke S, Karjodkar F, Saxena V, Frydenberg M, Wenzel A. Should cavitation in proximal surfaces be reported in cone beam computed tomography examination? Caries Res. 2014;48:208-13.

SIGN (Scottish Intercollegiate Guidelines Network). What we do. Methodology. Search Filters. https://www.sign.ac.uk/search-filters.html. Accessed 5 September 2019.

Shahbazian M, Jacobs R, Wyatt J, Denys D, Lambrichts I, Vinckier F, Willems G. Validation of the cone beam computed tomography-based stereolithographic surgical guide aiding autotransplantation of teeth: clinical case-control study. Oral Surg Oral Med Oral Pathol Oral Radiol. 2013;115:667-75.

Shea BJ, Reeves BC, Wells G, Thuku M, Hamel C, Moran J, Moher D, Tugwell P, Welch V, Kristjansson E, Henry DA. AMSTAR 2: a critical appraisal tool for systematic reviews that include randomised or non-randomised studies of healthcare interventions, or both. BMJ 2017; 21;358:j4008.

Sinanoglu A, Kocasarac HD, Noujeim M. Age estimation by an analysis of spheno-occipital synchondrosis using cone-beam computed tomography. Leg Med (Tokyo). 2016;18:13-9.

Spin-Neto R, Matzen LH, Schropp L, Gotfredsen E, Wenzel A. Factors affecting patient movement and re-exposure in cone beam computed tomography examination. Oral Surg Oral Med Oral Pathol Oral Radiol.2015;119:572-8.

Spin-Neto R, Matzen LH, Schropp L, Gotfredsen E, Wenzel A. Movement characteristics in young patients and the impact on CBCT image quality. Dentomaxillofac Radiol. 2016;45:20150426.

Star H, Thevissen P, Jacobs R, Fieuws S, Solheim T, Willems G. Human dental age estimation by calculation of pulp-tooth volume ratios yielded on clinically acquired cone beam computed tomography images of monoradicular teeth. J Forensic Sci. 2011;56 Suppl 1:S77-82.

Talwar S, Utneja S, Nawal RR, Kaushik A, Srivastava D, Oberoy SS. Role of cone-beam computed tomography in diagnosis of vertical root fractures: a systematic review and meta-analysis. J Endod.2016;42:12-24.

Tan X, Zhang L, Zhou W, Li Y, Ning J, Chen X, Chen X, Song D, Zhou X, Huang D. Palatal Radicular Groove Morphology of the Maxillary Incisors: A Case Series Report. J Endod. 2017;43:827-33.

Theodorakou C, Walker A, Horner K, Pauwels R, Bogaerts R, Jacobs R; SEDENTEXCT Project Consortium. Estimation of paediatric organ and effective doses from dental cone beam CT using anthropomorphic phantoms. Br J Radiol. 2012;85:153-60.

Trainito S, Favero L, Martini G, Pedersen TK, Favero V, Herlin T, Zulian F. Odontostomatologic involvement in juvenilel scleroderma of the face. J Paediatr Child Health. 2012;48:572-6.

van Acker JW, Martens LC, Aps JK. Cone-beam computed tomography in pediatric dentistry, a retrospective observational study. Clin Oral Investig. 2016;20:1003-10.

Walker L, Enciso R, Mah J. Three-dimensional localization of maxillary canines with cone-beam computed tomography. Am J Orthod Dentofacial Orthop. 2005;128:418-23.

Wriedt S, Al-Nawas B, Schmidtmann I, Eletr S, Wehrbein H, Moergel M, Jacobs C. Analyzing the teeth next to the alveolar cleft: Examination and treatment proposal prior to bone grafting based on three-dimensional versus two-dimensional diagnosis-A diagnostic study. J Craniomaxillofac Surg. 2017;45:1272-7.

van Vlijmen OJ, Kuijpers MA, Bergé SJ, Schols JG, Maal TJ, Breuning H, Kuijpers-Jagtman AM. Evidence supporting the use of cone-beam computed tomography in orthodontics. J Am Dent Assoc. 2012;143:241-52.

White SC, Scarfe WC, Schulze RK, Lurie AG, Douglass JM, Farman AG, Law CS, Levin MD, Sauer RA, Valachovic RW, Zeller GG, Goske MJ. The Image Gently in Dentistry campaign: promotion of responsible use of maxillofacial radiology in dentistry for children. Oral Surg Oral Med Oral Pathol Oral Radiol. 2014;118:257-61.

Wen C, Li G, Ren J, Zheng G. Evaluation of cone-beam CT in diagnosis of supernumerary teeth in the anterior maxilla. West China journal of stomatology. 2012;30:399-401.

Wenzel A. Radiographic display of carious lesions and cavitation in approximal surfaces: Advantages and drawbacks of conventional and advanced modalities. Acta Odontol Scand. 2014;72:251-64.

Wenzel A, Hirsch E, Christensen J, Matzen LH, Scaf G, Frydenberg M (2013) Detection of cavitated approximal surfaces using cone beam CT and intraoral receptors. Dentomaxillofac Radiol 42, 39458105.

Whiting PF, Rutjes AWS, Westwood ME, Mallett S, Deeks JJ, Reitsma JB, Leeflang MM, Sterne JAC, and Bossuyt PMM. QUADAS-2: A Revised Tool for the Quality Assessment of Diagnostic Accuracy Studies. Ann Intern Med. 2011;155:529-36.

Woelber JP, Fleiner J, Rau J, Ratka-Krüger P, Hannig C. Accuracy and usefulness of cbct in periodontology: a systematic review of the literature. Int J Periodontics Restorative Dent. 2018;38:289-97.

Yang R, Yang C, Liu Y, Hu Y, Zou J. Evaluate root and canal morphology of primary mandibular second molars in Chinese individuals by using cone-beam computed tomography. J Formos Med Assoc. 2013;112:390-5.

Yi J, Sun Y, Li Y, Li C, Li X, Zhao Z. Cone-beam computed tomography versus periapical radiograph for diagnosing external root resorption: A systematic review and meta-analysis. Angle Orthod. 2017;87:328-37.

Zhang W, Shen G, Wang X, Yu H, Fan L. Evaluation of alveolar bone grafting using limited cone beam computed tomography. Oral Surg Oral Med Oral Pathol Oral Radiol. 2012;113:542-8.

Zhou WN, Xu YB, Jiang HB, Wan L, Du YF. Accurate evaluation of cone-beam computed tomography to volumetrically assess bone grafting in alveolar cleft patients. J Craniofac Surg. 2015;26:e535-9.

Ziegler CM, Klimowicz TR. A comparison between various radiological techniques in the localization and analysis of impacted and supernumerary teeth. Indian J Dent Res. 2013;24:336-41.
